# Horizontally Transferred Salivary Protein Promotes Insect Feeding by Suppressing Ferredoxin-Mediated Plant Defenses

**DOI:** 10.1093/molbev/msad221

**Published:** 2023-10-07

**Authors:** Yi-Zhe Wang, Yu-Xuan Ye, Jia-Bao Lu, Xin Wang, Hai-Bin Lu, Ze-Long Zhang, Zhuang-Xin Ye, Yu-Wen Lu, Zong-Tao Sun, Jian-Ping Chen, Jun-Min Li, Chuan-Xi Zhang, Hai-Jian Huang

**Affiliations:** State Key Laboratory for Managing Biotic and Chemical Threats to the Quality and Safety of Agro-products, Key Laboratory of Biotechnology in Plant Protection of Ministry of Agriculture and Zhejiang Province, Institute of Plant Virology, Ningbo University, Ningbo, China; Institute of Insect Science, Zhejiang University, Hangzhou, China; State Key Laboratory for Managing Biotic and Chemical Threats to the Quality and Safety of Agro-products, Key Laboratory of Biotechnology in Plant Protection of Ministry of Agriculture and Zhejiang Province, Institute of Plant Virology, Ningbo University, Ningbo, China; State Key Laboratory for Managing Biotic and Chemical Threats to the Quality and Safety of Agro-products, Key Laboratory of Biotechnology in Plant Protection of Ministry of Agriculture and Zhejiang Province, Institute of Plant Virology, Ningbo University, Ningbo, China; State Key Laboratory for Managing Biotic and Chemical Threats to the Quality and Safety of Agro-products, Key Laboratory of Biotechnology in Plant Protection of Ministry of Agriculture and Zhejiang Province, Institute of Plant Virology, Ningbo University, Ningbo, China; State Key Laboratory for Managing Biotic and Chemical Threats to the Quality and Safety of Agro-products, Key Laboratory of Biotechnology in Plant Protection of Ministry of Agriculture and Zhejiang Province, Institute of Plant Virology, Ningbo University, Ningbo, China; State Key Laboratory for Managing Biotic and Chemical Threats to the Quality and Safety of Agro-products, Key Laboratory of Biotechnology in Plant Protection of Ministry of Agriculture and Zhejiang Province, Institute of Plant Virology, Ningbo University, Ningbo, China; State Key Laboratory for Managing Biotic and Chemical Threats to the Quality and Safety of Agro-products, Key Laboratory of Biotechnology in Plant Protection of Ministry of Agriculture and Zhejiang Province, Institute of Plant Virology, Ningbo University, Ningbo, China; State Key Laboratory for Managing Biotic and Chemical Threats to the Quality and Safety of Agro-products, Key Laboratory of Biotechnology in Plant Protection of Ministry of Agriculture and Zhejiang Province, Institute of Plant Virology, Ningbo University, Ningbo, China; State Key Laboratory for Managing Biotic and Chemical Threats to the Quality and Safety of Agro-products, Key Laboratory of Biotechnology in Plant Protection of Ministry of Agriculture and Zhejiang Province, Institute of Plant Virology, Ningbo University, Ningbo, China; State Key Laboratory for Managing Biotic and Chemical Threats to the Quality and Safety of Agro-products, Key Laboratory of Biotechnology in Plant Protection of Ministry of Agriculture and Zhejiang Province, Institute of Plant Virology, Ningbo University, Ningbo, China; State Key Laboratory for Managing Biotic and Chemical Threats to the Quality and Safety of Agro-products, Key Laboratory of Biotechnology in Plant Protection of Ministry of Agriculture and Zhejiang Province, Institute of Plant Virology, Ningbo University, Ningbo, China; State Key Laboratory for Managing Biotic and Chemical Threats to the Quality and Safety of Agro-products, Key Laboratory of Biotechnology in Plant Protection of Ministry of Agriculture and Zhejiang Province, Institute of Plant Virology, Ningbo University, Ningbo, China

**Keywords:** horizontal gene transfer, plant defenses, saliva evolution, insect-plant coevolution

## Abstract

Herbivorous insects such as whiteflies, planthoppers, and aphids secrete abundant orphan proteins to facilitate feeding. Yet, how these genes are recruited and evolve to mediate plant–insect interaction remains unknown. In this study, we report a horizontal gene transfer (HGT) event from fungi to an ancestor of Aleyrodidae insects approximately 42 to 190 million years ago. BtFTSP1 is a salivary protein that is secreted into host plants during *Bemisia tabaci* feeding. It targets a defensive ferredoxin 1 in *Nicotiana tabacum* (NtFD1) and disrupts the NtFD1–NtFD1 interaction in plant cytosol, leading to the degradation of NtFD1 in a ubiquitin-dependent manner. Silencing BtFTSP1 has negative effects on *B. tabaci* feeding while overexpressing BtFTSP1 in *N. tabacum* benefits insects and rescues the adverse effect caused by NtFD1 overexpression. The association between BtFTSP1 and NtFD1 is newly evolved after HGT, with the homologous FTSP in its fungal donor failing to interact and destabilize NtFD1. Our study illustrates the important roles of horizontally transferred genes in plant–insect interactions and suggests the potential origin of orphan salivary genes.

## Introduction

Plants and herbivorous arthropods have been locked in a never-ending evolutionary battle, with saliva playing a crucial role in this conflict (Wim et al. 2016; Blaazer et al. 2018; Jiang et al. 2019). Saliva contains a variety of bioactive compounds that enable insects to feed and survive successfully by facilitating stylet penetration and counteracting plant defenses (Miles 1999; van Bel and Will 2016). With the help of high-throughput sequencing, the salivary components of aphids (Carolan et al. 2011; Vandermoten et al. 2014), planthoppers (Huang et al. 2016, 2018), and whiteflies (Huang et al. 2021b) have been uncovered. Interestingly, the saliva of each insect contains a wealth of orphan genes that have no known homologs in other lineages. For example, the LsSP1 gene, which aids planthopper feeding by manipulating plant defenses, is specific to planthoppers and has an unknown origin (Huang et al. 2023). Other critical feeding genes, such as Bsp9 from whiteflies (Wang et al. 2019b), C002 from aphids (Mutti et al. 2008), and SHP from planthoppers (Huang et al. 2015), are only found in specific taxa. Orphan genes are believed to be particularly significant for taxon-specific adaptations and environmental interactions (Long et al. 2003). Nonetheless, the origins and functions of orphan salivary components in herbivore–plant interactions remain largely unknown.

Horizontal gene transfer (HGT), which involves the exchange of genetic material between organisms that are not related by descent, is a significant mechanism by which insects can acquire new genes (Soucy et al. 2015). While the increasing availability of genome sequences has facilitated the discovery and analysis of HGT events, most genes transferred have neutral or slightly detrimental effects, and gradually degenerate or lose their original functions in the recipient (Soucy et al. 2015; Husnik and McCutcheon 2018). However, there are still several well-supported cases in which transferred genes have contributed to the acquisition of novel traits in arthropods, such as male courtship in moths (Li et al. 2022b), essential amino-acid production in mealybugs (Husnik et al. 2013), detoxification of plant produced metabolites in spider mites (Wybouw et al. 2016), and body coloration in aphids (Moran 2010). Despite these exciting discoveries, the extent and functional significance of HGT associated with insect saliva remains unknown. Salivary proteins exhibit a high evolutionary rate (Rao et al. 2019). Although numerous orphan genes have been identified in saliva, none of them have been reported to result from HGT.

Plant ferredoxins (FDs) are small [2Fe–2S] proteins primarily responsible for transporting electrons from photosystem I to various FD-dependent enzymes during photosynthesis (Hanke and Mulo 2013). FDs are initially synthesized in the cytosol, which is later transported into the chloroplast (Arakaki et al. 1997). In recent years, increasing evidence suggested that FDs and FD-like proteins are also involved in plant responses against plant pathogens. For example, overexpression of FD1 in *Nicotiana benthamiana* has been found to confer resistance to Potato virus X infection, while silencing FD1 has the opposite effects (Yang et al. 2020); Similarly, FD-like proteins have been shown to enhance disease resistance against various bacteria (Lin et al. 2010; Ger et al. 2014; Su et al. 2014; Hong et al. 2018). Moreover, FD1 confers plant resistance against rice stripe virus (RSV) (Cui et al. 2021). However, RSV has evolved a virus-derived small interfering RNA (vsiRNA) that downregulates FD1 transcripts, thus suppressing FD1-mediated plant defenses (Cui et al. 2021). To date, the role of plant FDs in coping with insect infestation remains unknown, and no insect component has been reported to mitigate FD-mediated plant immunity. Therefore, further investigation is required to understand the roles of FDs in herbivore–plant interactions.

The whitefly *Bemisia tabaci* (Hemiptera: Aleyrodidae family), is a highly versatile insect that feeds on hundreds of plant species worldwide (Barro et al. 2011). Several researches indicated that the successful feeding and extensive damage of *B. tabaci* is likely enabled by saliva. Our previous work comprehensively analyzed *B. tabaci* salivary proteins by transcriptomic and LC–MS/MS analyses, which revealed the presence of numerous orphan proteins in their salivary secretion (Huang et al. 2021b). However, the functions of most proteins remain unknown. In this study, a fungus-transferred salivary protein BtFTSP1 is employed as a molecular probe to investigate the functions and potential origins of herbivorous saliva. BtFTSP1 is transferred from fungi to a whitefly ancestor after the time Aleyrodidae diverged from Aphididae. It is capable of migrating into plant cells after secretions and improves whitefly performance by inhibiting NtFD1-mediated plant immunity.

## Results

### BtFTSP1 is Horizontally Transferred From Fungi to Aleyrodidae Insects

Our previous study demonstrated that *B. tabaci* saliva contained many orphan proteins that did not have homologs in other insect species (Huang et al. 2021b). To investigate the origin of these genes, homology searches were conducted against viral, bacterial, and fungal databases. One of the orphan genes, BtFTSP1 (fungus transferred salivary protein in *B. tabaci*; Genome accession number, BTA002831; GenBank accession number, QHB15613), showed a high degree of sequence similarity to fungal proteins. For example, BtFTSP1 had 60.2% identity (E-value = 3e-38; Total score = 234) to an FTSP homolog in the fungus *Meira miltonrushii* (MmFTSP-like, XP_025357528). Blasting BtFTSP1 against the *B. tabaci* genome revealed the presence of 3 additional paralogous genes (BtFTSP2, BTA029129, XP_018909720; BtFTSP3, BTA002830, CAH0381103; BtFTSP4, BTA019829, XP_018907192) that showed 35.6% to 51.9% amino-acid sequence similarity to each other (supplementary Fig. S1a and b, Supplementary Material online). The 4 BtFTSPs showed no detectable similarity at the nucleic acid level. The BtFTSP1, BtFTSP2, and BtFTSP3 were located in chromosome 1, while BtFTSP4 was located in chromosome 8 (supplementary Fig. S1c and Table S1, Supplementary Material online). Analyzing the flanking regions of BtFTSPs in the genome demonstrated that genes adjacent to BtFTSPs belonged to insects, further confirming the integration of BtFTSPs into the insect genome (supplementary Table S1, Supplementary Material online). Additionally, there was one intron in BtFTSP4, while no intron was detected in BtFTSP1, BtFTSP2, and BtFTSP3 (supplementary Fig. S1c, Supplementary Material online). All 4 BtFTSPs have a signal peptide at the N-terminus and are closely associated with fungal proteins. However, only BtFTSP1 was detected in the secreted saliva, with 7 unique peptides identified by LC–MS/MS (supplementary Figs. S1d, S2, Supplementary Material online).

**Fig. 1.**
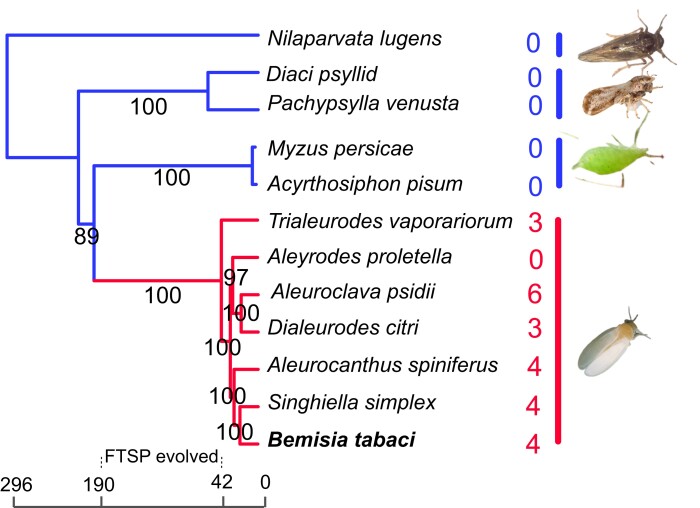
Distribution of FTSPs in Hemipteran insects. The coding regions of *Trialeurodes vaporariorum*, *Aleyrodes proletella*, *Dialeurodes citri*, *Aleuroclava psidii*, *Aleurocanthus spiniferus*, and *Singhiella simplex* were predicted using TransDecoder v5.5.0. The predicated proteins from *Bemisia tabaci*, *Acyrthosiphon pisum*, *Myzus persicae*, *Diaci psyllid*, *Pachypsylla venusta*, and *Nilaparvata lugens* were retrieved from a public database. The phylogenetic tree was constructed based on 18 single copy genes. *N. lugens* was used to root the tree. The divergent time of *D. citri- N. lugens* (177 to 401 Mya) was used for calibration time estimation. The estimated species divergence time is illustrated at the bottom of the phylogenetic tree. The number of FTSP-related contigs for each species is indicated on the right.

Since there are no insect FTSP homologs in the NCBI nr database, we retrieved transcriptomic and genomic data from 21 hemipteran species (supplementary Table S2, Supplementary Material online). Homology searches revealed that FTSP genes were identified in almost all analyzed insects in Aleyrodidae, except for *Aleyrodes proletella*. In contrast, no FTSP homolog was detected in other insects, indicating that FTSP genes are specific to Aleyrodidae (supplementary Table S2, Supplementary Material online). Evolutionary analysis based on 18 single-copy genes demonstrated that Aleyrodidae diverged from Aphididae approximately 42 to 190 million years ago (Mya), which might be the time that FTSP genes were horizontally transferred (Fig. 1). For *A. proletella*, we analyzed 24 high-throughput transcriptomic data being deposited in the SRA database or whole-genome sequencing data generated in this study. However, no FTSP-associated sequence was detected (supplementary Table S2, Supplementary Material online).

**Fig. 2.**
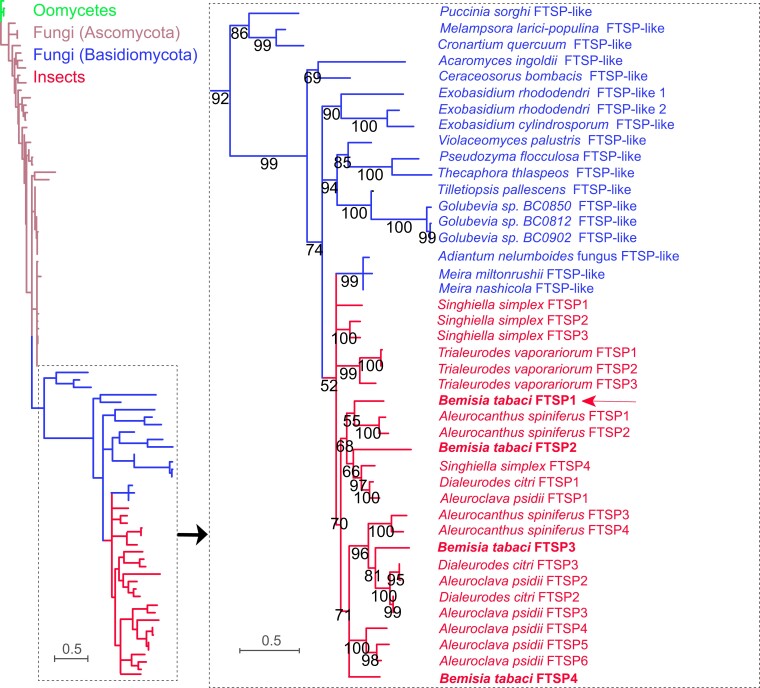
Evolutionary relationship among FTSPs. The evolutionary relationship was constructed using RAxML v0.9.0 based on the maximum likelihood topology with 1,000 bootstrap replicates. Nodes with bootstrap values greater than 50 were displayed. The scale bar represents 0.5 amino-acid substitutions per site. Sequences and the accession numbers are provided in supplementary Table S3 and Data S1, Supplementary Material online. The right image represents the enlarged images of the boxed area on the left. Colors in green, brown, blue, and red represent genes originating from oomycetes, Ascomycota fungi, Basidiomycota fungi, and insects, respectively.

Based on the blast search against the NCBI nr database with a cutoff E-value of 10^−5^, the distribution of FTSP homologs was found to be restricted to insects, fungi, and oomycetes (supplementary Table S3 and Data S1, Supplementary Material online). To learn more about the origin of the FTSP, a maximum likelihood (ML) phylogenetic tree was constructed (Fig. 2). Three oomycete genes, 67 fungal genes, and 24 insect genes were used for analysis (Supplementary Data S1, Supplementary Material online). The results showed that all insect FTSPs were clustered in the same group. Interestingly, *M. miltonrushii*, *M. nashicola*, and an *Adiantum nelumboides*-associated fungus were clustered with insect FTSPs (Fig. 2; supplementary Fig. S3, Supplementary Material online), indicating the potential occurrence of a horizontal gene transfer (HGT) event. Overall, insect FTSPs were more closely associated with homologs from Basidiomycota than from Ascomycota. These results suggest that insect FTSPs are likely transferred from Basidiomycota, potentially from *Meira* species or its ancestor.

### BtFTSP1 is Important for *B. tabaci* Feeding

**Fig. 3.**
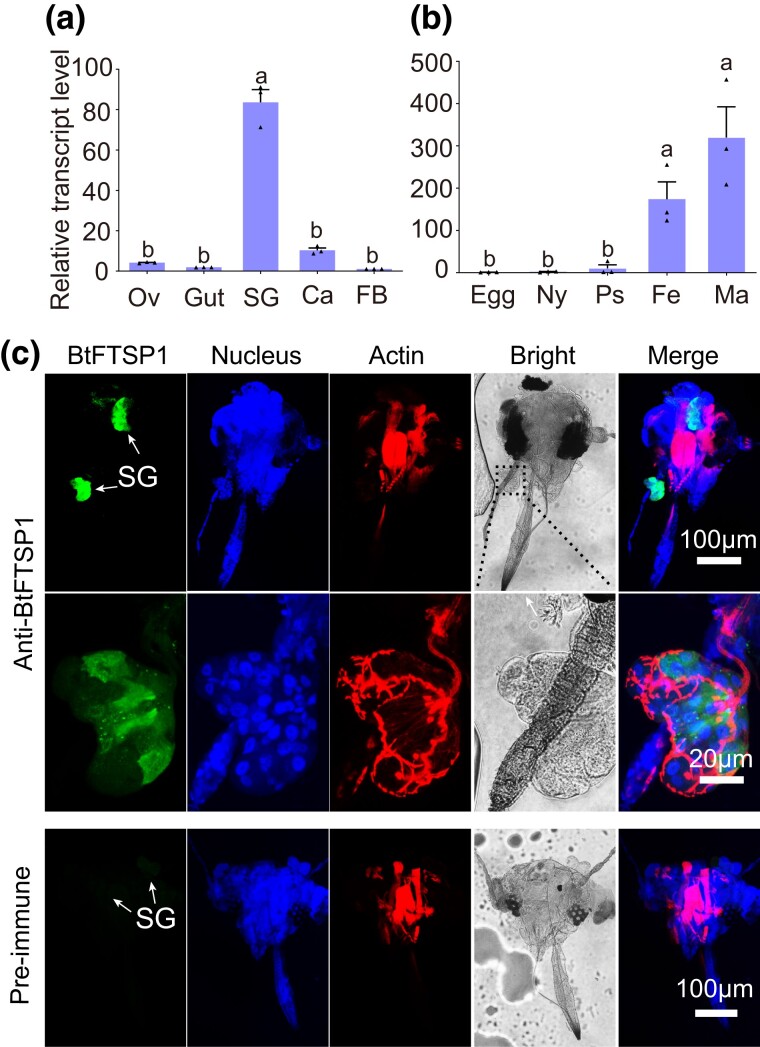
Expression patterns of BtFTSP1. a) qRT-PCR suggested that BtFTSP1 was mainly expressed in the salivary gland. SG, salivary gland; Ca, carcass; FB, fat body; Ov, ovary. b) Expression patterns of BtFTSP1 at different developmental stage. Ny, nymph; Ps, pseudo chrysalis; Fe, female adult; Ma, male adult. c) Immunohistochemical staining of BtFTSP1 in *B. tabaci* head. The insect heads were incubated with anti-BtFTSP1 serum or pre-immune serum conjugated with Alexa Fluor 488 NHS Ester (green) and actin dye phalloidin rhodamine (red) and examined by Leica SP8. The nucleus was stained with DAPI (blue). The lower images represent the enlarged images of the boxed area in the upper image. The boxed area was indicated in a bright filed image.

We chose to analyze the function of BtFTSP1 as it was the only paralog that was validated to be secreted into saliva. The BtFTSP1 transcript was mainly expressed during adult stages, with expression being nearly exclusive to the salivary glands (Fig. 3a and b). Immunohistochemical staining produced similar results, with the BtFTSP1 signal being distributed throughout the salivary glands but not the other tissues (Fig. 3c).

**Fig. 4.**
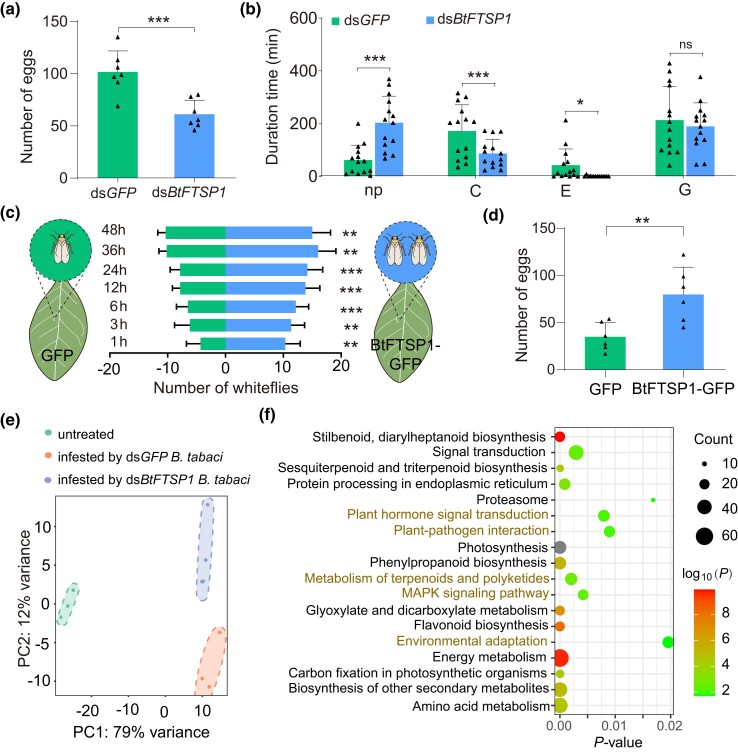
BtFTSP1 enhances *Bemisia tabaci* performance by affecting plant defenses. a) and b) Effect of ds*BtFTSP1* and ds*GFP* treatment on *B. tabaci* fecundity a) and feeding behavior b). Data are present as mean values ± SEM. Insect fecundity is measured by counting the number of deposited eggs. Ten insects, including 5 females and 5 males, were grouped and allowed to oviposit for 3 d. Insect feeding behaviors, including nonpenetration (np), pathway duration (C), phloem sap ingestion (E), and xylem sap ingestion (G), are measured by EPG recordings for 8 h. For fecundity analysis, *n* = 7 independent biological replicates; for EPG analysis, *n* = 14 independent biological replicates. c) Attraction of GFP- and BtFTSP1-GFP-expressed *Nicotiana tabacum* leaves to *B. tabaci* in the 2-choice experiments. *n* = 8 independent biological replicates. d) Number of deposited eggs on GFP- and BtFTSP1-GFP- expressed leaves. *n* = 6 independent biological replicates. *P*-values in (a, b, c, d) were determined by 2-tailed unpaired Student's *t* test. ***P < 0.001; **P < 0.01; *P < 0.05; ns, not significant. e) Principal component analysis (PCA) of gene expression patterns in *N. tabacum* leaves that were untreated or infested by dsRNA-treated *B. tabaci*. The first 2 principal components (PC1 and PC2) based on transcriptomic results are shown. f) Kyoto Encyclopedia of Genes and Genomes (KEGG) pathway enrichment analysis of DEGs between plants infested by ds*GFP*-treated and ds*BtFTSP1*-treated *B. tabaci*. Enriched *P*-values were calculated according to 1-sided hypergeometric test using TBtools software. KEGG pathways that are closely related to plant defenses were labeled in red.

To investigate the function of BtFTSP1, dsRNAs of the green fluorescent protein gene (GFP, served as a negative control) and BtFTSP1 were synthesized and injected into newly emerged adult whiteflies. The interference effect was determined by qRT-PCR, which showed that ds*BtFTSP1* specifically and efficiently suppressed the transcription of the target gene (supplementary Fig. S4a, Supplementary Material online). Silencing BtFTSP1 did not significantly affect the survivorship of adult insects (supplementary Fig. S4b, Supplementary Material online). Scanning electron microscopy (SEM) analysis showed that BtFTSP1 did not contribute to salivary sheath formation, as silencing BtFTSP1 did not influence the structure of the salivary sheath (supplementary Fig. S4c, Supplementary Material online), unlike previously reported gelling proteins (Will and Vilcinskas 2015). However, there was a 40% decrease in fecundity in *B. tabaci* treated with ds*BtFTSP1* compared to the control (Fig. 4a). Electrical penetration graph (EPG) technique, a method of indirectly visualizing the feeding of piercing-sucking insects within plant tissue, was employed to monitor the feeding activity of dsRNA-treated *B. tabaci* (supplementary Fig. S5, Supplementary Material online). The nonpenetration phase (np) was significantly increased in the ds*BtFTSP1* treatment compared to the control (Fig. 4b). In contrast, significant decreases in pathway duration phase (C) and phloem ingestion phase (E) were observed in ds*BtFTSP1*-treated *B. tabaci* (Fig. 4b), indicating impaired feeding processes.

Next, we overexpressed BtFTSP1 with a GFP tag (BtFTSP1-GFP) in *N. tabacum* via *Agrobacterium* infiltration. *N. tabacum* overexpressing BtFTSP1-GFP attracted more *B. tabaci*, resulting in more whiteflies settling and higher fecundity, compared to the GFP overexpression control (Fig. 4c and d). These findings suggest that BtFTSP1 may play a role in plant-host interactions.

To investigate the potential impact of BtFTSP1 on plants, transcriptomic sequencing was performed on *N. tabacum* plants that were untreated, infested with ds*GFP*-treated *B. tabaci*, and infested with ds*BtFTSP1*-treated *B. tabaci*. Principal component analysis (PCA) revealed that the infestation of *B. tabaci* contributed to the majority of variation (79%), while the deficiency of BtFTSP1 secretion accounted for a significant portion of variation (12%) (Fig. 4e). A total of 1,133 differentially expressed genes (DEGs) were identified between *N. tabacum* infested by ds*GFP*-treated *B. tabaci* and ds*BtFTSP1*-treated ones (Supplementary Data S2, Supplementary Material online). Enrichment analysis showed that genes associated with plant hormone signal transduction, plant-pathogen interaction, terpenoids/polyketides metabolism, MAPK signaling, and environmental adaptation were differentially expressed (Fig. 4f). Interestingly, most of the defense-associated genes were induced in *N. tabacum* infested by ds*BtFTSP1*-treated *B. tabaci* (supplementary Data S2, Supplementary Material online), indicating that BtFTSP1 is capable of attenuating plant defenses in host plants.

### BtFTSP1 Interacts With Defensive Ferredoxin (NtFD1) in Tobacco Plants

**Fig. 5.**
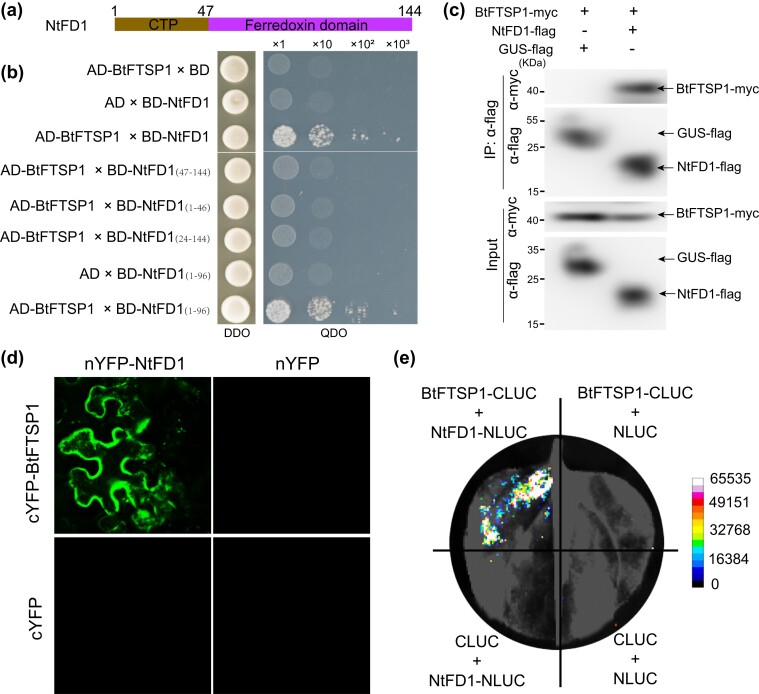
BtFTSP1 interacts with NtFD1. a) Domain organization of NtFD1. NtFD1 contains a chloroplastic transit peptide (cTP) at the N-terminus and a ferredoxin domain at the C-terminus. b) Yeast 2-hybrid assays showing the interaction between BtFTSP1 and different NtFD1 mutants. c) Co-immunoprecipitation (Co-IP) assays showed the interaction between BtFTSP1 and NtFD1. Total proteins were extracted from *Nicotiana benthamiana* leaves expressing BtFTSP1-Myc/NtFD1-Flag and BtFTSP1-Myc/GUS-Flag, then precipitated with Flag beads and probed with anti-Flag and anti-Myc antibodies for immunoblot analysis. d, e) Bimolecular fluorescence complementation (BiFC, d), and luciferase complementation (LUC, e) assays confirmed the interaction between BtFTSP1 and NtFD1.

Yeast 2-hybrid (Y2H) was used to identify the potential interactors of BtFTSP1. BtFTSP1 served as a bait to screen the target genes in a *Nicotiana benthamiana* cDNA library. Five proteins were identified as potential interactors of BtFTSP1, FD1 (ABB30150), cytochrome b6-f complex iron-sulfur (NP_001312705), ribulose bisphosphate carboxylase (XP_016449404), lysine-specific demethylase (XP_016459901), and plasminogen activator inhibitor 1 RNA-binding protein (XP_016458783) (supplementary Table S4, Supplementary Material online). Among them, the FD1 was most highly induced by *B. tabaci* infestation (supplementary Fig. S6, Supplementary Material online). FDs are a group of small [2Fe–2S] cluster proteins that act as electron carriers of low redox potential in chloroplast electron transport chains (Hanke and Mulo 2013). Plant Fds are highly conserved, with a chloroplastic transit peptide (cTP) at the N-terminus and a ferredoxin domain at the C-terminus (Fig. 5a). Recent evidence suggests that FDs and FD-like proteins also play a role in plant responses to biotic stresses (Yang et al. 2020). We validated the interaction between BtFTSP1 and *N. tabacum* FD1 (NtFD1) through point-to-point Y2H, co-immunoprecipitation (Co-IP), bimolecular fluorescence complementation (BiFC), and luciferase complementation (LUC) assays (Fig. 5b-e). Our study showed that neither the cTP nor the ferredoxin domain alone can interact with BtFTSP1 (Fig. 5b). The NtFD1^Δ1–96^ (full cTP region and partial ferredoxin domain), but not NtFD1^Δ24–144^ (partial cTP region and full ferredoxin domain), was responsible for interacting with BtFTSP1 (Fig. 5b).

**Fig. 6.**
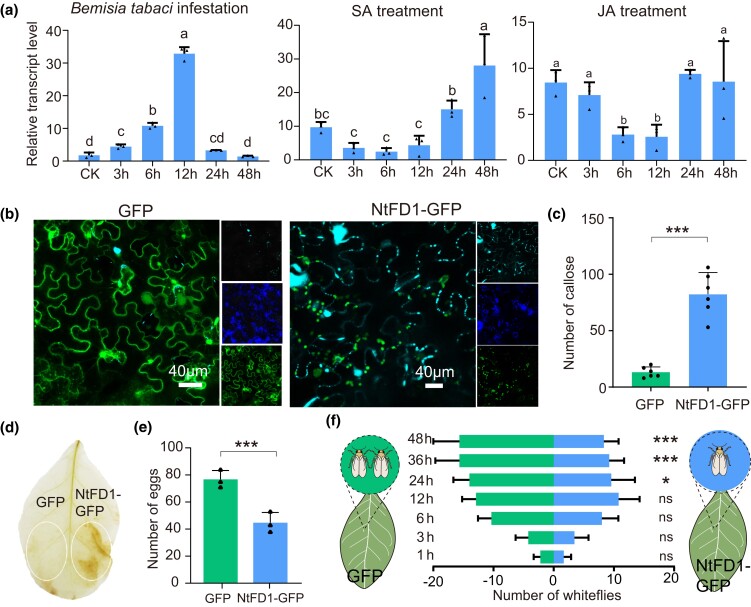
Effects of NtFD1 on plant defenses. a) Expression patterns of NtFD1 in response to *Bemisia tabaci* infestation, salicylic acid (SA) treatment, and jasmonic acid (JA) treatment. Data are presented as mean values ± SEM (*n* = 3 independent biological replicates). Different lowercase letters indicate statistically significant differences at *P* < 0.05 level according to 1-way ANOVA test followed by Tukey's multiple comparisons test. b) Aniline blue staining of GFP and NtFD1-GFP-expressed leaves revealed the callose in plasmodesmata and guard cells. c) The number of callose in plasmodesmata in 400 μm × 400 μm area. d) Accumulation of H_2_O_2_ in *Nicotiana tabacum* revealed by diaminobenzidine (DAB) staining. e) The number of deposited eggs on GFP- and NtFD1-GFP- expressed *N. tabacum* leaves. f) Attraction of GFP- and NtFD1-GFP- expressed leaves to *B. tabaci* in the 2-choice equipment. Data in c, e, and f) are presented as mean values ± SEM. *P*-values were determined by 2-tailed unpaired Student's *t* test. ***P < 0.001; **P < 0.01; *P < 0.05; ns, not significant.

Relative transcript levels of NtFD1 in response to *B. tabaci* infestation were investigated. NtFD1 was significantly induced at 3 h postinfestation, and reached a peak at 12 h (Fig. 6a). Hormonal signals, particularly salicylic acid (SA) and jasmonic acid (JA), play a critical role in plant defense against herbivores (Wang et al. 2019a, Ding and Ding 2020). Our results showed that the expression of NtFD1 was significantly induced after 12 h post-SA treatment, while JA treatment suppressed the NtFD1 expression (Fig. 6a).

Next, NtFD1 with a GFP tag (NtFD1-GFP) was overexpressed in *N. tabacum*. The majority of NtFD1-GFP signal was detected in chloroplast, while a small portion of NtFD1-GFP can be detected in the cytosol (Fig. 6b). Callose deposition and H_2_O_2_ accumulation are markers of plant basal defenses against phloem-sap sucking herbivores (Gao et al. 2022). Our results showed that more callose was deposited in NtFD1-GFP-overexpressing leaves than in the control (Fig. 6b and c). Moreover, NtFD1-GFP overexpression induced significant H_2_O_2_ accumulation (Fig. 6d). The feeding preference of *B. tabaci* on *N. tabacum* plants overexpressing NtFD1-GFP and GFP was compared. The results showed that *N. tabacum* overexpressing NtFD1-GFP was less attractive to *B. tabaci* than the control, with fewer whiteflies settling and lower fecundity (Fig. 6e and f). These results suggest that NtFD1 is involved in plant defense against *B. tabaci*.

### BtFTSP1 Suppresses NtFD1-mediate Plant Defenses

**Fig. 7.**
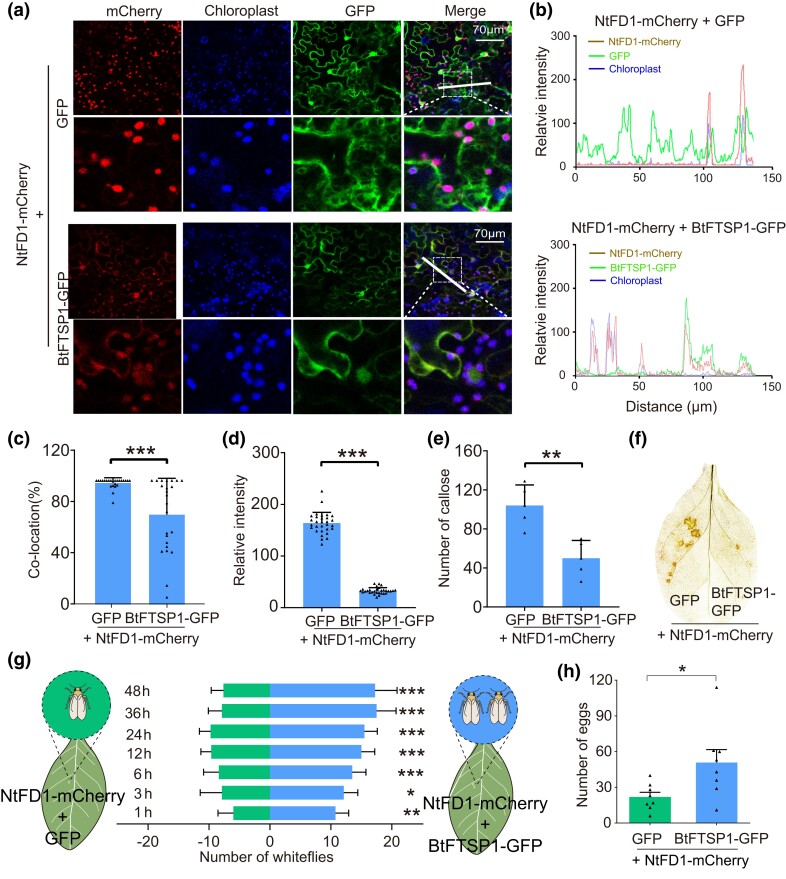
BtFTSP1 suppresses NtFD1-mediate plant defenses. a) BtFTSP1-GFP affected the localization patterns of NtFD1-mCherry in *Nicotiana tabacum*. *N. tabacum* co-expressing GFP and NtFD1-mCherry was used as a control. Chloroplasts (blue) were visualized by autofluorescence. The lower images represent the enlarged images of the boxed area in the upper image. The boxed area was indicated in merge image. The white dashed line indicated the region used for fluorescent analysis in b). Fluorescence was captured under the Leica SP8 confocal microscope. The red-fluorescence of NtFD1-mCherry, which was used to detect the amount of NtFD1-mCherry in NtFD1-mCherry/GFP and NtFD1-mCherry/BtFTSP1-GFP, was captured using the same parameters. b) Analyses of overlapped fluorescence spectra in a). The fluorescent spectra were calculated using Leica Application Suite X software. Fluorescence signals were obtained from the white dashed line. c) Colocation of NtFD1-mCherry and chloroplast. The portion of NtFD1-mCherry that colocalized with chloroplast was analyzed. d) Relative intensity of NtFD1-mCherry was compared between GFP and BtFTSP1-GFP treatments. The fluorescent intensity was calculated using Leica Application Suite X software. e) The number of callose in plasmodesmata in 400 μm × 400 μm area. f) Accumulation of H_2_O_2_ in *N. tabacum* revealed by diaminobenzidine (DAB) staining. g) Attraction of *B. tabaci* to *N. tabacum* leaves that expressed with NtFD1-mCherry/GFP and NtFD1-mCherry/BtFTSP1-GFP. h) The number of deposited eggs on indicated *N. tabacum* leaves. Data in c, d, e, g, and h) are presented as mean values ± SEM. *P*-values were determined by 2-tailed unpaired Student's *t* test. ***P < 0.001; **P < 0.01; *P < 0.05; ns, not significant.

To investigate the effect of BtFTSP1 on NtFD1, NtFD1 with a mCherry tag (NtFD1-mCherry) were co-expressed with BtFTSP1-GFP in *N. tabacum*. As a result, the localization patterns of NtFD1-mCherry changed in the presence of BtFTSP1-GFP (Fig. 7a). The NtFD1-mCherry and BtFTSP1-GFP overlapped, and more NtFD1-mCherry retained in cytosol, whereas NtFD1-mCherry and GFP did not overlap, with most NtFD1-mCherry located in chloroplast (Fig. 7b and c). The NtFD1-mCherry signal was attenuated in the presence of BtFTSP1-GFP when compared to the GFP control (Fig. 7d).

BtFTSP1 significantly suppressed the callose deposition and H_2_O_2_ accumulation triggered by NtFD1 overexpression (Fig. 7e and f). Moreover, feeding preference analysis showed that BtFTSP1 can rescue the plant defense caused by NtFD1 overexpression, as more whiteflies settled and oviposited on *N. tabacum* plants overexpressing NtFD1 and BtFTSP1 than on the control (Fig. 7g and h). These results suggest that BtFTSP1 can suppress NtFD1-mediated plant defenses.

### BtFTSP1 Destabilizes NtFD1 by Disassociating the NtFD1 Polymer

**Fig. 8.**
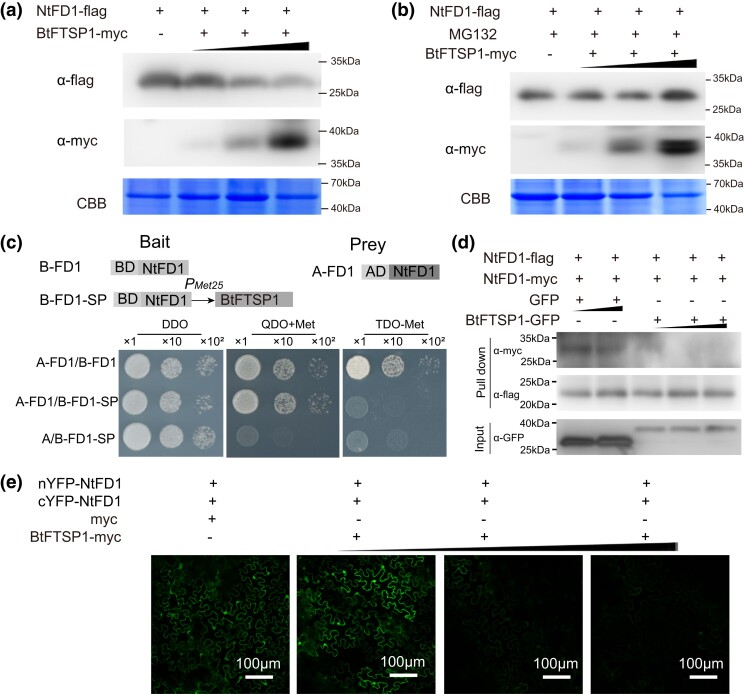
BtFTSP1 destabilizes NtFD1 by disassociating NtFD1–NtFD1 interaction. a) BtFTSP1 promotes NtFD1 degradation *in planta*. NtFD1-Flag was co-expressed with different concentration of BtFTSP1-Myc in *Nicotiana benthamiana*. b) BtFTSP1 failed to destabilize NtFD1 in the presence of MG132. The 26S proteasome inhibitor MG132 was used to inhibit the ubiquitin-dependent protein degradation. The leaves were infiltrated with 50μM MG132 at 24 h post *Agrobacterium tumefaciens* infiltration. The protein level of NtFD1-Flag and BtFTSP1-Myc were quantified by western-blotting. Coomassie brilliant blue (CBB) staining was conducted to visualize the amount of sample loading. c to e) BtFTSP1 disassociates the NtFD1–NtFD1 interaction in Y3H c), competitive pull-down d), and competitive BIFC e) assays. c) In BK-NtFD1-BtFTSP1 (B-FD1-SP), the expression of BtFTSP1 was inhibited in the presence of methionine and induced by its absence. The recombinant pBridge vectors, together with the recombinant pGADT7-NtFD1 vector were transfected into the yeast, and incubated on the DDO (SD/−Leu/–Trp), QDO (SD/−Ade/−His/−Leu/−Try) and TDO (SD/−Leu/–Trp/–Met) medium, respectively. d) In dose-dependent pull-down assay, the same amount of NtFD1-Flag and NtFD1-Myc were mixed with different amounts of BtFTSP1-GFP and GFP, respectively. The amounts of NtFD1-Myc bound to NtFD1-Flag decreased in the presence of BtFTSP1-GFP. e) In competitive BIFC assay, the cYFP-NtFD1 and nYFP-NtFD1 were mixed with different concentration of BtFTSP1-Myc. MG132 was used to inhibit ubiquitin-dependent protein degradation.

According to the results in Fig. 7, we hypothesized that BtFTSP1 may be able to degrade NtFD1. To test this hypothesis, we co-expressed NtFD1-Flag with different concentrations of BtFTSP1-Myc. The protein level of NtFD1-Flag decreased significantly with increasing amounts of BtFTSP1-Myc, while the transcript level of NtFD1-Flag remained unchanged (Fig. 8a; supplementary Fig. S7, Supplementary Material online). This result indicates that the decrease in protein level is not due to changes in transcription but rather due to degradation. To confirm this, we used MG132, a 26S proteasome inhibitor, which reduces the degradation of ubiquitin-conjugated proteins in eukaryotes (Li et al. 2022a). We found that the BtFTSP1-Myc failed to degrade NtFD1-Flag in the presence of MG132 (Fig. 8b), suggesting that BtFTSP1 may play a role in the degradation of NtFD1 in a ubiquitin-dependent manner.

We performed Y2H to investigate the tobacco proteins interacting with NtFD1 and found that NtFD1 potentially interacted with one another (supplementary Fig. S8, Supplementary Material online). This interaction was further confirmed by point-to-point Y2H, Co-IP, and BiFC assays (supplementary Fig. S8a-d, Supplementary Material online). Then, we subjected the NtFD1-Flag proteins to native PAGE assays, which demonstrated that NtFD1 formed polymers, mainly the tetramer (supplementary Fig. S8e, Supplementary Material online). Additionally, mutation assays further confirmed that NtFD1^Δ1–96^ is critical for NtFD1- NtFD1 interaction (supplementary Fig. S8a, Supplementary Material online).

As the NtFD1^Δ1–96^ is also essential for its interactions with BtFTSP1, we speculated that BtFTSP1 might compete with NtFD1–NtFD1 interaction. To test this hypothesis, we used yeast 3-hybrid (Y3H) assays with NtFD1 as the bait and drove the expression of BtFTSP1 using the methionine-inducible Met25 promoter (Fig. 8c). In this assay, the expression of BtFTSP1 was inhibited in the presence of methionine and induced by its absence. The results showed that NtFD1 interacted with NtFD1 in the absence of BtFTSP1, while NtFD1- NtFD1 interaction was completely abolished in the presence of BtFTSP1 (Fig. 8c). Furthermore, dose-dependent pull-down assays were used to assess whether BtFTSP1 interfered with the NtFD1–NtFD1 interaction in vitro. We mixed the same amount of NtFD1-Flag and NtFD1-Myc with different amounts of BtFTSP1-GFP and immobilized them onto anti-Flag beads. The results showed that the amounts of NtFD1-Myc bound to NtFD1-Flag decreased in the presence of BtFTSP1-GFP (Fig. 8d), indicating that BtFTSP1 disrupted NtFD1–NtFD1 interaction. Additionally, competitive BiFC assays were carried out leaves of *N. benthamiana*, with cYFP-NtFD1 and nYFP-NtFD1 mixed with different concentrations of BtFTSP1-Myc. As BtFTSP1 destabilizes NtFD1 in a ubiquitin-dependent manner, all leaves were infiltered with MG132 to inhibit NtFD1 degradation. We found that the YFP fluorescence signal attenuated with the increasing amounts of BtFTSP1-Myc (Fig. 8e). These results suggest that BtFTSP1 potentially disassociates the NtFD1–NtFD1 interaction by competitive binding.

### NtFD1 Destabilization is a Newly Evolved Function of BtFTSP1, Which is not Found in Its Fungi Donor

Aleyrodidae FTSPs exhibited high sequence similarity with MmFTSP-like in fungi (supplementary Fig. S3, Supplementary Material online). The MmFTSP-like is a secretory protein that contains a signal peptide at its N-terminus. To investigate whether MmFTSP-like functions similarly to BtFTSP1 in manipulating NtFD1, the interaction between MmFTSP-like and NtFD1 was investigated. The result revealed that MmFTSP-like failed to interact with NtFD1 (supplementary Fig. S9a, Supplementary Material online). Additionally, MmFTSP-like was unable to destabilize NtFD1, which was significantly different from the BtFTSP1 (supplementary Fig. S9b, Supplementary Material online). Therefore, the underlying mechanism of functional differences between MmFTSP-like and BtFTSP1 deserves further investigation.

## Discussion

Herbivorous insects have complex relationships with their host plants and other organisms. A thorough understanding of these interactions can provide significant insights into the evolution of life. In this study, we investigated the origin and function of BtFTSPs. We found that these genes were potentially acquired through HGT from fungi to the whitefly ancestor. Analyzing different versions of *B. tabaci* genomes, we confirmed that BtFTSPs were integrated into the insect genome, with their flanking regions belonging to insects. The HGT event of FTSPs has provided whiteflies with the ability to mitigate plant immunity, thereby enhancing their feeding behavior. Our results suggest that HGT may represent an important origin of salivary effectors, with significant implications for shaping the interactions between herbivores and their host plants.

Our phylogenetic analysis revealed that insect FTSPs are orphan genes that are unique to Aleyrodidae species (Fig. 1). We traced their origin back to HGT events from the ancestor of Basidiomycota fungi, with a more speculative possibility of being transferred from *Meira* species (Fig. 2). The genus *Meira* was originally discovered in mite cadavers from citrus leaves and fruit (Boekhout et al. 2003), and has since been found to be an endophyte in several plants (Yasuda et al. 2006; Paz et al. 2007; Vega et al. 2010). It is possible that the ancestors of whitefly and *Meira* shared the same plants or the ancestor of *Meira* infected whitefly, leading to an accidental exchange of genetic material. We analyzed 7 Aleyrodidae species and found that 6 contained 3 to 6 FTSP paralogues, except for *A. proletella* (supplementary Table S2, Supplementary Material online). No FTSP-associated sequence was detected in high-throughput transcriptomic or genomic data of this species. Based on the phylogenetic tree of Aleyrodidae species, *A. proletella* likely evolved after *T. vaporariorum*, a species with 3 TvFTSP paralogues (Fig. 1). As all TvFTSPs were tandemly arrayed in the same scaffold in the genome and showed high sequence similarity (supplementary Fig. S10, Supplementary Material online), FTSPs in *T. vaporariorum* might be acquired through one HGT event and evolved via autochthonous duplication. It is likely that one HGT event occurred at the early stage of Aleyrodidae evolution, and the transferred FTSP was lost in *A. proletella.* However, given the low bootstrap support for the outgroup placement of *T. vaporariorum* (Fig. 1), we cannot exclude the possibility that *A. proletella* is the earliest diverging member of that clade. If so, FTSPs were potentially transferred after divergency of *A. proletella*, and no HGT event occurred in this species. The phylogeny of Aleyrodidae species deserves further investigation. For *B. tabaci*, we identified 4 BtFTSP1 paralogs that clustered in different subgroups. It remains unclear how many HGT events occurred in *B. tabaci*, as recent studies have shown that many apparent gene duplications are actually the result of HGT instead of autochthonous gene duplication (Treangen and Rocha 2011; Soucy et al. 2015).

FD1 confers plant resistance against both plant pathogens (Ma et al. 2020; Cui et al. 2021) and herbivorous insects (Fig. 6b to f). Its transcript levels were found to increase upon *B. tabaci* infestation and SA treatment (Fig. 6a). The interplay between the SA-JA signaling pathways is believed to allow plants to select an effective defense mechanism based on the type of invader they are confronting (Koornneef and Pieterse 2008). Previous research has shown that *B. tabaci* activates the SA signaling pathway, hindering the effectiveness of JA defenses (Xu et al. 2019). However, SA is also crucial for plant defense against herbivores (Ding and Ding 2020). It has been demonstrated that FDs positively regulate the accumulation of SA in several plants (Wang et al. 2018; Yang et al. 2020; Fonseca et al. 2020 ), while SA is capable of inducing callose deposition (Yang et al. 2020), terpenoids biosynthesis (Soltani et al. 2020), and H_2_O_2_ accumulation (Molinari 2007 ), which restrict insect prolonged feeding. Therefore, *B. tabaci* potentially needs to employ other means to dampen SA-mediated defense responses, including the upregulation of NtFD1. Our study indicates that BtFTSP1 can inhibit NtFD1-mediated plant immunity, suggesting that while SA levels increase during infestation, SA-mediated plant defenses may be redirected through other pathways.

It has been reported that the virus secretes vsiRNA to suppress FD1-mediated plant immunity (Cui et al. 2021). However, it is unknown whether insects have evolved a strategy similar to that of the virus. Our study suggests that the horizontally transferred BtFTSP1 interacts with NtFD1 and destabilizes it in a ubiquitin-dependent manner by disassociating the NtFD1–NtFD1 interaction (Fig. 8). This indicates that different organisms have independently evolved effectors to target defensive FD1 components, thus facilitating colonization. Noteworthily, the capacity of NtFD1 destabilization is not found in FTSP homologs in its fungi donor (supplementary Fig. S9, Supplementary Material online). It remains unclear how BtFTSP1 gained this new function in long-term evolution. For many reported HGT events, similar functions are found in both the donor and recipient organisms (Pauchet and Heckel 2013; Husnik and McCutcheon 2018; Ren et al. 2020). In addition to destabilizing defensive NtFD1, BtFTSP1 potentially exerts other functions during insect feeding, with some being conserved with its fungi donor, which deserves further investigation.

**Fig. 9.**
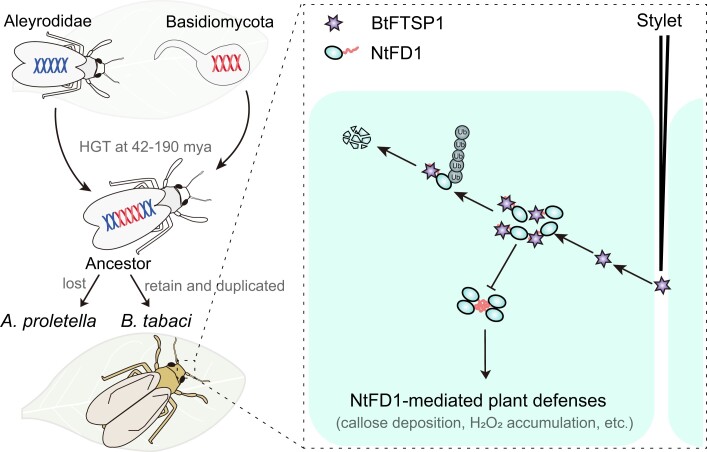
Proposed model for the suppression of NtFD1-mediated plant defenses by horizontally transferred BtFTSP1. The FTSP gene was horizontally transferred from plant-associated Basidiomycota fungi to ancestors of herbivorous Aleyrodidae insects approximately 42 to 190 million years ago (mya). The horizontally transferred FTSP gene was potentially lost in *Aleyrodes proletella*. However, the BtFTSP1 in *Bemisia tabaci* was domesticated as a salivary protein that can be secreted during insect feeding and migrated into plant cells. It is capable of targeting defensive NtFD1 and disrupts the NtFD1–NtFD1 interaction, leading to the ubiquitin-dependent degradation of NtFD1. NtFD1 is a defensive protein that induces callose deposition and H_2_O_2_ accumulation, possibly through the regulation of salicylic acid (Wang et al. 2018; Yang et al. 2020; Fonseca et al. 2020). The decreased NtFD1 level in the host plant is beneficial to insect feeding.

Based on our results, we propose a picture of FTSP evolution and function (Fig. 9). It is likely that the ancestors of Aleyrodidae insects and Basidiomycota fungi shared a common host plant or that the fungi infected insects, resulting in an accidental transfer of genetic material approximately 42 to 190 mya. In *A. proletella*, the horizontally transferred FTSP gene was potentially lost, while in *B. tabaci*, the BtFTSP1 was domesticated as a salivary protein that can be secreted during insect feeding and migrated into plant cells. In long-term evolution, the BtFTSP1 evolved a novel function of suppressing NtFD1-mediated plant defenses, which was not found in its fungi donor. NtFD1 is a defensive protein that induces callose deposition and H_2_O_2_ accumulation, possibly through the regulation of salicylic acid (Wang et al. 2018; Yang et al. 2020; Fonseca et al. 2020). The defensive NtFD1 form polymer, likely the tetramer, to avoid the degradation. The salivary BtFTSP1 competes with NtFD1 for binding sites and disrupts the NtFD1–NtFD1 interaction, leading to the ubiquitin-dependent degradation of NtFD1. This process results in a decrease in the level of NtFD1, which is advantageous for *B. tabaci* feeding.

## Materials and Methods

### Insects and Plants

The *B. tabaci* culture of cryptic species MED was originally collected from soybean plants in Suzhou, An’hui Province, China. The insects were maintained in laboratory conditions on *N. tabacum* plants at 25 ± 1 °C, 50% to 70% relative humidity, and 16 h light/8 h darkness. In addition, *N. benthamiana* and *N. tabacum* plants were kept in a growth chamber at 23 ± 1 °C under a light/dark photoperiod of 16 h/8 h.

### Identification of Horizontal Transferred Salivary Proteins

Our previous work identified 171 salivary proteins in *B. tabaci* saliva (Huang et al. 2021b). To identify the potential origin of these proteins, the Aleyrodidae-restricted genes were first identified by blast search against the predicted proteins in *Acyrthosiphon pisum* (Li et al. 2019), *Nilaparvata lugen*s (Xue et al. 2015), *Riptortus pedestris* (Huang et al. 2021a), *Diaci psyllid* (Carlson et al. 2022), and *Drosophila melanogaste*r (Hoskins et al. 2007) with a cutoff E-value of 10^−10^, respectively. Genes with no homology in the above species were subsequently searched against the viral, bacterial, and fungal databases with a cutoff E-value of 10^−10^. As a result, BtFTSP1 was found to be potentially transferred from fungi. The BtFTSP1 were further blast against the scaffold-level *B. tabaci* genome (Xie et al. 2017) and chromosome-level *B. tabaci* genome (https://www.ncbi.nlm.nih.gov/datasets/genome/GCA_918797505.1/, access on 2023 September 1) to identify the potential paralogous genes with a cutoff E-value of 10^−10^. Additional 3 paralogous genes (BtFTSP2, BtFTSP3, and BtFTSP4) were identified. The presence of these 4 BtFTSPs in *B. tabaci* saliva was examined according to LC-MS/MS data previously reported (Huang et al. 2021b).

### Identification of FTSP Homologues in Insects and Fungi

No FTSP homologs from other insects were identified using the regular blast search against the NCBI nr database. To learn more the FTSP phylogeny, high-throughput raw data of *A. proletella* (SRX14998513-SRX14998536), *Dialeurodes citri* (SRR2980521), *Aleurocanthus spiniferus* (SRR17330024), *Aleuroclava psidii* (SRR16114381), and *Singhiella simplex* (ERR3781281) were retrieved from NCBI SRA database. The raw data were de novo assembled using SPAdes (v3.13.0) with default parameters. Additionally, the assembled genome from 15 hemipteran species was downloaded from NCBI (supplementary Table S2, Supplementary Material online). The assembled contigs or genome were searched against the BtFTSPs to identify the potential homologs in other insects using BLASTX with a cutoff E-value of 10^−10^. The FTSP homologs in fungi were identified using Position-Specific Iterated BLAST (https://blast.ncbi.nlm.nih.gov/Blast.cgi) with a cutoff E-value of 10^−5^. Noteworthily, one FTSP homologs (AnFTSP-like, MCO5562931.1) in NCBI nr database is annotated as a hypothetical protein from the ferns, *Adiantum nelumboides*. It originated from whole genome shotgun sequencing using PacBio Sequel, and assembled by Nextdenovo v. v2.3.0 (Zhong et al. 2022). AnFTSP-like is located in scaffold JAKNSL020002928. However, all the other genes in this scaffold belonged to fungi. Therefore, AnFTSP-like might originated from a fungus that infects ferns, and we annotated it as *Adiantum nelumboides* fungus FTSP-like.

### Whole-Genome Sequencing of *A. proletella*

No FTSP-associated transcript was detected in high-throughput transcriptomic data of *A. proletella* (SRX14998513-SRX14998536). Therefore, whole-genome sequencing was performed on this species to identify potential FTSP-associated sequences in the genome. The *A. proletella* specimen used in this study was kindly provided by De-Ying Ma (Xinjiang Agricultural University, China). Genomic DNA was extracted from a single adult *A. proletella* using the DNeasy Blood & Tissue Extraction Kit (Qiagen Inc.). After the determination of the DNA quality and quantity, a paired-end sequencing library (350 bp in length) was constructed and sequenced using the Illumina NovaSeq6000. The output data were de novo assembled using Trinity v2.8.5 with default parameters. COI was employed to confirm that the species under investigation is *A. proletella*.

### Phylogenetic Analysis of FTSPs

The FTSP homologs from insects, fungi, and oomycetes were used to construct a phylogenetic tree. Briefly, the amino-acid sequences were aligned with MAFFT (v7.310) with default parameters (maxiterate:1000), and ambiguously aligned regions were trimmed by Gblock (Katoh and Standley 2013). Then, the best-fit model of amino-acid substitution (PROTGAMMAJTT) was evaluated by ModelTest-NG v0.1.6. The maximum likelihood (ML) trees were constructed using RAxML v0.9.0 with 1,000 bootstrap replications (Kozlov et al. 2019). Details of all the reference sequences used in phylogenetic analysis are listed in supplementary Table S3 and Data S1, Supplementary Material online.

### Phylogenetic Analysis of Insects

To learn the phylogenetic relationship of insects, the coding regions of assembled contigs from 6 Aleyrodidae species were first predicted using TransDecoder (https://anaconda.org/bioconda/transdecoder). The predicted proteins from *B. tabaci*, *T. vaporariorum*, *A. pisum*, *Myzus persicae*, *D. psyllid*, *Pachypsylla venusta*, and *N. lugens* were retrieved from a public database. All-versus-all BLASTp algorithm was used to find the homologous pairs of sequences with a significant cutoff E-value of 10^−5^. Thereafter, the BLASTp result was converted into a normalized similarity matrix and processed using OrthoMCL v2.0.9 with default parameters (Li et al. 2003). Additionally, protein families were identified by Markov chain clustering MCL-14 to 137 (Enright et al. 2002). The phylogenetic tree was then constructed using single-copy orthologues in each species (1:1:1 gene identified by OrthoMCL analysis), and *N. lugens* was utilized to root the tree. Moreover, sequence alignment was performed by MAFFT. Conserved amino-acid sites were identified by TrimAl v1.2 (Capella-Gutiérrez et al. 2009). Furthermore, ModelTest-NG was employed to determine the best model (PROTGAMMAJTT). Then, a ML tree was constructed using RAxML under the LG + I + G4 + F model with 1,000 bootstraps replications. Later, the divergence time was estimated by r8s v1.81 with known time divergence data in TIMETREE (www.timetree.org) (Sanderson 2003). At last, the divergent time of *D. citri- N. lugens* (177 to 401 Mya) was used for calibration time estimation.

### RNA Interference

The DNA sequences of target genes were amplified using the primers listed in supplementary material S5, Supplementary Material online, and cloned into pClone007 Vector (#TSV-007, Tsingke, Beijing, China). Double-stranded RNAs were synthesized from PCR-generated DNA templates containing the T7 sequence using a T7 High Yield RNA Transcription Kit (#TR101-01, Vazyme). RNA interference experiment was conducted as previously described (Xu et al. 2015). Briefly, newly emerged whiteflies were anesthetized with carbon dioxide for 5 to 10 s, and then dsRNA was injected into the insect mesothorax using a FemtoJet (Eppendorf-Netheler-Hinz, Hamburg, Germany). The insects were kept on *N. tabacum* leaves and living ones were selected for further investigation. The silencing efficiency was determined on 4th day post-injection using the qRT-PCR method.

### Insect Bioassays

To analyze survivorship, newly emerged *B. tabaci* were treated with dsRNA and kept on *N. tabacum* leaves, and their mortality rates were recorded for 10 consecutive days. Three independent replications were conducted. To analyze fecundity, newly emerged adults were treated with dsRNA. Ten insects, including 5 females and 5 males were grouped and placed onto *N. tabacum* leaves one day later. The insects were allowed to oviposit for 3 d. The number of eggs (deposited by 5 females in each replicate) was counted, and at least 6 replicates were conducted for each treatment. For host choice analysis, *N. tabacum* leaves were detached and wrapped with moist cotton at the petiole. Two leaves were placed in a plastic disc (diameter: 50 cm) with a release chamber in the middle. A group of 30 whiteflies were then released into the chamber, and the number of insects settling on each leaf was counted at 1, 3, 6, 12, 24, 36, and 48 h. At least 8 replicates were performed for this analysis.

### EPG Recording Analysis

The GiGA-8d EPG amplifier (Wageningen Agricultural University, Wageningen, The Netherlands) was used to record EPG data. This amplifier has a 10 TΩ input resistance and an input bias current of less than 1 pA. The dsRNA-treated *B. tabaci* were starved on filter paper with only water provided for 12 h before being anesthetized with CO_2_ for 10 s. A gold wire (Wageningen Agricultural University, diameter: 20 mm; length: 5 cm) was then connected to the insect abdomen and the EPG amplifier using water-soluble silver conductive glue (Wageningen Agricultural University). For the plant electrode, a copper wire (diameter: 2 mm; length: 10 cm) was inserted into soil planted with one rice plant. EPG recording was carried out for 8 h in a Faraday cage (120cm × 75cm × 67 cm, Dianjiang, Shanghai, China) with a gain of 50× and output voltage adjusted between −5 V and +5 V.

The recorded data were analyzed using PROBE 3.4 (Wageningen Agricultural University), and the insect feeding behaviors were classified into 4 categories: nonpenetration (np), pathway duration (C), phloem-sap ingestion (E), and xylem sap ingestion (G) (Civolani et al. 2014). At least 10 replicates were performed for each treatment.

### Scanning Electron Microscopy (SEM)

*B. tabaci* were allowed to feed on artificial diets for 24 h. The parafilm attached to the salivary sheath was cut and washed with 1×PBS. Later, SEM samples were attached to a stub and dried in a desiccator under vacuum. After gold-sputtering, the samples were observed by SEM TM4000 II plus (Hitachi, Tokyo, Japan).

### Agrobacterium-mediated Plant Transformation in *N. tabacum* and *N. benthamiana*

The recombinant expression vectors were transfected into *A. tumefaciens* strain GV3101 by the heat transfer method and were grown on LB medium containing 50 μg/mL kanamycin and 10 μg/mL rifampicin for approximately 60 h at 28 °C. Later, the colonies containing target vectors were further amplified in LB medium and collected by centrifugation at 2,400 × *g* for 2 min. Subsequently, the agrobacterium was suspended in an induction buffer (10 mM MgCl_2_, 10 mM MES (pH 5.6), 200 μM Acetosyringone) at indicated OD_600_. In detail, OD_600_ was set to 1.0 for regular experiments. For degradation or competitive BiFC assays, the OD_600_ was set to 0.1, 0.5, and 1.0. After mixing equal amounts of the selected combinations, the suspension was infiltrated into the *N. tabacum* and *N. benthamiana* leaves.

### Transcriptomic Sequencing and Data Analysis

Samples of untreated *N. tabacum* plants and *N. tabacum* plants infested by ds*GFP*- and ds*BtFTSP1*-treated *B. tabaci* for 24 h were collected and homogenized in TRIzol Reagent (#10296018, Invitrogen, Carlsbad, CA, USA). Total RNA was then extracted from the homogenized samples following the manufacturer's instructions, and the RNA samples were sent to Novogene Institute (Novogene, Beijing, China) for transcriptomic sequencing as previously described (Huang et al. 2021b). Poly (A)+ RNA was purified from 20 μg pooled total RNA using oligo (dT) magnetic beads. Fragmentation was performed at 94 °C for 5 min in the presence of divalent cations. Reverse transcription was carried out using N6 random primers to create double-stranded cDNA. The cDNA was end-repaired, adaptor-ligated, and PCR-amplified to create a cDNA library, which was purified using a QIAquick PCR purification kit (Qiagen, Hilden, Germany). The library was sequenced on an Illumina NovaSeq 6000 platform, and all sequencing data were submitted to the NCBI Sequence Read Archive under accession number PRJNA947676.

The internal software was used to filter the output raw reads, and the clean reads from each cDNA library were aligned to the reference *N. tabacum* genome. Low-quality alignments were filtered using SAMtools v1.7 (Li 2009). Cufflink v2.2.1 was used to calculate transcripts per million (TPM) expression values (Trapnell et al. 2012). DESeq2 v2.2.1 was used to analyze the DEGs, and genes with log2-ratio > 1 and adjusted *P*-value < 0.05 were identified (Wang et al. 2010). To reveal overall differences in gene expression patterns among different transcriptomes, PCA analysis was performed using R function plotPCA (github.com/franco-ye/TestRepository/blob/main/PCA_by_deseq2.R), and correlation analysis was carried out using DNAstar v8.0 (Clewley 1995). TBtools software v1.0697 was used to perform KEGG enrichment analyses (Chen et al. 2020). Enriched *P*-values were calculated using a 1-sided hypergeometric test: $P=1-\sum_{i=0}^{m-1}\;\left(\left(\begin{array}{c} M\\ i \end{array}\right)\left(\begin{array}{c} N-M\\ n-i \end{array}\right)\;/\left(\begin{array}{c} N\\ n \end{array}\right)\right)$, where *N* represents the number of genes with KEGG annotation, *n* represents the number of DEGs in *N*, *M* represents the number of genes in each KEGG term, and *m* represents the number of DEGs in each KEGG term.

### *B. tabaci* Infestation and Hormonal Treatment

The 4 to 5-week *N. tabacum* leaves were infested by newly emerged *B. tabaci*. Similarly, the leaves were sprayed with 500 μM SA (#84210, Sigma-Aldrich, St. Louis, MO, USA) and 100 μM JA (Sigma-Aldrich). The treated plants were maintained in a climate chamber at 25 °C, and samples were collected at indicated time points.

### Quantitative Real-Time PCR (qRT-PCR) Analysis

Female *B. tabaci* were dissected to obtain different tissue samples, including carcasses (10), fat bodies (10), guts (20), salivary glands (40), and ovaries (10), using a pair of forceps from each sample. In addition, various developmental stages of *B. tabaci*, including eggs (50), nymphs (20), pupae (20), adult males (20), and females (20) were collected. The number of insects in each sample is indicated in parentheses. To extract RNA from *N. tabacum*, plant samples were ground with liquid nitrogen, and then homogenized using the TRIzol Total RNA Isolation Kit (#9109, Takara, Dalian, China) following the manufacturer's instructions. The first strand of cDNA was synthesized from RNA using HiScript II Q RT SuperMix (#R212-01, Vazyme, Nanjing, China). qRT-PCR was performed on a Roche Light Cycler 480 Real-Time PCR System (Roche Diagnostics, Mannheim, Germany) using the SYBR Green Supermix Kit (#11202ES08, Yeasen, Shanghai, China). The PCR conditions were as follows: initial denaturation at 95 °C for 5 min, followed by 40 cycles of denaturation at 95 °C for 10 s, and annealing at 60 °C for 30 s. The primers used in qRT-PCR were designed using Primer Premier v6.0 (supplementary Table S5, Supplementary Material online). *B. tabaci* actin and *N. tabacum* tubulin were used as internal controls, respectively. The relative quantitative method (2^−ΔΔCt^) was used to evaluate the quantitative variation. Any qRT-PCR results with a Ct value ≥35 were considered as a lack of gene expression in the sample. Three independent biological replicates, each repeated twice, were performed.

### Immunohistochemistry (IHC) Staining

The *B. tabaci* heads with salivary glands attached were carefully dissected and fixed in 4% paraformaldehyde (#E672002, Sango Biotechnology, Shanghai, China) for 30 min. The custom service of Huaan Biotechnology Company (Hangzhou, China) was used to produce the anti-BtFTSP1 serum by immunizing rabbits with purified GST-BtFTSP1 proteins. The GST-BtFTSP1 was expressed in *E. coli* strain Transetta (TransGen Biotech, Beijing, China) and purified using glutathione-sepharose beads (#C600031-0005, Sango Biotechnology). To enable visualization, the anti-BtFTSP1 serum was conjugated with Alexa Fluor 488 NHS Ester (#A20000, ThermoFisher Scientific) in accordance with the manufacturer's protocols. The *B. tabaci* heads were then incubated with the fluorophore-conjugated serums overnight at 4 °C with a 1:200 dilution, followed by staining with the actin dye phalloidinrhodamine (#A22287, ThermoFisher Scientific) at a 1:500 dilution for 30 min at room temperature and 4′,6-diamidino-2-phenylindole (DAPI) solution (#ab104139, Abcam, Cambridge, USA). Fluorescence images were acquired using a Leica confocal laser-scanning microscope SP8 (Leica Microsystems) to visualize the localization of BtFTSP1.

### Interaction Assays Between Two Proteins

In the Y2H screening assay, the coding sequences of BtFTSP1 without a signal peptide were constructed into the pGBKT7 vector (Clontech, Mountain View, CA, USA) (supplementary Table S5, Supplementary Material online), whereas the cDNA library of *N. benthamiana* was constructed into pGADT7 vector (Biogene Biotech, Shanghai, China). Later, the recombinant vectors were cotransfected into the yeast strain Y2H Gold, and the positive clones were selected on quadruple dropout (QDO) solid medium (SD/−Ade/−His/−Leu/−Try) (#630428, Takara) for approximately 3 d at 30 °C. After colonies were harvested from the QDO liquid medium, the positive yeast plasmids were extracted using the TIANprep Yeast plasmid DNA kit (#DP112-02, TIANGEN, Beijing, China) and subsequently introduced into *E. coli* DH5α competent cells (#TSC-C14, Tsingke), so as to identify the potential interacting genes by Sanger sequencing (YouKang Biotech, Hangzhou, China).

In the Y2H point-to-point verification assay, BtFTSP1, MmFTSP-like, NtFD1, and their, respectively, mutants and were cloned into pGBKT7 or pGADT7 vector, respectively. The MmFTSP-like sequence (XM_025496291) used in this study was synthesized via the custom service of GenScript Company (Nanjing, China). The primers used for vector construction are listed in supplementary Table S5, Supplementary Material online. Afterward, the recombinant vectors and corresponding empty vectors were cotransfected into the yeast strain Y2H Gold, and incubated on the double dropout (DDO) medium (SD/-Leu/-Trp) (#630417, Takara) at 30 °C for 3 d. Then, the monoclonal colonies were spotted on QDO medium. Yeast cells were photographed after 3 d at 30 °C to record growth.

In the Co-IP assays, BtFTSP1 and NtFD1 were cloned into Lic-Myc or Lic-Flag vectors for fusion expression with Myc or Flag. The β-Glucuronidase (GUS) gene fused with Flag was used as a control. *N. benthamiana* leaves (5-wk-old) were transformed by agro-infiltration with the different combinations: BtFTSP1-Myc/NtFD1-Flag, BtFTSP1-Myc/GUS-Flag, NtFD1-Myc/NtFD1-Flag, and NtFD1-Myc/GUS-Flag. Two days later, the leaves were ground in liquid nitrogen and extracted using IP lysis buffer (#87788, Thermo Scientific) with 1 g samples in a 1 ml reaction solution with the addition of a protease inhibitor cocktail (#56079200, Roche, Switzerland). After maintaining them at 4 °C for 10 min, the mixtures were centrifuged at 1,000 × *g* for 20 min. The supernatant was then incubated with 20 μl anti-Flag beads (#L00432-1, GenScript, Nanjing, China) for 4 h at 4 °C. The immunoprecipitates were then washed 4 times with 1×PBS and resuspended in 80 μL 2×SDS-PAGE sample buffer (500 mM Tris–HCl, pH = 6.8, 50% glycerin, 10% SDS, 1% bromophenol blue, and 2% β-mercaptoethanol). Subsequently, the protein samples were boiled at 95 °C for 10 min and subjected to western blotting analysis.

In the BiFC assay, BtFTSP1 and NtFD1 were cloned into the pCV-cYFP or pCV-nYFP vectors. Thereafter, the recombinant vectors were transfected into *A. tumefaciens* GV3101 as described above. Then, the *A. tumefaciens* transfected with recombinant vectors and corresponding empty vectors were co-infiltrated into *N. benthamiana* leaves. The infiltrated *N. benthamiana* was maintained in a climate chamber for 36 to 48 h. YFP fluorescence was captured under the Leica SP8 confocal microscope.

In the LUC assay, BtFTSP1 was cloned into pCAMBIA1300-cLUC vector, whereas the NtFD1 was cloned into pCAMBIA1300-nLUC vector, respectively. The recombinant vectors and corresponding empty vectors were transformed into *A. tumefaciens* GV3101, respectively, which were subsequently co-infiltrated into different areas of the same *N. benthamiana* leaf. At 36 h postinfiltration, 0.2 mM LUC substrate was infiltrated into the whole leaves, and images were obtained using a low-light cooled CDD imaging apparatus (LUMAZONE SOPHIA2048B, USA).

### Interaction Assays Between Three Proteins

In Y3H assays, the full-length CDS sequence of NtFD1 was ligated into the pBridge vector (Clontech) to fusion expression with DNA binding domain (supplementary Table S5, Supplementary Material online). The coding sequences of BtFTSP1 without a signal peptide were cloned into the same pBridge vector which contained the MET25 promoter to drive transcription. The recombinant pBridge vectors, together with the recombinant pGADT7-NtFD1 vector were transfected into the yeast, and incubated on the DDO medium at 30 °C for 3 d. Then, the monoclonal colonies were spotted on QDO and TDO (SD/−Leu/-Trp/-Met) medium, respectively. Yeast cells were photographed after 3 d (on QDO medium) or 5 d (on TDO medium) at 30 °C to record growth.

In competitive BiFC assays, the recombinant vectors of pCV-cYFP-NtFD1, pCV-nYFP-NtFD1, and BtFTSP1-Myc were used. The recombinant vectors, as well as the empty Lic-Myc vector, were transfected into *A. tumefaciens* GV3101, respectively. Then, *A. tumefaciens* carrying pCV-cYFP-NtFD1 and pCV-nYFP-NtFD1 were mixed with different concentrations (OD_600_ = 0.1, 0.3, 1) of *A. tumefaciens* carrying BtFTSP1-Myc, and co-infiltrated into *N. benthamiana* leaves. One day later, the leaves were further infiltrated with 50μM MG132 (#M7449, Sigma-Aldrich, Steinheim, Germany). The infiltrated *N. benthamiana* was maintained in a climate chamber for additional 12 to 24 h. YFP fluorescence was captured under the Leica SP8 confocal microscope.

In pull down assays, the NtFD1-Flag, NtFD1-Myc, BtFTSP1-GFP, and GFP were independently expressed in *N. benthamiana* plant. The leaves were ground in liquid nitrogen and extracted using IP lysis buffer with 1 g samples in a 1 ml reaction solution with the addition of a protease inhibitor cocktail. After maintaining them at 4 °C for 10 min, the mixtures were centrifuged at 1,000 × *g* for 20 min and the supernatant were used for subsequent experiment. Briefly, the supernatant of NtFD1-Flag was incubated with 20 μl anti-Flag beads for 2 h at 4 °C. After washing with PBST (consisting of PBS and 0.1% Triton-100 (#A110694, Sango Biotechnology) for 3 times, the beads were incubated with a mixture of NtFD1-Myc/BtFTSP1-GFP and NtFD1-Myc/GFP, respectively. The beads were further washed with PBST for 4 times, and the precipitate was added with protein loading buffer and subjected to western blotting analysis.

### Detection of NtFD1 Polymer

The *N. benthamiana* leaves expressing NtFD1-Flag were ground in liquid nitrogen and extracted using IP lysis buffer with 1 g samples in a 1 ml reaction solution with the addition of a protease inhibitor cocktail. The samples were directly loaded onto native PAGE gel and underwent western blotting analysis. The native protein marker GAPDH (#G5262-1VL, Sigma-Aldrich, St. Louis, MO, USA) was used to determine the protein size.

### BtFTSP1 Destabilizes NtFD1 Assay

The recombinant vector of NtFD1-flag, BtFTSP1-Myc, and MmFTSP-like-Myc were used. The recombinant vectors were transfected into *A. tumefaciens* GV3101, respectively. Then, *A. tumefaciens* carrying NtFD1-Flag were mixed with different concentrations (OD_600_ = 0.1, 0.3, 1) of *A. tumefaciens* carrying BtFTSP1-Myc or MmFTSP-like-Myc, and co-infiltrated into *N. benthamiana* leaves. To inhibit ubiquitin-dependent protein degradation, 50 μM MG132 was infiltrated into *N. benthamiana* leaves one day later. The infiltrated *N. benthamiana* was maintained in a climate chamber for 48 h. The protein levels were detected by western blotting analysis.

### Western-Blotting Assay

To guarantee an equal amount of protein loading, the protein concentration was determined by a BCA Protein Assay Kit (#CW0014S, CwBiotech, Taizhou, China) according to the manufacturer's instructions. The samples were separated by 10%, 12.5%, or 15% SDS-PAGE gels and transferred to PVDF membranes. Then, the blots were probed with anti-Flag (1:10,000, #MA1-91878, ThermoFisher Scientific), anti-Myc (1:10,000, #MA1-21316, ThermoFisher Scientific), anti-GFP (1:10,000, #MA5-15256, ThermoFisher Scientific), or anti-BtFTSP1 serum (1:5000, Huaan Biotechnology Company, Hangzhou, China), followed by additional incubation with horseradish peroxidase (HRP)-conjugated goat antimouse IgG antibody (1:10,000, # 31430, ThermoFisher Scientific) or HRP-conjugated goat antirabbit IgG antibody (1:10,000, #31460, ThermoFisher Scientific). Images were acquired by an AI 680 image analyzer (Amersham Pharmacia Biotech, Buckinghamshire, UK). Additionally, samples were stained with Coomassie brilliant blue (CBB) to monitor the protein loading.

### Callose Staining

The callose was detected by aniline blue staining. Briefly, *N. tabacum* leaf was infiltrated with 2 mg/ml aniline blue (Biosupplies, Parkville, Australia; dissolved in 1×PBS, pH 7.5), and incubated in the dark for 5 min. Then, the injected leaf tissue was dissected out, washed with sterile water, and observed using a Leica confocal laser-scanning microscope SP8.

### Diaminobenzidine (DAB) Staining

The H_2_O_2_ level in *N. tabacum* was detected by DAB staining. Briefly, *N. tabacum* leaf was cut and immersed into 1 mg/mL 3,3′-Diaminobenzidine Tetrahydrochloride (DAB-HCl) (pH 3.8, #D8001, Sigma, St. Louis, MO, USA) for 6 h. Thereafter, the DAB solution was replaced with 100% ethanol and decolored overnight at 65 °C. Then, the stained leaf was photographed using a Canon EOS 80D camera (Canon Inc., Tokyo, Japan).

### Statistical Analysis

The log-rank test (SPSS Statistics 19, Chicago, IL, USA) was used to determine the statistical significance of survival distributions. Two-tailed unpaired Student’s *t* test (comparisons between 2 groups) or 1-way ANOVA test followed by Tukey's multiple comparisons test (comparisons among 3 groups) was used to analyze the results of qRT-PCR, EPG, fecundity analysis, and host choice analysis. Data were graphed in GraphPad Prism 9.

## Supplementary Material

msad221_Supplementary_Data

## Data Availability

The sequencing data generated in this study have been deposited in the NCBI Sequence Read Archive under accession number PRJNA947676. All sequence data used in this study can be found in supplementary Table S3 and Data S1, Supplementary Material online.
