# Supplementary material for: Horizontally Transferred Salivary Protein Promotes Insect Feeding by Suppressing Ferredoxin-Mediated Plant Defenses
Source: Mol Biol Evol. 2023 Oct 7;40(10):msad221. doi: 10.1093/molbev/msad221 (PMC10583550; doi:10.1093/molbev/msad221)
Supplement: msad221_Supplementary_Data [file msad221_supplementary_data.zip › Supplementary Data 2.pdf]

Supplementary Data 2 Differentially expressed genes between *Nicotiana tabacum* infested by dsGFP- and dsBtFTSP1- treated *Bemisia tabaci*

Gene_ID	Description	Mean FPKM (dsGFP)	Mean FPKM (dsBtFTSP1)	log2FC	P-Value
Nitab4.5_0001921g0020	Phosphate-induced protein 1	0.00	13.92	-7.89	0.00000
Nitab4.5_0001409g0220		0.00	10.87	-7.54	0.00194
Nitab4.5_0006358g0080		0.00	8.39	-7.16	0.00007
Nitab4.5_0001410g0050	Transposase, MuDR, plant	0.00	8.07	-7.11	0.00023
Nitab4.5_0000059g0330		0.00	7.84	-7.06	0.00000
Nitab4.5_0000028g0480		0.00	7.79	-7.06	0.00092
Nitab4.5_0003214g0010		0.00	7.66	-7.03	0.00310
Nitab4.5_0000586g0060	P-loop containing nucleoside triphosphate hydrolase	0.00	7.61	-7.02	0.00110
Nitab4.5_0001531g0050	Tify, CO/COL/TOC1, conserved site	0.00	6.55	-6.81	0.01013
Nitab4.5_0000119g0320	Ribosome biogenesis protein Nop16	0.00	6.47	-6.79	0.02305
Nitab4.5_0008337g0090	Reverse transcriptase	0.00	6.34	-6.76	0.01265
Nitab4.5_0006598g0030	Auxin-induced protein, ARG7	0.00	6.13	-6.71	0.01717
Nitab4.5_0000980g0390	Auxin-induced protein, ARG7	0.00	6.00	-6.68	0.00080
Nitab4.5_0000639g0190	VQ	0.00	5.98	-6.68	0.00079
Nitab4.5_0006421g0020	C2 domain	0.00	5.94	-6.67	0.00329
Nitab4.5_0002324g0020	Retrotransposon gag domain	0.00	5.79	-6.63	0.00000
Nitab4.5_0003011g0030	Nucleic acid-binding, OB-fold	0.00	5.71	-6.62	0.03184
Nitab4.5_0008975g0030		0.00	5.69	-6.61	0.00105
Nitab4.5_0000319g0100		0.38	39.83	-6.56	0.00000
Nitab4.5_0002492g0060		0.00	5.52	-6.56	0.00001
Nitab4.5_0007722g0030		0.00	5.22	-6.48	0.00540
Nitab4.5_0002640g0020	VQ	0.00	5.13	-6.46	0.00655
Nitab4.5_0000452g0070		0.00	4.94	-6.40	0.00016
Nitab4.5_0011440g0020	Ribonuclease H-like domain	0.00	4.84	-6.37	0.01213
Nitab4.5_0001858g0020		0.00	4.42	-6.24	0.00042
Nitab4.5_0003378g0050	NB-ARC	0.00	4.11	-6.14	0.01643
Nitab4.5_0002878g0050		0.00	3.75	-6.00	0.02536
Nitab4.5_0000860g0200	EXTL2, alpha-1,4-N-acetylhexosaminyltransferase	0.00	3.45	-5.88	0.00310
Nitab4.5_0006270g0030	Protein of unknown function DUF3743	0.00	3.15	-5.76	0.00298
Nitab4.5_0008522g0060	Heat shock protein Hsp90, N-terminal, Heat shock protein Hsp90 family, His	0.09	6.53	-5.68	0.00099
Nitab4.5_0006504g0010	Aminotransferase-like, plant mobile domain	0.00	2.79	-5.57	0.00424

Nitab4.5_0024858g0010	Powdery mildew resistance protein, RPW8 domain	0.00	2.75	-5.55	0.00424
Nitab4.5_0005360g0010		0.51	25.47	-5.54	0.00000
Nitab4.5_0001083g0010	AP2/ERF domain, DNA-binding domain	0.05	3.51	-5.38	0.00693
Nitab4.5_0011739g0010	Alpha crystallin/Hsp20 domain, HSP20-like chaperone	0.09	4.25	-5.10	0.00191
Nitab4.5_0005372g0100	Bromo adjacent homology (BAH) domain	0.00	1.96	-5.06	0.01300
Nitab4.5_0001589g0010		0.22	8.11	-4.98	0.00142
Nitab4.5_0011008g0030		0.52	16.22	-4.86	0.00002
Nitab4.5_0000185g0110	AP2/ERF domain, DNA-binding domain	0.07	3.17	-4.80	0.00185
Nitab4.5_0005246g0010	Tify, CO/COL/TOC1, conserved site	0.13	3.59	-4.43	0.01476
Nitab4.5_0000525g0160		0.09	2.29	-4.40	0.00743
Nitab4.5_0001921g0030	Phosphate-induced protein 1	2.38	46.30	-4.28	0.00000
Nitab4.5_0000568g0010	EF-Hand 1, calcium-binding site, EF-hand domain, EF-hand domain pair	1.25	23.78	-4.25	0.00000
Nitab4.5_0003137g0030		0.39	8.00	-4.23	0.04916
Nitab4.5_0008134g0020		1.01	19.68	-4.22	0.00000
Nitab4.5_0000110g0010		0.31	6.57	-4.22	0.00003
Nitab4.5_0000698g0030		0.37	7.19	-4.15	0.00409
Nitab4.5_0004874g0010	Phosphate-induced protein 1	0.44	7.79	-4.14	0.00002
Nitab4.5_0004829g0120		0.23	4.33	-4.02	0.01586
Nitab4.5_0011791g0010	Rapid ALkalinization Factor	0.68	11.13	-3.99	0.02567
Nitab4.5_0001223g0120	EF-hand domain pair, EF-hand domain, EF-Hand 1, calcium-binding site	0.18	3.45	-3.95	0.01835
Nitab4.5_0002980g0030	EF-hand domain, EF-Hand 1, calcium-binding site, EF-hand domain pair	0.86	12.44	-3.83	0.00023
Nitab4.5_0000857g0050		2.32	32.69	-3.82	0.00000
Nitab4.5_0000852g0120	Zinc finger, RING-type, Zinc finger, RING/FYVE/PHD-type	0.67	9.49	-3.78	0.00022
Nitab4.5_0000015g0110		0.85	11.90	-3.74	0.01680
Nitab4.5_0000974g0060	Lateral organ boundaries, LOB	0.73	10.02	-3.74	0.00199
Nitab4.5_0000134g0120		0.38	5.23	-3.65	0.04139
Nitab4.5_0002684g0110		0.36	4.98	-3.64	0.01605
Nitab4.5_0000192g0090	Gibberellin regulated protein	2.20	27.46	-3.63	0.00000
Nitab4.5_0000357g0200	Zinc finger, RING-type, Zinc finger, RING/FYVE/PHD-type	0.48	6.03	-3.59	0.00305
Nitab4.5_0001671g0150		0.47	6.03	-3.56	0.00072
Nitab4.5_0002329g0010	Leucine-rich repeat	2.06	24.35	-3.55	0.00048
Nitab4.5_0015041g0010		0.78	9.47	-3.53	0.02955
Nitab4.5_0006188g0010		0.53	6.34	-3.48	0.00354
Nitab4.5_0004511g0060		1.74	19.57	-3.47	0.00002
Nitab4.5_0000003g0750	Homeodomain-like, Myb-like domain, SANT/Myb domain, Myb domain	0.39	4.51	-3.47	0.00044
Nitab4.5_0007168g0030		1.99	22.40	-3.46	0.00451
Nitab4.5_0003346g0010	Cytochrome P450, Cytochrome P450, conserved site, Cytochrome P450, E-	0.35	3.83	-3.44	0.00049
Nitab4.5_0000605g0240	Proteinase inhibitor I29, cathepsin propeptide, Peptidase C1A, papain C-terr	0.31	3.54	-3.43	0.00336
Nitab4.5_0002852g0020	DNA-binding domain, AP2/ERF domain	0.90	9.83	-3.42	0.00188

Nitab4.5_0000518g0090	Cupredoxin, Multicopper oxidase, type 2, Multicopper oxidase, type 1, L-asc	0.44	4.71	-3.42	0.00216
Nitab4.5_0001229g0080		1.32	14.43	-3.42	0.01899
Nitab4.5_0000004g0330	P-loop containing nucleoside triphosphate hydrolase, Small GTPase superfa	0.99	10.42	-3.37	0.00000
Nitab4.5_0002171g0070	Basic-leucine zipper domain	1.02	10.31	-3.33	0.00000
Nitab4.5_0002312g0100		3.60	34.34	-3.26	0.00000
Nitab4.5_0003624g0060		0.76	7.42	-3.24	0.01966
Nitab4.5_0002310g0170		0.58	5.71	-3.24	0.00282
Nitab4.5_0020114g0010	Cytochrome P450, Cytochrome P450, E-class, group I, Cytochrome P450, c	7.33	69.35	-3.24	0.00016
Nitab4.5_0004646g0080		5.90	54.16	-3.20	0.00000
Nitab4.5_0001151g0010		1.73	16.03	-3.20	0.00072
Nitab4.5_0002630g0010	Xyloglucan endo-transglycosylase, C-terminal, Beta-glucanase, Concanaval	0.65	6.08	-3.18	0.00036
Nitab4.5_0003235g0090	Protein kinase-like domain, Serine-threonine/tyrosine-protein kinase catalytic	0.65	6.01	-3.17	0.00725
Nitab4.5_0005755g0010	Phosphate-induced protein 1	2.62	23.31	-3.15	0.00000
Nitab4.5_0003402g0050		0.39	3.60	-3.13	0.01014
Nitab4.5_0001675g0070	Xyloglucan endo-transglycosylase, C-terminal, Xyloglucan endotransglucosy	0.48	4.03	-3.09	0.00394
Nitab4.5_0000381g0180		0.46	4.16	-3.05	0.00405
Nitab4.5_0007098g0010		0.73	5.93	-3.02	0.00416
Nitab4.5_0002503g0010		4.48	36.30	-3.02	0.00000
Nitab4.5_0000047g0140	DNA-binding WRKY	1.49	12.01	-3.01	0.00046
Nitab4.5_0010755g0030	Auxin-induced protein, ARG7	6.32	50.21	-2.99	0.01048
Nitab4.5_0002503g0070	AP2/ERF domain, DNA-binding domain	1.29	10.16	-2.97	0.00790
Nitab4.5_0007486g0030	Retrotransposon gag domain	0.51	4.28	-2.97	0.00936
Nitab4.5_0001013g0070	Pectin lyase fold/virulence factor, Pectinesterase, catalytic, Pectin lyase fold	0.71	5.62	-2.95	0.00395
Nitab4.5_0009673g0010		0.57	4.60	-2.93	0.00339
Nitab4.5_0013465g0010	Xyloglucan endo-transglycosylase, C-terminal, Concanavalin A-like lectin/glu	1.86	14.13	-2.93	0.00009
Nitab4.5_0012198g0010	Cytochrome P450, Cytochrome P450, conserved site, Cytochrome P450, E-	1.05	7.83	-2.91	0.00026
Nitab4.5_0004709g0010	Carboxypeptidase A inhibitor-like	0.95	7.04	-2.90	0.00046
Nitab4.5_0026398g0010		1.05	7.70	-2.88	0.00112
Nitab4.5_0000130g0010		0.90	6.61	-2.87	0.00291
Nitab4.5_0003313g0050	Dehydrin	3.92	28.71	-2.87	0.00000
Nitab4.5_0012054g0010		2.78	20.36	-2.87	0.00000
Nitab4.5_0018681g0010	Serine/threonine-protein kinase, active site, Protein kinase, ATP binding site	1.37	9.82	-2.84	0.00002
Nitab4.5_0002885g0120	ATPase, F1 complex, epsilon subunit, mitochondrial	0.99	7.20	-2.83	0.00153
Nitab4.5_0000304g0050		1.56	11.12	-2.80	0.01448
Nitab4.5_0004577g0030	Zinc finger, RING/FYVE/PHD-type, Zinc finger, RING-type	0.50	3.60	-2.80	0.00336
Nitab4.5_0000073g0450	Ribosomal protein S17, Nucleic acid-binding, OB-fold	1.22	8.61	-2.79	0.00002
Nitab4.5_0000414g0110	Terpene synthase, N-terminal domain, Terpenoid cyclases/protein prenyltrar	3.66	25.29	-2.79	0.00470
Nitab4.5_0001780g0030		2.86	20.01	-2.79	0.00551
Nitab4.5_0001239g0170	ATPase, AAA-type, conserved site, AAA-type ATPase, N-terminal domain, A	1.49	10.10	-2.76	0.00081

Nitab4.5_0028615g0010	Auxin-induced protein, ARG7	1.53	10.42	-2.75	0.00012
Nitab4.5_0000048g0130		0.98	6.88	-2.75	0.00032
Nitab4.5_0015114g0020	Heat shock protein 70, conserved site, Heat shock protein 70 family	3.79	25.38	-2.74	0.00000
Nitab4.5_0003894g0070		1.15	7.79	-2.73	0.03173
Nitab4.5_0008226g0020	Terpenoid synthase, Terpene synthase, N-terminal domain, Terpenoid cycla	1.79	11.82	-2.73	0.01423
Nitab4.5_0000160g0050		6.45	41.97	-2.70	0.00550
Nitab4.5_0006692g0010	Cytochrome P450, Cytochrome P450, conserved site, Cytochrome P450, E-	7.92	51.23	-2.69	0.00055
Nitab4.5_0005769g0060	Clp protease proteolytic subunit /Translocation-enhancing protein TepA	1.01	6.67	-2.69	0.01429
Nitab4.5_0001317g0160	Basic-leucine zipper domain	0.96	6.19	-2.69	0.00359
Nitab4.5_0000244g0350	Concanavalin A-like lectin/glucanase, subgroup, Glycoside hydrolase, family	0.93	5.99	-2.69	0.00142
Nitab4.5_0012177g0010		1.00	6.46	-2.66	0.01903
Nitab4.5_0007460g0010		0.98	6.17	-2.66	0.00631
Nitab4.5_0008474g0010	Lateral organ boundaries, LOB	1.66	10.47	-2.66	0.00005
Nitab4.5_0001800g0020	Heat shock protein 70, conserved site, Heat shock protein 70 family	2.43	15.21	-2.65	0.00054
Nitab4.5_0001301g0070	Zinc finger, RING-CH-type, Protein of unknown function DUF3675, Zinc finge	7.20	44.84	-2.64	0.00000
Nitab4.5_0000223g0340	DNA-binding WRKY	2.36	14.62	-2.63	0.00326
Nitab4.5_0000786g0240	Phosphate-induced protein 1	0.81	5.00	-2.62	0.00202
Nitab4.5_0007566g0010	Cytochrome P450, conserved site, Cytochrome P450, Cytochrome P450, E-	12.92	79.11	-2.61	0.00052
Nitab4.5_0005034g0010	Pectinesterase inhibitor domain	0.67	4.07	-2.60	0.00979
Nitab4.5_0008226g0010	Terpene synthase, metal-binding domain, Terpenoid synthase	3.93	23.87	-2.60	0.00872
Nitab4.5_0013643g0010	Uncharacterised conserved protein UCP037471, Cytochrome b561/ferric rec	1.34	8.09	-2.60	0.00856
Nitab4.5_0000406g0070	Acyl-CoA N-acyltransferase, GNAT domain	1.75	10.42	-2.57	0.00104
Nitab4.5_0007001g0020	Homeobox domain, Homeodomain-like, Homeobox, conserved site, Helix-tu	5.87	34.75	-2.56	0.00000
Nitab4.5_0001666g0010		3.37	19.88	-2.56	0.00006
Nitab4.5_0006835g0010		3.72	21.97	-2.56	0.00000
Nitab4.5_0000027g0570		0.87	5.12	-2.55	0.00860
Nitab4.5_0018164g0010	Protein of unknown function DUF1005	1.01	5.86	-2.54	0.00254
Nitab4.5_0001476g0100	Cytochrome P450, Cytochrome P450, B-class, Cytochrome P450, conserve	0.94	5.53	-2.53	0.00682
Nitab4.5_0001522g0010	CCT domain	0.81	4.77	-2.52	0.00906
Nitab4.5_0008797g0010		5.89	33.75	-2.51	0.00003
Nitab4.5_0005728g0080		4.79	27.05	-2.50	0.00000
Nitab4.5_0001177g0010	Target SNARE coiled-coil domain, Syntaxin, N-terminal domain, Syntaxin/ep	2.67	15.00	-2.49	0.00057
Nitab4.5_0000286g0060	Alpha crystallin/Hsp20 domain, HSP20-like chaperone	6.29	35.28	-2.49	0.00002
Nitab4.5_0000460g0100	Homeobox domain, Homeodomain-like, Helix-turn-helix motif, Homeobox, cc	1.07	5.99	-2.49	0.00567
Nitab4.5_0001760g0080		5.52	30.63	-2.47	0.00000
Nitab4.5_0002113g0180	Multiprotein bridging factor 1, N-terminal, Cro/C1-type helix-turn-helix domair	7.45	41.24	-2.47	0.00177
Nitab4.5_0000463g0020	Heat shock protein 70 family, Heat shock protein 70, conserved site	1.46	7.99	-2.46	0.01233
Nitab4.5_0002687g0010	Tetracycline resistance protein, TetA/multidrug resistance protein MdtG, Maj	2.52	13.73	-2.45	0.00077
Nitab4.5_0004251g0010	Homeodomain-like, Homeobox domain, Homeobox, conserved site, Helix-tu	6.16	33.56	-2.45	0.00000

Nitab4.5_0000662g0020		2.80	15.18	-2.44	0.00108
Nitab4.5_0005982g0020	Dormancyauxin associated	62.70	337.40	-2.43	0.00000
Nitab4.5_0004738g0080		6.41	34.36	-2.42	0.00000
Nitab4.5_0005578g0010		2.05	11.07	-2.42	0.00000
Nitab4.5_0000760g0030	Oligopeptide transporter, OPT superfamily	1.74	9.43	-2.41	0.00002
Nitab4.5_0004465g0060		4.19	22.00	-2.39	0.00222
Nitab4.5_0002694g0030	O-methyltransferase, family 3	1.45	7.63	-2.38	0.00573
Nitab4.5_0001128g0040	Zinc finger, RING-type, Zinc finger, C6HC-type, Zinc finger, RING/FYVE/PHI	1.15	5.97	-2.37	0.01270
Nitab4.5_0018811g0010	O-methyltransferase, family 3	2.63	13.60	-2.37	0.00053
Nitab4.5_0001118g0040		1.82	9.38	-2.37	0.02128
Nitab4.5_0004584g0010	SWEET sugar transporter	1.16	5.94	-2.36	0.00080
Nitab4.5_0013007g0030	Terpene synthase, N-terminal domain, Terpenoid cyclases/protein prenyltrar	3.05	15.63	-2.36	0.02636
Nitab4.5_0007490g0050		2.11	10.85	-2.35	0.02642
Nitab4.5_0000028g0180	Cupredoxin, Plastocyanin-like, Blue (type 1) copper domain	18.22	92.14	-2.34	0.00036
Nitab4.5_0004055g0070	Conserved hypothetical protein CHP01589, plant	3.72	18.88	-2.34	0.02320
Nitab4.5_0004904g0010		13.55	68.25	-2.33	0.00000
Nitab4.5_0000833g0010	Concanavalin A-like lectin/glucanases superfamily, Concanavalin A-like lecti	2.90	14.60	-2.33	0.00000
Nitab4.5_0000207g0450	EF-hand domain pair	19.74	98.94	-2.33	0.00011
Nitab4.5_0008180g0060		2.06	10.30	-2.32	0.00012
Nitab4.5_0007099g0040	DNA-binding WRKY	1.52	7.56	-2.32	0.02841
Nitab4.5_0016435g0010	Terpene synthase, metal-binding domain, Terpenoid synthase, Terpene synt	5.13	25.47	-2.31	0.01734
Nitab4.5_0000368g0350	Transcription factor GRAS	2.44	12.06	-2.31	0.00077
Nitab4.5_0012264g0010		8.42	40.94	-2.28	0.00003
Nitab4.5_0004833g0020	Plastocyanin-like, Cupredoxin, Blue (type 1) copper domain	12.26	59.39	-2.28	0.00120
Nitab4.5_0007641g0020	Sucrose synthase, plant/cyanobacteria, Sucrose synthase, Glycosyl transfer	2.64	12.48	-2.24	0.00000
Nitab4.5_0012394g0010	Epoxide hydrolase-like	2.13	10.07	-2.24	0.00574
Nitab4.5_0003888g0010		14.41	68.05	-2.24	0.00052
Nitab4.5_0000404g0110	Xyloglucan endotransglucosylase/hydrolase, Beta-glucanase, Xyloglucan en	2.47	11.51	-2.22	0.00000
Nitab4.5_0003715g0060	Rossmann-like alpha/beta/alpha sandwich fold, UspA, Universal stress prote	1.08	4.98	-2.20	0.00315
Nitab4.5_0012978g0010		3.04	13.99	-2.19	0.01893
Nitab4.5_0004775g0010	Domain of unknown function DUF3700	1.48	6.72	-2.18	0.00206
Nitab4.5_0000194g0160	START-like domain, Bet v I type allergen, Bet v I domain	3.30	14.95	-2.18	0.00017
Nitab4.5_0003711g0050	Protein of unknown function DUF4228, plant	3.47	15.65	-2.17	0.00426
Nitab4.5_0017332g0010	DNA-directed RNA polymerase, subunit RPB6, Archaeal RpoK/eukaryotic R	1.39	6.17	-2.15	0.00046
Nitab4.5_0000570g0370	Endosulphine	2.00	8.64	-2.11	0.00008
Nitab4.5_0006549g0010	DNA-binding WRKY	1.78	7.68	-2.11	0.00315
Nitab4.5_0001237g0010	GNAT domain, Acyl-CoA N-acyltransferase	3.75	16.08	-2.10	0.00413
Nitab4.5_0003032g0010	DnaJ domain, DnaJ domain, conserved site, HSP40/DnaJ peptide-binding, C	12.30	52.67	-2.10	0.00007
Nitab4.5_0005805g0050	Late embryogenesis abundant protein, LEA-5	8.85	37.91	-2.10	0.00285

Nitab4.5_0005040g0030		17.55	74.98	-2.09	0.00008
Nitab4.5_0008196g0010	Thiolase-like, subgroup, Very-long-chain 3-ketoacyl-CoA synthase, FAE1/Ty	9.27	39.49	-2.09	0.00001
Nitab4.5_0000732g0120	Dienelactone hydrolase	1.29	5.48	-2.09	0.00731
Nitab4.5_0000025g0420	Oleosin	4.83	20.49	-2.08	0.00000
Nitab4.5_0001461g0050	Terpenoid synthase, Terpene synthase, metal-binding domain, Terpenoid cy	5.24	22.05	-2.07	0.02770
Nitab4.5_0012964g0030	Ribosomal protein L41	3.61	15.19	-2.07	0.00053
Nitab4.5_0004685g0090	Zinc finger, RING-type, Zinc finger, RING/FYVE/PHD-type	7.55	31.73	-2.07	0.00009
Nitab4.5_0001865g0010	Homeobox domain, Homeodomain-like, Leucine zipper, homeobox-associat	2.11	8.87	-2.07	0.00010
Nitab4.5_0003228g0050	Peptidase S8, subtilisin-related	2.05	8.67	-2.07	0.00059
Nitab4.5_0007571g0020	AP2/ERF domain, DNA-binding domain	2.68	11.17	-2.06	0.00333
Nitab4.5_0008653g0010		45.00	187.56	-2.06	0.00000
Nitab4.5_0002717g0050	Terpenoid synthase, Terpene synthase, metal-binding domain, Terpenoid cy	8.03	33.37	-2.06	0.02815
Nitab4.5_0008811g0010	Protein of unknown function DUF3675, Zinc finger, RING/FYVE/PHD-type	2.10	8.70	-2.05	0.00296
Nitab4.5_0000902g0020		9.14	37.81	-2.05	0.00799
Nitab4.5_0003599g0030	Ribosomal protein L27/L41, mitochondrial	8.16	33.62	-2.04	0.00000
Nitab4.5_0003524g0040	Heavy metal-associated domain, HMA	7.48	30.71	-2.04	0.00001
Nitab4.5_0000341g0020	Pectin lyase fold/virulence factor, Pectinesterase, catalytic, Pectin lyase fold	4.61	18.88	-2.03	0.00000
Nitab4.5_0002076g0030	Protein of unknown function DUF4228, plant	15.22	62.25	-2.03	0.00000
Nitab4.5_0001077g0040	NAC domain	5.45	22.13	-2.02	0.00000
Nitab4.5_0021859g0010	UspA, Rossmann-like alpha/beta/alpha sandwich fold, Universal stress prote	3.72	15.02	-2.01	0.00021
Nitab4.5_0000734g0070	DNA-binding domain, AP2/ERF domain	8.85	35.68	-2.01	0.00004
Nitab4.5_0001226g0020	DNA-binding WRKY	2.26	9.00	-1.99	0.01779
Nitab4.5_0001655g0230		5.39	21.40	-1.99	0.00002
Nitab4.5_0000040g0600		3.90	15.57	-1.99	0.01901
Nitab4.5_0001000g0160	DNA-binding WRKY	8.08	32.02	-1.99	0.00580
Nitab4.5_0018523g0020	Photosystem antenna protein-like	4.48	17.73	-1.98	0.00290
Nitab4.5_0007814g0010	P-loop containing nucleoside triphosphate hydrolase	5.27	20.81	-1.98	0.00000
Nitab4.5_0000286g0080	DNA-binding WRKY	14.70	57.82	-1.98	0.00208
Nitab4.5_0007943g0030	Domain of unknown function DUF239	5.09	20.09	-1.97	0.01708
Nitab4.5_0004974g0060	F-box domain, F-box associated interaction domain, F-box associated doma	1.57	6.15	-1.97	0.00566
Nitab4.5_0000040g0110	NAC domain	3.01	11.78	-1.97	0.00006
Nitab4.5_0000319g0160		40.30	156.49	-1.96	0.00008
Nitab4.5_0001105g0070		5.31	20.54	-1.95	0.00392
Nitab4.5_0004024g0020		4.40	17.03	-1.95	0.00527
Nitab4.5_0000786g0210	Phosphate-induced protein 1	9.21	35.47	-1.95	0.00006
Nitab4.5_0002574g0060	Domain of unknown function DUF1785, Stem cell self-renewal protein Piwi, /	3.35	12.91	-1.95	0.00394
Nitab4.5_0003838g0090	Pentatricopeptide repeat, Glycosyl hydrolase family 100, Six-hairpin glycosid	3.65	14.05	-1.94	0.00001
Nitab4.5_0001957g0020	DnaJ domain, HSP40/DnaJ peptide-binding, Chaperone DnaJ, C-terminal	5.74	22.00	-1.94	0.00289
Nitab4.5_0002350g0090	Serine/threonine-protein kinase, active site, Protein kinase domain, Serine-tl	3.24	12.35	-1.93	0.00102

Nitab4.5_0011263g0010		3.11	11.94	-1.93	0.01294
Nitab4.5_0002328g0020	Heat shock transcription factor, plant, Heat shock factor (HSF)-type, DNA-bi	5.17	19.73	-1.93	0.00016
Nitab4.5_0001566g0040		4.52	17.18	-1.93	0.04684
Nitab4.5_0011381g0050	Oligopeptide transporter, OPT superfamily	4.48	17.04	-1.93	0.00000
Nitab4.5_0006164g0040	Mitochondrial substrate/solute carrier, Mitochondrial carrier domain, Mitocho	1.73	6.57	-1.93	0.00307
Nitab4.5_0004338g0090		1.75	6.65	-1.92	0.00617
Nitab4.5_0002700g0030		9.48	35.78	-1.92	0.00000
Nitab4.5_0018289g0010		6.91	25.94	-1.91	0.00264
Nitab4.5_0007535g0020		6.28	23.51	-1.90	0.00129
Nitab4.5_0002046g0110		24.07	90.10	-1.90	0.00394
Nitab4.5_0003981g0080	Alpha crystallin/Hsp20 domain, HSP20-like chaperone	3.47	13.00	-1.90	0.00022
Nitab4.5_0001216g0110	Cytochrome b-c1 complex subunit 9	14.93	55.62	-1.90	0.00001
Nitab4.5_0000736g0050	Protein of unknown function DUF1645, plant	5.64	20.87	-1.89	0.00010
Nitab4.5_0000031g0040	SGNH hydrolase-type esterase domain	6.36	23.58	-1.89	0.00856
Nitab4.5_0000313g0020	NAC domain	3.06	11.22	-1.87	0.00005
Nitab4.5_0000703g0110	Cation-transporting P-type ATPase, C-terminal, Fatty acid hydroxylase	29.29	107.33	-1.87	0.00011
Nitab4.5_0002718g0050	Phloem protein 2-like, F-box domain	5.87	21.48	-1.87	0.00000
Nitab4.5_0005133g0020	Heat shock protein 70, conserved site, Heat shock protein 70 family	4.00	14.65	-1.87	0.00309
Nitab4.5_0001615g0010	BTB/POZ fold, WD40/YVTN repeat-like-containing domain, WD40-repeat-co	2.01	7.32	-1.87	0.01509
Nitab4.5_0002470g0040		3.92	14.28	-1.87	0.00164
Nitab4.5_0000886g0010	DNA-binding WRKY	5.04	18.36	-1.86	0.00243
Nitab4.5_0000600g0050	AP2/ERF domain, DNA-binding domain	5.63	20.50	-1.86	0.00032
Nitab4.5_0005560g0020	CBS domain, Aldolase-type TIM barrel	11.97	43.14	-1.85	0.00000
Nitab4.5_0008334g0040	Late embryogenesis abundant protein, LEA-25/LEA-D113	14.17	50.89	-1.84	0.00000
Nitab4.5_0004330g0030	Non-haem dioxygenase N-terminal domain, Oxoglutarate/iron-dependent dic	53.97	193.64	-1.84	0.00525
Nitab4.5_0003786g0020	Peptidase M10A, Peptidoglycan binding-like, Peptidase M10, metallopeptida	4.35	15.59	-1.84	0.01264
Nitab4.5_0007620g0010	Zinc finger, C2H2	11.86	42.44	-1.84	0.00013
Nitab4.5_0000028g0360		5.01	17.94	-1.84	0.01075
Nitab4.5_0001190g0010		75.20	269.10	-1.84	0.00204
Nitab4.5_0000183g0080	FBD domain, F-box domain	2.01	7.18	-1.84	0.00241
Nitab4.5_0014335g0010	C2 domain	2.14	7.65	-1.84	0.00386
Nitab4.5_0010600g0020	Uncharacterised protein family Cys-rich	5.09	18.16	-1.84	0.00634
Nitab4.5_0004300g0180	NAC domain	9.34	33.22	-1.83	0.00013
Nitab4.5_0005728g0010		2.25	7.94	-1.82	0.00187
Nitab4.5_0000500g0040	Aromatic-ring hydroxylase-like, Monooxygenase, FAD-binding	3.54	12.45	-1.81	0.01548
Nitab4.5_0001413g0010	Protein of unknown function DUF1645, plant	7.90	27.73	-1.81	0.00040
Nitab4.5_0002815g0140	UDP-glucuronosyl/UDP-glucosyltransferase	4.02	14.10	-1.81	0.01653
Nitab4.5_0000130g0140	Non-haem dioxygenase N-terminal domain, Isopenicillin N synthase-like, Ox	35.37	123.53	-1.80	0.01013
Nitab4.5_0002373g0050		3.92	13.69	-1.80	0.02118

Nitab4.5_0000270g0260	Histone-fold, Histone core, Histone H4, conserved site, Histone H4	3.12	10.84	-1.80	0.00973
Nitab4.5_0000287g0240	Basic-leucine zipper domain	11.20	38.92	-1.80	0.00000
Nitab4.5_0002456g0060	Protein of unknown function DUF4228, plant	5.12	17.66	-1.79	0.03502
Nitab4.5_0012523g0010	UDP-glucuronosyl/UDP-glucosyltransferase	5.09	17.49	-1.78	0.03565
Nitab4.5_0007691g0010	DNA-binding WRKY	10.61	36.39	-1.78	0.00973
Nitab4.5_0000036g0570		5.50	18.84	-1.78	0.00000
Nitab4.5_0003044g0040	Six-hairpin glycosidase-like, Glycosyl hydrolase family 100	6.15	20.98	-1.77	0.00000
Nitab4.5_0000723g0160	Dehydrin	12.89	43.99	-1.77	0.00000
Nitab4.5_0000286g0090	Alpha crystallin/Hsp20 domain, HSP20-like chaperone	2.61	8.86	-1.76	0.01484
Nitab4.5_0005939g0020	Syntaxin/epimorphin, conserved site, Target SNARE coiled-coil domain, t-SN	10.20	34.63	-1.76	0.01230
Nitab4.5_0002546g0080	Cytochrome c oxidase biogenesis protein Cmc1-like	8.74	29.61	-1.76	0.00117
Nitab4.5_0000011g0100	Zinc finger, CHCC-type	22.37	75.51	-1.76	0.00142
Nitab4.5_0001933g0010	Homeodomain-like, Myb domain, plants, Myb domain, SANT/Myb domain	2.76	9.29	-1.75	0.00280
Nitab4.5_0000004g0320	Ribosomal protein L23/L15e core domain, Nucleotide-binding, alpha-beta pla	5.95	20.04	-1.75	0.00002
Nitab4.5_0000312g0130	Alpha crystallin/Hsp20 domain, HSP20-like chaperone	7.26	24.34	-1.74	0.00066
Nitab4.5_0000619g0010	Rapid ALkalinization Factor	4.84	16.09	-1.73	0.00845
Nitab4.5_0006217g0020	Reverse transcriptase zinc-binding domain	6.63	21.91	-1.72	0.03011
Nitab4.5_0007605g0020	DNA-binding WRKY	11.45	37.84	-1.72	0.01631
Nitab4.5_0001552g0120	P-loop containing nucleoside triphosphate hydrolase	5.45	18.01	-1.72	0.00181
Nitab4.5_0006179g0020	F-box domain, Phloem protein 2-like	2.37	7.82	-1.72	0.00161
Nitab4.5_0003395g0040		5.03	16.56	-1.72	0.00002
Nitab4.5_0009635g0010	Oxoglutarate/iron-dependent dioxygenase, Isopenicillin N synthase-like, Nor	15.27	50.23	-1.72	0.00000
Nitab4.5_0011752g0010		38.01	123.92	-1.70	0.00005
Nitab4.5_0000786g0230	Phosphate-induced protein 1	4.33	14.10	-1.70	0.00276
Nitab4.5_0000278g0040	Alpha crystallin/Hsp20 domain, HSP20-like chaperone	8.04	26.14	-1.70	0.00354
Nitab4.5_0002780g0140	Yippee/Mis18	84.10	273.35	-1.70	0.00240
Nitab4.5_0001134g0020	EF-hand domain, EF-Hand 1, calcium-binding site, EF-hand domain pair	13.33	43.31	-1.70	0.00527
Nitab4.5_0003413g0040	Expansin, cellulose-binding-like domain, Expansin/Lol pI, Expansin, RlpA-like	3.47	11.23	-1.69	0.00046
Nitab4.5_0000829g0200	Thioredoxin-like fold, Glutaredoxin, Glutaredoxin-like, plant II	26.10	84.37	-1.69	0.00016
Nitab4.5_0007225g0020		6.54	21.05	-1.69	0.00083
Nitab4.5_0008962g0010	DNA-binding WRKY	7.12	22.84	-1.68	0.00013
Nitab4.5_0000459g0060	Heat shock transcription factor family, Winged helix-turn-helix DNA-binding c	2.25	7.16	-1.67	0.00561
Nitab4.5_0001105g0180	Zinc finger, RING-type, Zinc finger, RING/FYVE/PHD-type	12.69	40.15	-1.66	0.00009
Nitab4.5_0000697g0070	DnaJ domain, DnaJ domain, conserved site	35.93	112.76	-1.65	0.00000
Nitab4.5_0008683g0010	HAD-superfamily hydrolase, subfamily IIB, HAD-like domain, Trehalose-phos	4.20	13.14	-1.64	0.00006
Nitab4.5_0010567g0010	Nucleic acid-binding, OB-fold, Cold-shock protein, DNA-binding, Zinc finger,	2.05	6.36	-1.63	0.00307
Nitab4.5_0004937g0060	Cytochrome P450, Cytochrome P450, conserved site, Cytochrome P450, E-	4.17	12.88	-1.63	0.00370
Nitab4.5_0005466g0030		4.01	12.39	-1.63	0.00090
Nitab4.5_0000008g0860	Chloramphenicol acetyltransferase-like domain, Transferase	49.96	153.33	-1.62	0.00000

Nitab4.5_0025878g0010	Nucleoside phosphorylase domain, Nucleoside phosphorylase	193.49	592.85	-1.62	0.00004
Nitab4.5_0001867g0040		7.53	22.87	-1.60	0.02461
Nitab4.5_0009046g0010	Cysteine-rich transmembrane CYSTM domain	15.05	45.71	-1.60	0.00000
Nitab4.5_0003408g0110	Glycosyl hydrolase, five-bladed beta-propellor domain, Exostosin-like, EXTL:	5.60	16.89	-1.59	0.01700
Nitab4.5_0007598g0030	Alpha crystallin/Hsp20 domain, HSP20-like chaperone	3.52	10.60	-1.59	0.00430
Nitab4.5_0011213g0010	EF-hand domain, EF-hand domain pair, EF-Hand 1, calcium-binding site	30.78	92.62	-1.59	0.00022
Nitab4.5_0007879g0040		3.56	10.72	-1.59	0.00282
Nitab4.5_0000586g0010	AP2/ERF domain, DNA-binding domain	5.20	15.59	-1.58	0.00002
Nitab4.5_0000692g0050		6.21	18.58	-1.58	0.00208
Nitab4.5_0003757g0050	Galactose oxidase/kelch, beta-propeller, F-box domain, Kelch-type beta prop	6.98	20.87	-1.58	0.00000
Nitab4.5_0002066g0110	Domain of unknown function DUF3700	15.85	47.42	-1.58	0.00000
Nitab4.5_0002569g0100	Leucine-rich repeat, Leucine rich repeat 4	5.58	16.70	-1.58	0.00028
Nitab4.5_0009217g0020	Ammonium transporter, conserved site, Ammonium transporter, Ammonium	6.00	17.91	-1.58	0.00809
Nitab4.5_0005904g0010	Terpene synthase, N-terminal domain, Terpenoid cyclases/protein prenyltrar	8.32	24.82	-1.58	0.02336
Nitab4.5_0000444g0190	Ammonium transporter, Ammonium transporter, conserved site, Ammonium	4.71	14.05	-1.58	0.01311
Nitab4.5_0011148g0010	DNA-binding WRKY, Zn-cluster domain	7.30	21.68	-1.57	0.00388
Nitab4.5_0003767g0050	Zinc finger, RING/FYVE/PHD-type, Zinc finger, RING-type	8.54	25.23	-1.56	0.00147
Nitab4.5_0002545g0080		8.97	26.44	-1.56	0.00802
Nitab4.5_0001478g0160		8.51	24.97	-1.55	0.00235
Nitab4.5_0002426g0020		21.94	64.28	-1.55	0.00082
Nitab4.5_0027211g0010	Leucine zipper, homeobox-associated, Homeobox, conserved site, HD-ZIP p	4.08	11.88	-1.54	0.00098
Nitab4.5_0002820g0100	Late embryogenesis abundant protein, LEA-5	64.69	187.59	-1.54	0.00000
Nitab4.5_0001742g0030	Cytochrome P450, Cytochrome P450, conserved site, Cytochrome P450, E-	3.39	9.81	-1.53	0.00481
Nitab4.5_0003594g0010	Concanavalin A-like lectin/glucanase, subgroup, Xyloglucan endotransglucos	20.66	59.62	-1.53	0.00000
Nitab4.5_0003641g0020	Armadillo-type fold, Armadillo-like helical	3.33	9.61	-1.53	0.00118
Nitab4.5_0007739g0010	AP2/ERF domain, DNA-binding domain	12.36	35.63	-1.53	0.00210
Nitab4.5_0001024g0060	EF-Hand 1, calcium-binding site, EF-hand domain, EF-hand domain pair	44.61	127.64	-1.52	0.00072
Nitab4.5_0007730g0010	AP2/ERF domain, DNA-binding domain	8.06	23.00	-1.51	0.00035
Nitab4.5_0024190g0010	Protein of unknown function DUF284, transmembrane eukaryotic	9.12	26.01	-1.51	0.00293
Nitab4.5_0000008g0870	Transferase, Chloramphenicol acetyltransferase-like domain	33.74	96.07	-1.51	0.00000
Nitab4.5_0000733g0120		6.53	18.59	-1.51	0.00678
Nitab4.5_0004188g0060	Mitochondrial import receptor subunit TOM7	19.94	56.71	-1.51	0.01606
Nitab4.5_0000380g0160	Ribonuclease H-like domain, Ribonuclease CAF1	13.42	38.18	-1.51	0.00172
Nitab4.5_0000570g0120	Heat shock factor binding 1	12.03	34.20	-1.51	0.02685
Nitab4.5_0000224g0060	Cation-transporting P-type ATPase, P-type ATPase, transmembrane domai	4.23	12.02	-1.51	0.00165
Nitab4.5_0007575g0040	RecF/RecN/SMC, N-terminal, P-loop containing nucleoside triphosphate hyd	6.90	19.60	-1.51	0.01525
Nitab4.5_0000164g0180	Zinc finger, C2H2, Zinc finger C2H2-type/integrase DNA-binding domain, Zir	18.44	52.38	-1.51	0.00118
Nitab4.5_0010493g0010	AP2/ERF domain, DNA-binding domain	9.97	28.23	-1.50	0.04739
Nitab4.5_0001021g0070		18.13	51.34	-1.50	0.00031

Nitab4.5_0005448g0040		9.28	26.18	-1.50	0.01152
Nitab4.5_0000239g0050	START-like domain, Polyketide cyclase/dehydrase	5.67	15.99	-1.50	0.00645
Nitab4.5_0003481g0110	VQ	13.80	38.85	-1.49	0.01979
Nitab4.5_0010236g0020	Late embryogenesis abundant protein, LEA-5	12.19	34.33	-1.49	0.04339
Nitab4.5_0000731g0010	F-box domain	3.20	8.96	-1.48	0.00461
Nitab4.5_0004441g0010	Heat shock factor (HSF)-type, DNA-binding, Heat shock transcription factor f	5.27	14.72	-1.48	0.00056
Nitab4.5_0001079g0120		57.25	159.50	-1.48	0.00024
Nitab4.5_0001949g0040		15.81	43.96	-1.48	0.00178
Nitab4.5_0000299g0200		7.72	21.46	-1.47	0.00034
Nitab4.5_0001731g0020	AP2/ERF domain, DNA-binding domain	13.43	37.31	-1.47	0.00485
Nitab4.5_0000600g0080	AP2/ERF domain, DNA-binding domain	4.47	12.35	-1.47	0.00380
Nitab4.5_0006355g0030	F-box domain, Phloem protein 2-like	8.75	24.19	-1.47	0.00040
Nitab4.5_0007624g0050		21.94	60.47	-1.46	0.00000
Nitab4.5_0002344g0080	C2 domain	7.28	20.07	-1.46	0.00001
Nitab4.5_0000259g0120	TCP-1-like chaperonin intermediate domain, Chaperonin Cpn60/TCP-1	22.24	61.19	-1.46	0.00036
Nitab4.5_0007585g0030	Serine/threonine- / dual specificity protein kinase, catalytic domain, Protein k	5.18	14.23	-1.46	0.00042
Nitab4.5_0007252g0020	Lipoxygenase, Lipoxygenase, C-terminal, Lipase/lipooxygenase, PLAT/LH2,	10.86	29.80	-1.46	0.00126
Nitab4.5_0001031g0010		7.32	20.08	-1.46	0.00002
Nitab4.5_0003603g0040	RlpA-like double-psi beta-barrel domain, Barwin-like endoglucanase, Expans	4.84	13.27	-1.45	0.00029
Nitab4.5_0000021g0470	P-loop containing nucleoside triphosphate hydrolase, Small GTPase superfa	8.19	22.37	-1.45	0.00021
Nitab4.5_0001586g0220	Protein of unknown function DUF947	3.67	10.03	-1.45	0.00349
Nitab4.5_0014067g0010		16.53	45.07	-1.45	0.00186
Nitab4.5_0007701g0010	Concanavalin A-like lectin/glucanase, subgroup, Glycoside hydrolase, family	15.84	43.10	-1.44	0.00000
Nitab4.5_0007710g0010	Protein kinase-like domain, Protein kinase, ATP binding site, Serine/threonin	4.28	11.65	-1.44	0.00098
Nitab4.5_0007088g0020	LysM domain	14.24	38.57	-1.44	0.00001
Nitab4.5_0003052g0010		41.28	111.40	-1.43	0.00044
Nitab4.5_0000037g0360	Systemic acquired resistance protein SAR	664.99	1793.30	-1.43	0.04945
Nitab4.5_0001525g0070	SAM dependent carboxyl methyltransferase	4.01	10.82	-1.43	0.00111
Nitab4.5_0006461g0040	Protein of unknown function DUF1005	3.71	10.00	-1.43	0.00349
Nitab4.5_0000584g0040		32.01	86.17	-1.43	0.01376
Nitab4.5_0012187g0010	EF-hand domain, EF-Hand 1, calcium-binding site, EF-hand domain pair	20.91	56.10	-1.42	0.00000
Nitab4.5_0004523g0050	Glutathione S-transferase, C-terminal-like, Glutathione S-transferase, C-term	54.80	146.98	-1.42	0.00236
Nitab4.5_0000256g0420		5.41	14.49	-1.42	0.01047
Nitab4.5_0002039g0050		17.40	46.57	-1.42	0.00817
Nitab4.5_0026409g0010	MORN motif	7.67	20.49	-1.42	0.00001
Nitab4.5_0009367g0020		40.63	107.65	-1.41	0.00019
Nitab4.5_0002329g0160		6.10	16.13	-1.40	0.00058
Nitab4.5_0000926g0150	Concanavalin A-like lectin/glucanase, subgroup, Concanavalin A-like lectin/g	37.13	98.22	-1.40	0.00000
Nitab4.5_0002151g0020	Galactose oxidase, beta-propeller, Kelch repeat type 1	11.05	29.16	-1.40	0.00000

Nitab4.5_0003233g0040	F-box domain, F-box associated interaction domain	10.85	28.61	-1.40	0.00290
Nitab4.5_0008969g0020	Pyridoxal phosphate-dependent transferase, major region, subdomain 2, Pyr	10.70	28.19	-1.40	0.00000
Nitab4.5_0012382g0010	Fumarase/histidase, N-terminal, Phenylalanine ammonia-lyase, shielding do	9.35	24.62	-1.40	0.00231
Nitab4.5_0000600g0060	AP2/ERF domain, DNA-binding domain	6.13	16.13	-1.40	0.00768
Nitab4.5_0009567g0010	RlpA-like double-psi beta-barrel domain, Expansin, cellulose-binding-like dor	3.79	9.93	-1.39	0.00478
Nitab4.5_0000068g0290	NB-ARC, Leucine-rich repeat, Disease resistance protein, P-loop containing	10.53	27.45	-1.38	0.00048
Nitab4.5_0003498g0020	Protein of unknown function DUF966, Uncharacterised conserved protein UC	4.20	10.94	-1.38	0.00094
Nitab4.5_0009672g0060		11.76	30.64	-1.38	0.00613
Nitab4.5_0000082g0370	EF-hand domain pair, EF-hand domain, EF-Hand 1, calcium-binding site	22.76	59.26	-1.38	0.00000
Nitab4.5_0005385g0020	Zinc finger, C2H2, Zinc finger, C2H2-like, Zinc finger C2H2-type/integrase D	23.68	61.64	-1.38	0.00951
Nitab4.5_0000079g0240		25.79	66.87	-1.37	0.00537
Nitab4.5_0003995g0010	MYB-CC type transcription factor, LHEQLE-containing domain	7.65	19.85	-1.37	0.00001
Nitab4.5_0001965g0080	EF-Hand 1, calcium-binding site, EF-hand domain, EF-hand domain pair	3.75	9.71	-1.37	0.00581
Nitab4.5_0006895g0040		11.39	29.44	-1.37	0.03258
Nitab4.5_0000043g0340	Lipoxygenase, C-terminal, Lipoxygenase, conserved site, PLAT/LH2 domain	8.37	21.62	-1.37	0.01026
Nitab4.5_0000241g0150	Zinc finger, RING-type, Zinc finger, RING/FYVE/PHD-type	34.14	88.08	-1.37	0.00054
Nitab4.5_0000034g0110	Peptidoglycan binding-like, Metallopeptidase, catalytic domain, Peptidase M	6.65	17.05	-1.36	0.00965
Nitab4.5_0000593g0240	Exostosin-like, Glycosyl hydrolase, five-bladed beta-propellor domain, EXTL	23.78	60.84	-1.36	0.02427
Nitab4.5_0000041g0020	Dormancyauxin associated	68.76	175.85	-1.35	0.00000
Nitab4.5_0013033g0010	Protein kinase domain, Serine/threonine-protein kinase, active site, NAF don	7.60	19.41	-1.35	0.00131
Nitab4.5_0000525g0080	Protein kinase domain, Protein kinase-like domain	80.92	206.53	-1.35	0.00848
Nitab4.5_0000933g0030	HSP20-like chaperone, Alpha crystallin/Hsp20 domain	4.84	12.35	-1.35	0.00227
Nitab4.5_0000695g0100	Zinc finger, RING-type, Zinc finger, RING/FYVE/PHD-type	15.48	39.43	-1.35	0.00257
Nitab4.5_0008962g0050	DnaJ domain	15.20	38.68	-1.35	0.00221
Nitab4.5_0013609g0010	Fatty acid hydroxylase, Cation-transporting P-type ATPase, C-terminal	26.27	66.83	-1.35	0.00108
Nitab4.5_0000020g0340		5.10	12.95	-1.35	0.01097
Nitab4.5_0000684g0170	DNA-binding WRKY	19.76	50.20	-1.35	0.01916
Nitab4.5_0003716g0110		6.37	16.17	-1.34	0.00013
Nitab4.5_0000319g0090		20.14	51.00	-1.34	0.03433
Nitab4.5_0000015g0100	Homeobox domain, Homeodomain-like, Leucine zipper, homeobox-associate	14.30	36.18	-1.34	0.00000
Nitab4.5_0002211g0060	AP2/ERF domain, DNA-binding domain	62.30	157.54	-1.34	0.00000
Nitab4.5_0012695g0020		4.40	11.12	-1.34	0.00311
Nitab4.5_0000022g0300	Protein kinase domain, Serine/threonine-protein kinase, active site, Protein k	18.35	46.34	-1.34	0.00487
Nitab4.5_0010414g0050		53.16	134.00	-1.33	0.00000
Nitab4.5_0000980g0280		4.97	12.51	-1.33	0.00143
Nitab4.5_0007798g0010	WD40-repeat-containing domain, WD40 repeat, WD40/YVTN repeat-like-cor	5.40	13.56	-1.33	0.00079
Nitab4.5_0000857g0030		13.21	33.01	-1.32	0.00005
Nitab4.5_0009581g0020	Small GTPase superfamily, P-loop containing nucleoside triphosphate hydro	6.28	15.64	-1.32	0.01067
Nitab4.5_0000040g0550	Domain of unknown function, DUF971	9.77	24.31	-1.32	0.00359

Nitab4.5_0022963g0010		10.40	25.83	-1.31	0.00005
Nitab4.5_0000590g0020	MORN motif	7.61	18.88	-1.31	0.00005
Nitab4.5_0004411g0010	Zinc finger, RING-type, conserved site, Zinc finger, RING/FYVE/PHD-type, Z	7.99	19.79	-1.31	0.00007
Nitab4.5_0001349g0010	EF-hand domain, EF-hand domain pair, EF-Hand 1, calcium-binding site	5.15	12.76	-1.31	0.00104
Nitab4.5_0003051g0020		8.66	21.34	-1.30	0.01330
Nitab4.5_0001664g0080		10.59	25.92	-1.29	0.00016
Nitab4.5_0002760g0050	Basic-leucine zipper domain	5.66	13.83	-1.29	0.00057
Nitab4.5_0002236g0040	AP2/ERF domain, DNA-binding domain	43.27	105.65	-1.29	0.00000
Nitab4.5_0003328g0120	Heat shock protein Hsp90, N-terminal, Histidine kinase-like ATPase, ATP-bi	8.45	20.60	-1.29	0.02385
Nitab4.5_0005040g0040		21.01	51.19	-1.28	0.04243
Nitab4.5_0004030g0050	Chloramphenicol acetyltransferase-like domain, Transferase	15.37	37.34	-1.28	0.00023
Nitab4.5_0007439g0020	Rhodanese-like domain	29.43	71.48	-1.28	0.00018
Nitab4.5_0019152g0010	Pentatricopeptide repeat	39.73	96.18	-1.28	0.00095
Nitab4.5_0000062g0220	Drug/metabolite transporter	4.37	10.56	-1.27	0.00258
Nitab4.5_0002796g0050	Peptidase S10, serine carboxypeptidase	54.58	131.90	-1.27	0.00374
Nitab4.5_0004063g0040		13.73	33.12	-1.27	0.00000
Nitab4.5_0002169g0070	Zinc finger, RING-type, Zinc finger, RING/FYVE/PHD-type, Zinc finger, RINC	28.44	68.59	-1.27	0.00030
Nitab4.5_0012375g0020	Thioredoxin-like fold	26.05	62.69	-1.27	0.00000
Nitab4.5_0000743g0010	Protein kinase domain, Serine/threonine- / dual specificity protein kinase, cat	9.27	22.26	-1.26	0.01672
Nitab4.5_0001582g0030		7.62	18.25	-1.26	0.00722
Nitab4.5_0000190g0190	Calmodulin binding protein-like	6.09	14.54	-1.25	0.01291
Nitab4.5_0000082g0410		7.83	18.65	-1.25	0.00155
Nitab4.5_0004330g0020	Non-haem dioxygenase N-terminal domain, Oxoglutarate/iron-dependent dic	64.95	154.52	-1.25	0.03092
Nitab4.5_0000319g0290	Homeodomain-like, Homeobox domain, Homeobox, conserved site, Helix-tu	7.74	18.35	-1.25	0.00016
Nitab4.5_0000516g0140		40.32	95.34	-1.24	0.00397
Nitab4.5_0005738g0030		8.65	20.46	-1.24	0.00044
Nitab4.5_0003854g0030	SKP1 component, SKP1 component, dimerisation, BTB/POZ fold	12.99	30.65	-1.24	0.00032
Nitab4.5_0002682g0040		20.50	48.25	-1.24	0.00064
Nitab4.5_0002269g0030	Fatty acid hydroxylase	20.34	47.87	-1.23	0.00011
Nitab4.5_0000914g0180	Protein of unknown function DUF284, transmembrane eukaryotic	8.27	19.45	-1.23	0.02240
Nitab4.5_0001190g0040	Heat shock protein 70 family, Heat shock protein 70, conserved site	31.51	74.09	-1.23	0.00000
Nitab4.5_0001786g0040	Homeodomain-like, Myb domain, SANT/Myb domain, SANT domain, Myb dc	6.31	14.81	-1.23	0.00079
Nitab4.5_0006014g0030	Heavy metal-associated domain, HMA	38.78	90.89	-1.23	0.00370
Nitab4.5_0004847g0060		24.77	58.05	-1.23	0.00234
Nitab4.5_0000168g0050		9.01	21.10	-1.23	0.01716
Nitab4.5_0003902g0040	Pseudouridine synthase/archaeosine transglycosylase, PUA-like domain, tR	10.98	25.70	-1.23	0.00057
Nitab4.5_0001610g0040	NAC domain	7.42	17.36	-1.23	0.00264
Nitab4.5_0009186g0020		5.95	13.87	-1.22	0.00218
Nitab4.5_0004246g0040	RWP-RK domain, ICP0-binding domain of Ubiquitin-specific protease 7, AP2	17.66	41.12	-1.22	0.00000

Nitab4.5_0008315g0010		18.37	42.73	-1.22	0.00001
Nitab4.5_0002578g0010	Myb domain, Homeodomain-like, SANT/Myb domain	20.15	46.80	-1.22	0.00005
Nitab4.5_0002116g0020		24.75	57.37	-1.21	0.02207
Nitab4.5_0000978g0060	Thioredoxin-like fold	10.82	25.03	-1.21	0.00002
Nitab4.5_0005669g0020		5.83	13.45	-1.21	0.00366
Nitab4.5_0012065g0030	DnaJ domain	10.19	23.50	-1.21	0.00260
Nitab4.5_0001373g0030	Homeodomain-like, Myb domain, SANT/Myb domain	14.84	34.12	-1.20	0.00001
Nitab4.5_0000058g0060	Polyketide cyclase/dehydrase, START-like domain	11.28	25.89	-1.20	0.00045
Nitab4.5_0002683g0010		11.00	25.20	-1.20	0.00009
Nitab4.5_0005289g0020		44.20	101.20	-1.20	0.00026
Nitab4.5_0003887g0040		7.23	16.53	-1.19	0.00133
Nitab4.5_0000207g0410	EF-hand domain pair, EF-Hand 1, calcium-binding site, EF-hand domain	30.11	68.79	-1.19	0.00542
Nitab4.5_0016580g0020		12.74	29.05	-1.19	0.00113
Nitab4.5_0001302g0030		6.91	15.75	-1.19	0.01009
Nitab4.5_0000308g0100	AP complex, mu/sigma subunit, Longin-like domain, Adaptor protein complex	12.29	28.00	-1.19	0.00219
Nitab4.5_0005031g0050	EF-hand domain, EF-hand domain pair, EF-Hand 1, calcium-binding site	254.26	578.68	-1.19	0.00000
Nitab4.5_0000132g0530	UDP-glucuronosyl/UDP-glucosyltransferase	12.75	28.94	-1.18	0.00010
Nitab4.5_0006193g0010		17.60	39.85	-1.18	0.00382
Nitab4.5_0007800g0010	Dormancyauxin associated	352.77	794.92	-1.17	0.00000
Nitab4.5_0006669g0020	NAD(P)-binding domain, Glucose/ribitol dehydrogenase, Short-chain dehydr	9.76	21.92	-1.17	0.00025
Nitab4.5_0002056g0070	Phloem protein 2-like	32.91	73.91	-1.17	0.00000
Nitab4.5_0010196g0040	Mitochondrial substrate/solute carrier, Mitochondrial carrier domain, Mitochon	41.73	93.57	-1.17	0.00339
Nitab4.5_0011920g0010	Cupredoxin, Plastocyanin-like	18.28	40.98	-1.16	0.00199
Nitab4.5_0011192g0020		16.86	37.72	-1.16	0.00045
Nitab4.5_0002016g0030	Heat shock factor (HSF)-type, DNA-binding, Heat shock transcription factor f	11.40	25.49	-1.16	0.01352
Nitab4.5_0000063g0170		7.40	16.55	-1.16	0.00505
Nitab4.5_0001204g0100	NAC domain	20.94	46.69	-1.16	0.00000
Nitab4.5_0000350g0190		49.73	110.86	-1.16	0.00002
Nitab4.5_0000917g0020	EF-hand domain pair, EF-hand domain, EF-Hand 1, calcium-binding site	21.57	47.92	-1.15	0.00004
Nitab4.5_0003501g0050	Immunoglobulin-like fold, Late embryogenesis abundant protein, LEA-14	8.57	18.99	-1.15	0.00041
Nitab4.5_0013137g0020	Zinc finger, CCCH-type, Ankyrin repeat, Ankyrin repeat-containing domain	49.90	110.39	-1.15	0.00002
Nitab4.5_0010321g0020	Tetratricopeptide-like helical, Tetratricopeptide repeat-containing domain, Te	66.20	146.22	-1.14	0.01950
Nitab4.5_0003779g0050	G-box binding, MFMR	7.59	16.76	-1.14	0.00102
Nitab4.5_0005929g0020		19.40	42.83	-1.14	0.00001
Nitab4.5_0001047g0070	Myb domain, plants, Myb domain, Homeodomain-like, SANT domain, SANT/	10.95	24.16	-1.14	0.00038
Nitab4.5_0000744g0140		28.06	61.83	-1.14	0.00006
Nitab4.5_0007477g0010	Transcription factor IIS, N-terminal, Transcription elongation factor, TFIIS/CF	7.85	17.27	-1.14	0.00043
Nitab4.5_0010472g0030	UDP-glucose 4-epimerase C-terminal domain, UDP-glucose 4-epimerase Ga	10.74	23.59	-1.14	0.00879
Nitab4.5_0001157g0010	Acyl-CoA N-acyltransferase, GNAT domain	11.20	24.60	-1.14	0.01014

Nitab4.5_0003437g0010	Protein of unknown function DUF597	23.31	51.14	-1.13	0.00046
Nitab4.5_0007505g0040	Transcription factor, K-box	11.23	24.63	-1.13	0.01730
Nitab4.5_0001608g0020	PMR5 N-terminal domain, PC-Esterase	47.56	104.08	-1.13	0.00000
Nitab4.5_0004319g0010	Phosphoribosyltransferase domain	11.01	24.10	-1.13	0.00232
Nitab4.5_0000716g0240		10.48	22.87	-1.13	0.00965
Nitab4.5_0009884g0020	Heat shock protein 70 family, Heat shock protein 70, conserved site	379.07	826.87	-1.13	0.00000
Nitab4.5_0000174g0290	Transcription factor GRAS	10.14	22.06	-1.12	0.00013
Nitab4.5_0000376g0140	Sodium/calcium exchanger membrane region, EF-hand domain pair, EF-Har	13.15	28.63	-1.12	0.00257
Nitab4.5_0001406g0010	Glycosyl transferase, family 20, Trehalose-phosphatase, HAD-like domain, H	7.64	16.61	-1.12	0.00076
Nitab4.5_0000046g0020	DNA-binding WRKY	20.93	45.53	-1.12	0.04509
Nitab4.5_0001863g0230	Domain of unknown function DUF23	6.78	14.75	-1.12	0.00338
Nitab4.5_0003602g0040	Dyskerin-like, Pseudouridine synthase/archaeosine transglycosylase, PUA-li	7.83	17.00	-1.12	0.00309
Nitab4.5_0002719g0120	Alpha/beta hydrolase fold-3	11.03	23.90	-1.12	0.01288
Nitab4.5_0009622g0010	Protein phosphatase 2C, manganese/magnesium aspartate binding site, Pro	10.82	23.44	-1.11	0.00020
Nitab4.5_0002700g0200		44.47	96.15	-1.11	0.00472
Nitab4.5_0005046g0010	Protein of unknown function DUF1005	6.98	15.08	-1.11	0.00138
Nitab4.5_0000550g0080		35.42	76.50	-1.11	0.00266
Nitab4.5_0008566g0080	Protein of unknown function DUF597	31.46	67.88	-1.11	0.00011
Nitab4.5_0001266g0060	Annexin repeat, Annexin repeat, conserved site, Annexin, plant, Annexin	10.57	22.74	-1.11	0.00016
Nitab4.5_0006895g0010		35.25	75.54	-1.10	0.00007
Nitab4.5_0001622g0050	Heat shock protein Hsp90 family, Histidine kinase-like ATPase, ATP-binding	63.88	136.75	-1.10	0.00093
Nitab4.5_0004436g0040	Galactose oxidase, beta-propeller, Kelch repeat type 1	17.61	37.65	-1.10	0.00000
Nitab4.5_0004872g0010	Membrane-anchored ubiquitin-fold protein, HCG-1, Ubiquitin supergroup	14.54	30.94	-1.09	0.00245
Nitab4.5_0001079g0100	F-box domain, Galactose oxidase, beta-propeller, Kelch repeat type 1	148.44	315.31	-1.09	0.00016
Nitab4.5_0000996g0050	Signal transduction response regulator, receiver domain, CheY-like superfan	15.90	33.60	-1.08	0.00000
Nitab4.5_0000686g0110	EF-hand domain pair, EF-Hand 1, calcium-binding site, EF-hand domain	13.03	27.40	-1.07	0.00007
Nitab4.5_0003561g0030		18.33	38.42	-1.07	0.00026
Nitab4.5_0007732g0020	Formiminotransferase, N-terminal subdomain	40.76	85.28	-1.07	0.03100
Nitab4.5_0006978g0010	Transcription factor GRAS	8.02	16.77	-1.06	0.00873
Nitab4.5_0004384g0050	EF-hand domain pair, EF-hand domain, EF-Hand 1, calcium-binding site	39.98	83.49	-1.06	0.00682
Nitab4.5_0002205g0020	ABC transporter-like, P-loop containing nucleoside triphosphate hydrolase, F	26.86	56.08	-1.06	0.00777
Nitab4.5_0000365g0050	SANT/Myb domain, Homeodomain-like, Myb domain	7.73	16.10	-1.06	0.00219
Nitab4.5_0005310g0010	6-phosphogluconate dehydrogenase, C-terminal-like, Fibritin/6-phosphogluc	14.86	30.96	-1.06	0.00493
Nitab4.5_0012646g0010	HAD-like domain, Trehalose-phosphatase, Glycosyl transferase, family 20, H	9.29	19.33	-1.06	0.00209
Nitab4.5_0000976g0340	Cytochrome c oxidase assembly protein PET191	21.04	43.75	-1.06	0.00240
Nitab4.5_0000566g0200		103.10	214.23	-1.06	0.02138
Nitab4.5_0004778g0020	DREPP family	127.87	265.14	-1.05	0.00000
Nitab4.5_0001902g0020	EF-hand domain, EF-Hand 1, calcium-binding site, EF-hand domain pair	204.53	423.74	-1.05	0.00009
Nitab4.5_0003626g0010		248.11	513.89	-1.05	0.00003

Nitab4.5_0008414g0050		780.08	1614.52	-1.05	0.00017
Nitab4.5_0000570g0190	Late embryogenesis abundant protein, LEA-5	62.69	129.43	-1.05	0.00237
Nitab4.5_0000271g0040		7.13	14.72	-1.05	0.00396
Nitab4.5_0004813g0010	VQ	8.73	17.96	-1.04	0.00538
Nitab4.5_0007373g0020	Protein kinase, ATP binding site, Concanavalin A-like lectin/glucanase, subg	8.05	16.56	-1.04	0.00287
Nitab4.5_0003773g0020	Phloem protein 2-like	7.16	14.70	-1.04	0.00450
Nitab4.5_0000906g0020	Lipase, class 3	10.63	21.80	-1.04	0.00044
Nitab4.5_0008981g0010	XS domain	10.93	22.39	-1.03	0.00034
Nitab4.5_0005003g0010	Mitochondrial inner membrane translocase subunit Tim17/Tim22/Tim23/perc	12.58	25.75	-1.03	0.00025
Nitab4.5_0000091g0420	Oxoglutarate/iron-dependent dioxygenase, Isopenicillin N synthase-like	91.71	187.36	-1.03	0.00017
Nitab4.5_0002427g0040		8.04	16.40	-1.03	0.00212
Nitab4.5_0007755g0020	Yippee/Mis18	19.99	40.79	-1.03	0.02956
Nitab4.5_0000143g0170		368.28	751.43	-1.03	0.00000
Nitab4.5_0000802g0150	Hs1pro-1, C-terminal, Hs1pro-1, N-terminal	26.90	54.70	-1.02	0.00162
Nitab4.5_0004860g0010	SKP1 component, POZ domain, E3 ubiquitin ligase, SCF complex, Skp subu	10.46	21.25	-1.02	0.00275
Nitab4.5_0001102g0070		16.53	33.56	-1.02	0.00055
Nitab4.5_0003041g0010	PHF5-like	7.94	16.13	-1.02	0.00231
Nitab4.5_0008421g0020	Derlin	9.61	19.51	-1.02	0.01327
Nitab4.5_0003368g0020	EF-hand domain pair, EF-hand domain, EF-Hand 1, calcium-binding site	40.80	82.81	-1.02	0.00618
Nitab4.5_0003883g0030	Dehydrin	18.07	36.65	-1.02	0.00008
Nitab4.5_0011134g0010	RNA recognition motif domain, Nucleotide-binding, alpha-beta plait	33.41	67.48	-1.01	0.00055
Nitab4.5_0004955g0020	Homeodomain-like, Homeobox domain	17.59	35.46	-1.01	0.00142
Nitab4.5_0003540g0010	Basic-leucine zipper domain	59.36	119.63	-1.01	0.00019
Nitab4.5_0001566g0030		724.02	1458.61	-1.01	0.00263
Nitab4.5_0009514g0070	Protein of unknown function DUF597	80.56	162.28	-1.01	0.00012
Nitab4.5_0003541g0050	Transcriptional activator, plants	13.98	28.13	-1.01	0.00158
Nitab4.5_0003201g0010		19.91	40.06	-1.01	0.00106
Nitab4.5_0005716g0010		34.02	68.42	-1.01	0.01834
Nitab4.5_0001761g0250	Ribosomal protein S2, bacteria/mitochondria/plastid, Ribosomal protein S2, f	35.80	71.99	-1.01	0.00192
Nitab4.5_0001764g0140		10.22	20.50	-1.00	0.00571
Nitab4.5_0004384g0060	EF-hand domain pair, EF-hand domain, EF-Hand 1, calcium-binding site	21.92	43.97	-1.00	0.04028
Nitab4.5_0002405g0130	Tim10/DDP family zinc finger, Mitochondrial import inner membrane transloc	12.98	26.03	-1.00	0.00239
Nitab4.5_0009884g0010	Glutamate dehydrogenase, NAD-specific	42.26	84.55	-1.00	0.00001
Nitab4.5_0000015g0180	Rhodanese-like domain	75.67	37.83	1.00	0.00000
Nitab4.5_0005472g0080		67.26	33.62	1.00	0.01376
Nitab4.5_0007992g0040	Tetratricopeptide repeat-containing domain, Tetratricopeptide-like helical	126.27	63.02	1.00	0.00010
Nitab4.5_0026691g0010	START-like domain, Polyketide cyclase/dehydrase	19.07	9.51	1.00	0.00180
Nitab4.5_0000408g0150	HSP40/DnaJ peptide-binding, DnaJ domain, conserved site, DnaJ domain, C	36.20	18.05	1.00	0.00002
Nitab4.5_0001044g0040	Photosystem II PsbW, class 2	55.64	27.70	1.01	0.00001

Nitab4.5_0001312g0020	Photosystem I PsaF, reaction centre subunit III	293.53	145.76	1.01	0.00000
Nitab4.5_0000136g0100	Fructose-bisphosphate aldolase, class-I, Aldolase-type TIM barrel	117.23	58.17	1.01	0.01362
Nitab4.5_0008435g0010	NAD-dependent epimerase/dehydratase, NAD(P)-binding domain	60.11	29.82	1.01	0.00000
Nitab4.5_0002320g0020	Chloramphenicol acetyltransferase-like domain, Transferase	61.13	30.27	1.01	0.00000
Nitab4.5_0013647g0010	Thioredoxin, conserved site, Thioredoxin-like fold, Thioredoxin domain, Thior	60.42	29.86	1.02	0.00000
Nitab4.5_0014046g0010	HAD-like domain, Phosphatase PHOSPHO-type, HAD-superfamily hydrolas	59.94	29.57	1.02	0.00002
Nitab4.5_0003318g0060		37.06	18.26	1.02	0.00025
Nitab4.5_0003110g0130	DNA-binding domain, AP2/ERF domain	18.75	9.22	1.02	0.00079
Nitab4.5_0001998g0070	PDZ domain, Interphotoreceptor retinol-binding, C-terminal-processing pepti	23.36	11.47	1.03	0.00011
Nitab4.5_0001477g0170	Malate dehydrogenase, type 1, L-lactate/malate dehydrogenase, Lactate/ma	220.81	108.35	1.03	0.00000
Nitab4.5_0002101g0060	Aldehyde dehydrogenase, conserved site, Aldehyde dehydrogenase domain	21.27	10.43	1.03	0.00030
Nitab4.5_0000305g0210	Cyanobacterial aminoacyl-tRNA synthetase, CAAD domain	212.59	104.13	1.03	0.00000
Nitab4.5_0000263g0090	Myc-type, basic helix-loop-helix (bHLH) domain	17.91	8.77	1.03	0.00111
Nitab4.5_0012161g0020		33.18	16.24	1.03	0.00005
Nitab4.5_0000010g0210	Tetratricopeptide repeat-containing domain, Tetratricopeptide-like helical	101.82	49.85	1.03	0.00002
Nitab4.5_0000705g0030	Chlorophyll a/b binding protein domain	100.35	49.09	1.03	0.00000
Nitab4.5_0002946g0010	Photosystem I PsaD	96.65	47.23	1.03	0.00000
Nitab4.5_0008471g0030	Alcohol dehydrogenase superfamily, zinc-type, GroES (chaperonin 10)-like,	96.56	47.18	1.03	0.00006
Nitab4.5_0005361g0020		49.46	24.16	1.03	0.01830
Nitab4.5_0004658g0050	2-oxo acid dehydrogenase, lipoyl-binding site, Glycine cleavage H-protein, s	93.75	45.76	1.03	0.00000
Nitab4.5_0008119g0010	Glycoside hydrolase, catalytic domain, Glycosyl hydrolase, family 13, catalyt	21.61	10.53	1.04	0.00209
Nitab4.5_0000033g0010	SWEET sugar transporter	396.10	192.98	1.04	0.00000
Nitab4.5_0000357g0340	Glycoside hydrolase, superfamily, Glycoside hydrolase, family 17, Glycoside	28.53	13.90	1.04	0.00137
Nitab4.5_0002456g0090	SAM dependent carboxyl methyltransferase	42.04	20.48	1.04	0.00008
Nitab4.5_0000611g0110	Photosystem I PsaG, plant, Photosystem I PsaG/PsaK protein, Photosystem	417.23	202.98	1.04	0.00000
Nitab4.5_0008194g0060	PetM of cytochrome b6/f complex subunit 7	528.45	256.75	1.04	0.00000
Nitab4.5_0004146g0020	Serine/threonine-protein kinase, active site, Concanavalin A-like lectin/gluca	17.39	8.45	1.04	0.00156
Nitab4.5_0002356g0060	Transferase, Chloramphenicol acetyltransferase-like domain	42.49	20.62	1.04	0.00941
Nitab4.5_0004129g0010	2Fe-2S ferredoxin-type domain, Beta-grasp domain, Ferredoxin [2Fe-2S], pl	2907.19	1410.79	1.04	0.00000
Nitab4.5_0006319g0030		30.74	14.90	1.04	0.00002
Nitab4.5_0010777g0020	Pectinesterase inhibitor domain, Pectin lyase fold, Pectinesterase, catalytic,	34.53	16.74	1.04	0.00000
Nitab4.5_0007963g0010	Glycine cleavage T-protein, C-terminal barrel, Glycine cleavage system T pr	127.93	61.99	1.05	0.00016
Nitab4.5_0003353g0030	Concanavalin A-like lectin/glucanases superfamily, Xyloglucan endotransglu	35.57	17.23	1.05	0.00991
Nitab4.5_0009855g0010		16546.22	7999.43	1.05	0.00000
Nitab4.5_0000639g0060	Chlorophyll a/b binding protein domain, Chlorophyll A-B binding protein, plar	13.19	6.37	1.05	0.00253
Nitab4.5_0009028g0040	Protein of unknown function DUF2996	34.83	16.81	1.05	0.00000
Nitab4.5_0001049g0090	Glycoside hydrolase, family 19, catalytic, Glycoside hydrolase, family 19, Lys	94.70	45.68	1.05	0.00014
Nitab4.5_0004552g0010	Nucleotide sugar epimerase, NAD(P)-binding domain, NAD-dependent epim	77.55	37.41	1.05	0.04183
Nitab4.5_0002687g0020	Major facilitator superfamily, Major facilitator superfamily domain, Major facili	30.54	14.73	1.05	0.00006

Nitab4.5_0002892g0010	Cyanobacterial aminoacyl-tRNA synthetase, CAAD domain	216.47	104.33	1.05	0.00000
Nitab4.5_0001856g0010	NAD(P)H-quinone oxidoreductase, subunit N	138.74	66.84	1.05	0.00000
Nitab4.5_0001022g0030	Trimeric LpxA-like, ADP-glucose pyrophosphorylase, conserved site, Glucos	90.06	43.38	1.05	0.00000
Nitab4.5_0001417g0050	GNAT domain, Amino-acid N-acetyltransferase (ArgA), Aspartate/glutamate/	32.46	15.61	1.06	0.00025
Nitab4.5_0001096g0020	Triose-phosphate transporter domain	30.68	14.75	1.06	0.00121
Nitab4.5_0000980g0220	Pollen Ole e 1 allergen/extensin	134.28	64.53	1.06	0.00027
Nitab4.5_0005596g0080		596.46	286.45	1.06	0.00032
Nitab4.5_0000722g0100	ATPase, AAA-type, core, P-loop containing nucleoside triphosphate hydrolas	57.45	27.59	1.06	0.00014
Nitab4.5_0006341g0020	Globin, structural domain, Globin-like, Globin	113.01	54.27	1.06	0.00000
Nitab4.5_0007026g0040	NAC domain	52.91	25.40	1.06	0.00006
Nitab4.5_0000754g0260	Phenylalanine ammonia-lyase, shielding domain, Fumarase/histidase, N-terr	97.33	46.71	1.06	0.00212
Nitab4.5_0000082g0460	Mitochondrial carrier domain, Mitochondrial substrate/solute carrier	39.97	19.15	1.06	0.00000
Nitab4.5_0002859g0140	Signal peptide, camelysin, Photosystem I PsaH, reaction centre subunit VI	132.43	63.42	1.06	0.00016
Nitab4.5_0000709g0070	ACT domain	15.46	7.40	1.06	0.00201
Nitab4.5_0012694g0010	NAD(P)-binding domain	16.78	8.02	1.07	0.00171
Nitab4.5_0000263g0230		26.37	12.57	1.07	0.00002
Nitab4.5_0006395g0010	CidB/LrgB family	62.55	29.81	1.07	0.00000
Nitab4.5_0000535g0120	NAC domain	23.90	11.37	1.07	0.00011
Nitab4.5_0006833g0020	Tetratricopeptide repeat-containing domain, Tetratricopeptide-like helical	115.83	55.07	1.07	0.00001
Nitab4.5_0013227g0020	Lipase, class 3	46.51	22.09	1.07	0.00243
Nitab4.5_0001401g0180	PetM of cytochrome b6/f complex subunit 7	360.51	171.17	1.07	0.00000
Nitab4.5_0009018g0010		18.55	8.81	1.07	0.00221
Nitab4.5_0001622g0160	Photosystem I PsaG/PsaK protein, Photosystem I PsaG, plant, Photosystem	178.31	84.21	1.08	0.00004
Nitab4.5_0000040g0080	PsbQ-like domain, Photosystem II PsbQ, oxygen evolving complex	38.36	18.09	1.08	0.00001
Nitab4.5_0011986g0020		1321.88	622.94	1.09	0.00000
Nitab4.5_0000128g0170	Serine hydrolase FSH	50.70	23.86	1.09	0.00012
Nitab4.5_0002695g0040	Glycine cleavage T-protein, N-terminal, GTP-binding protein TrmE/Glycine c	142.36	66.95	1.09	0.00001
Nitab4.5_0007026g0030	Glycine cleavage H-protein, Glycine cleavage H-protein, subgroup, 2-oxo ac	331.91	155.99	1.09	0.00000
Nitab4.5_0000131g0030	Chloroquine resistance transporter-related	26.52	12.44	1.09	0.00031
Nitab4.5_0005266g0020	Ornithine/DAP/Arg decarboxylase, Orn/DAP/Arg decarboxylase 2, pyridoxal-	36.61	17.15	1.09	0.00124
Nitab4.5_0002568g0020		19.35	9.06	1.10	0.00055
Nitab4.5_0001003g0130	Chloramphenicol acetyltransferase-like domain, Transferase	30.55	14.28	1.10	0.00030
Nitab4.5_0000123g0370	Cytochrome P450, conserved site, Cytochrome P450, E-class, group I, Cyto	47.63	22.24	1.10	0.00009
Nitab4.5_0001003g0100	HAD-like domain, HAD-superfamily hydrolase, subfamily IIA, 2-phosphoglyc	52.65	24.57	1.10	0.00000
Nitab4.5_0000976g0110	Rubredoxin domain, Rubredoxin-type fold, Rubredoxin-like domain	52.60	24.51	1.10	0.00000
Nitab4.5_0002816g0020	Peroxisomal biogenesis factor 11	23.30	10.85	1.10	0.00010
Nitab4.5_0000783g0230	NAD(P)-binding domain, NmrA-like	21.43	9.97	1.10	0.00020
Nitab4.5_0001382g0020	Ribulose-1,5-bisphosphate carboxylase small subunit, N-terminal, Ribulose I	4833.56	2248.50	1.10	0.00000
Nitab4.5_0009646g0060	NAD(P)-binding domain, Glyceraldehyde 3-phosphate dehydrogenase, activ	211.55	98.14	1.11	0.00000

Nitab4.5_0006960g0010	Catalase active site, Catalase core domain, Catalase, mono-functional, haer	73.06	33.88	1.11	0.00007
Nitab4.5_0001034g0100	Cyclin-dependent kinase inhibitor, plant, Cyclin-dependent kinase inhibitor	28.85	13.37	1.11	0.00001
Nitab4.5_0002637g0020	Protein of unknown function DUF3464	61.42	28.42	1.11	0.00001
Nitab4.5_0021741g0010	GH3 auxin-responsive promoter	59.56	27.56	1.11	0.00664
Nitab4.5_0005926g0020	Fructose-1,6-bisphosphatase class 1/Sedoheputulose-1,7-bisphosphatase, I	53.56	24.77	1.11	0.00000
Nitab4.5_0002990g0020		69.93	32.31	1.11	0.00000
Nitab4.5_0000059g0010	Glutamine synthetase, beta-Grasp, Glutamine synthetase/guanido kinase, ca	141.31	65.09	1.12	0.00001
Nitab4.5_0002600g0010	Ammonium transporter, Ammonium transporter AmtB-like domain, Ammoniu	54.17	24.86	1.12	0.00048
Nitab4.5_0001770g0160	SCAMP	12.99	5.95	1.13	0.00244
Nitab4.5_0003142g0030		82.99	37.98	1.13	0.02966
Nitab4.5_0005090g0030	Protein of unknown function DUF506, plant	27.57	12.61	1.13	0.00008
Nitab4.5_0009478g0010	UspA, Concanavalin A-like lectin/glucanase, subgroup, Protein kinase, ATP	17.50	8.00	1.13	0.00031
Nitab4.5_0000032g0010	Fructose-1,6-bisphosphatase, Fructose-1,6-bisphosphatase class 1/Sedohe	72.97	33.31	1.13	0.00000
Nitab4.5_0008168g0010	Tetratricopeptide repeat-containing domain, Tetratricopeptide-like helical	177.45	80.99	1.13	0.00000
Nitab4.5_0002229g0110	Photosystem II PsbQ, oxygen evolving complex, PsbQ-like domain	36.39	16.61	1.13	0.00000
Nitab4.5_0004304g0040	Glycosyl transferase, family 35	22.86	10.43	1.13	0.00007
Nitab4.5_0014926g0010		25.72	11.73	1.13	0.00016
Nitab4.5_0000152g0250	Phosphoribulokinase/uridine kinase, Fructose-bisphosphate aldolase, class-	35.33	16.11	1.13	0.00001
Nitab4.5_0005336g0020	AMP-binding, conserved site, AMP-dependent synthetase/ligase, AMP-bindi	21.41	9.76	1.13	0.00149
Nitab4.5_0008471g0020	NAD(P)-binding domain, Alcohol dehydrogenase superfamily, zinc-type, Gro	131.85	60.10	1.13	0.00072
Nitab4.5_0012156g0030		145.12	66.10	1.13	0.02347
Nitab4.5_0000278g0170	Sedoheptulose-1,7-bisphosphatase, Fructose-1,6-bisphosphatase class 1/S	135.41	61.62	1.14	0.00000
Nitab4.5_0001982g0070	Proline dehydrogenase, Proline oxidase	130.08	59.09	1.14	0.00104
Nitab4.5_0007189g0010	Chlorophyll A-B binding protein, Chlorophyll A-B binding protein, plant, Chlor	16.55	7.51	1.14	0.00066
Nitab4.5_0006769g0070	Protein kinase, ATP binding site, Protein kinase-like domain, Protein kinase	17.41	7.90	1.14	0.00046
Nitab4.5_0005790g0010	Amino acid transporter, transmembrane	42.48	19.24	1.14	0.00009
Nitab4.5_0008490g0010	PMR5 N-terminal domain, PC-Esterase	16.75	7.58	1.14	0.00097
Nitab4.5_0002064g0060	Plastid lipid-associated protein/fibrillin conserved domain	14.52	6.56	1.15	0.00124
Nitab4.5_0000678g0100	Domain of unknown function DUF4378, Protein of unknown function DUF37	21.22	9.59	1.15	0.00013
Nitab4.5_0000721g0130	FAS1 domain	22.64	10.23	1.15	0.00015
Nitab4.5_0001703g0040	Macrophage migration inhibitory factor, Tautomerase/MIF superfamily	190.42	85.95	1.15	0.00000
Nitab4.5_0000454g0030	Protein of unknown function DUF3474, Fatty acid desaturase, type 1	25.85	11.67	1.15	0.00042
Nitab4.5_0001361g0190	NAD(P)-binding domain	17.46	7.86	1.15	0.00037
Nitab4.5_0000288g0030	Glycoside hydrolase, family 9, Six-hairpin glycosidase, Glycoside hydrolase,	34.11	15.34	1.15	0.00278
Nitab4.5_0002612g0080	Protein phosphatase 2C (PP2C)-like domain, Protein phosphatase 2C	13.40	6.02	1.15	0.00253
Nitab4.5_0002435g0010	Photosystem II Psb28, class 1	26.61	11.91	1.16	0.00004
Nitab4.5_0000244g0140	Chloramphenicol acetyltransferase-like domain, Transferase	22.22	9.94	1.16	0.00009
Nitab4.5_0002222g0060		54.74	24.48	1.16	0.00000
Nitab4.5_0000702g0130	Catalase core domain, Catalase, mono-functional, haem-containing, Catalas	53.76	24.04	1.16	0.00008

Nitab4.5_0007096g0040	LanC-like protein, eukaryotic, Lanthionine synthetase C-like	15.31	6.82	1.17	0.00354
Nitab4.5_0005734g0020	Ammonium transporter AmtB-like domain, Ammonium transporter, Ammoniu	120.97	53.82	1.17	0.00002
Nitab4.5_0004944g0030	Auxin efflux carrier	255.97	113.84	1.17	0.00010
Nitab4.5_0008218g0020	Oligopeptide transporter, OPT superfamily	19.79	8.80	1.17	0.00020
Nitab4.5_0007144g0010		243.77	108.17	1.17	0.00037
Nitab4.5_0001918g0100	FAS1 domain	21.56	9.56	1.17	0.00004
Nitab4.5_0002709g0020		12.73	5.64	1.17	0.00349
Nitab4.5_0001936g0030	Plastocyanin, Cupredoxin, Blue (type 1) copper protein, Blue (type 1) copper	956.19	423.13	1.18	0.00003
Nitab4.5_0000317g0110	NAD(P)H-quinone oxidoreductase subunit M	54.57	24.14	1.18	0.00000
Nitab4.5_0001429g0010	Protein of unknown function DUF538	50.43	22.30	1.18	0.00000
Nitab4.5_0008829g0010	Protein of unknown function DUF1817	44.33	19.58	1.18	0.00000
Nitab4.5_0002424g0040	P-loop containing nucleoside triphosphate hydrolase	33.46	14.78	1.18	0.00004
Nitab4.5_0002209g0120	Chlorophyll A-B binding protein, plant, Chlorophyll A-B binding protein, Chlor	54.41	24.02	1.18	0.00030
Nitab4.5_0004168g0090		25.77	11.34	1.19	0.00009
Nitab4.5_0000194g0060	Pollen Ole e 1 allergen/extensin	229.20	100.72	1.19	0.00033
Nitab4.5_0000102g0040	FAD-dependent pyridine nucleotide-disulphide oxidoreductase, Pyridine nuc	20.66	9.07	1.19	0.00017
Nitab4.5_0001257g0060	SWEET sugar transporter	26.55	11.65	1.19	0.00001
Nitab4.5_0000241g0020		37.86	16.60	1.19	0.00651
Nitab4.5_0000386g0040	HAD-like domain	23.22	10.17	1.19	0.00006
Nitab4.5_0008779g0020	Bet v I domain, START-like domain	68.11	29.84	1.19	0.00000
Nitab4.5_0000552g0120	Protein of unknown function DUF538	87.41	38.28	1.19	0.03920
Nitab4.5_0001584g0050	Pollen specific protein SF21, Ndr	15.97	6.99	1.19	0.00322
Nitab4.5_0003070g0010	Glutamine synthetase, catalytic domain, Glutamine synthetase, beta-Grasp,	148.52	64.92	1.19	0.00001
Nitab4.5_0004438g0050		338.34	147.76	1.20	0.00000
Nitab4.5_0000099g0190	Short-chain dehydrogenase/reductase SDR, NAD(P)-binding domain, Gluco:	43.21	18.85	1.20	0.00001
Nitab4.5_0010626g0010	SWEET sugar transporter	208.24	90.81	1.20	0.00000
Nitab4.5_0010531g0010	Cytochrome P450, Cytochrome P450, conserved site, Cytochrome P450, E-	30.24	13.18	1.20	0.00038
Nitab4.5_0000578g0110	Protein of unknown function DUF4079, Di-haem cytochrome, transmembran	10.99	4.77	1.20	0.00290
Nitab4.5_0008525g0020	Haem peroxidase, plant/fungal/bacterial, Haem peroxidase	149.71	64.63	1.21	0.00001
Nitab4.5_0007209g0010		65.76	28.35	1.21	0.00714
Nitab4.5_0007468g0020	ADP-glucose pyrophosphorylase, conserved site, Nucleotidyl transferase, Tr	129.78	55.92	1.21	0.00011
Nitab4.5_0004057g0050	Macrophage migration inhibitory factor, Tautomerase/MIF superfamily	97.15	41.84	1.22	0.00002
Nitab4.5_0000646g0210	Rhodanese-like domain	73.34	31.55	1.22	0.00001
Nitab4.5_0001504g0080	Protein of unknown function DUF3148	58.56	25.11	1.22	0.00000
Nitab4.5_0011020g0010	Protein of unknown function DUF1218	18.27	7.83	1.22	0.00013
Nitab4.5_0002137g0140	Triose-phosphate transporter domain, Triose phosphate/phosphoenolpyruva	46.99	20.07	1.23	0.00610
Nitab4.5_0005017g0040		17.31	7.39	1.23	0.00045
Nitab4.5_0001249g0020	Mitochondrial carrier domain, Mitochondrial substrate/solute carrier	25.97	11.08	1.23	0.00000
Nitab4.5_0000937g0110	Photosystem I PsaO	635.19	270.49	1.23	0.00016

Nitab4.5_0010803g0010	Protein of unknown function DUF3474, Fatty acid desaturase, type 1	26.84	11.42	1.23	0.00000
Nitab4.5_0003546g0010	Deoxyxylulose-5-phosphate synthase, Transketolase, C-terminal/Pyruvate-fe	51.15	21.72	1.24	0.00000
Nitab4.5_0000105g0250	Cytochrome P450, E-class, group I, Cytochrome P450, Cytochrome P450, c	95.81	40.68	1.24	0.00069
Nitab4.5_0000783g0240	Flavin monooxygenase-like	18.84	7.99	1.24	0.00039
Nitab4.5_0002741g0110		107.67	45.53	1.24	0.00116
Nitab4.5_0006179g0030	Protein phosphatase 2C (PP2C)-like domain, Protein phosphatase 2C	19.34	8.17	1.24	0.00010
Nitab4.5_0005250g0030	Photosystem I PsaF, reaction centre subunit III	68.52	28.91	1.24	0.00095
Nitab4.5_0000476g0010	Zinc finger, GATA-type, Transcription factor, GATA, plant, Zinc finger, NHR/(	15.77	6.65	1.25	0.00055
Nitab4.5_0006483g0010	Glutamine amidotransferase	22.39	9.43	1.25	0.00031
Nitab4.5_0002601g0090	NUDIX hydrolase domain, NUDIX hydrolase, conserved site, NUDIX hydrola	20.45	8.61	1.25	0.00005
Nitab4.5_0000173g0030	Aquaporin-like, Major intrinsic protein, Major intrinsic protein, conserved site	20.85	8.75	1.25	0.00159
Nitab4.5_0015171g0010	Fructose-1,6-bisphosphatase class 1/Sedoheputulose-1,7-bisphosphatase, S	210.06	88.07	1.25	0.00000
Nitab4.5_0002545g0050	Cytochrome P450, Cytochrome P450, conserved site, Cytochrome P450, E-	181.12	75.72	1.26	0.00000
Nitab4.5_0009304g0010	von Willebrand factor, type A	13.92	5.81	1.26	0.00044
Nitab4.5_0003282g0010	SGNH hydrolase-type esterase domain, Lipase, GDSL	12.56	5.24	1.26	0.00223
Nitab4.5_0008041g0020	Cytochrome P450, Cytochrome P450, E-class, group I, Cytochrome P450, c	10.97	4.58	1.26	0.00177
Nitab4.5_0002342g0120	PsbQ-like domain, Photosystem II PsbQ, oxygen evolving complex	48.86	20.33	1.27	0.00000
Nitab4.5_0001454g0050	Double-stranded RNA-binding domain	41.94	17.43	1.27	0.00237
Nitab4.5_0004144g0020	Thylakoid soluble phosphoprotein TSP9	694.71	288.45	1.27	0.00000
Nitab4.5_0000937g0070	Pollen Ole e 1 allergen/extensin	40.54	16.82	1.27	0.00000
Nitab4.5_0010009g0010	Proteinase inhibitor I20, Pin2	91.24	37.77	1.27	0.00000
Nitab4.5_0008803g0020	Alcohol dehydrogenase GroES-like, NAD(P)-binding domain, GroES (chapei	16.20	6.70	1.27	0.00015
Nitab4.5_0005452g0030	Protein of unknown function DUF3529	22.15	9.14	1.28	0.00003
Nitab4.5_0000713g0080	Ndr, Pollen specific protein SF21	20.80	8.56	1.28	0.00003
Nitab4.5_0010331g0010		27.30	11.23	1.28	0.00091
Nitab4.5_0000173g0130	Superoxide dismutase (Cu/Zn) / superoxide dismutase copper chaperone, S	69.57	28.60	1.28	0.00000
Nitab4.5_0001783g0130	Plastid lipid-associated protein/fibrillin conserved domain	48.21	19.80	1.28	0.00000
Nitab4.5_0000059g0410	P-loop containing nucleoside triphosphate hydrolase, ABC transporter-like, A	35.63	14.63	1.28	0.00004
Nitab4.5_0001215g0040	Serine-threonine/tyrosine-protein kinase catalytic domain, Serine/threonine-	26.56	10.87	1.29	0.00000
Nitab4.5_0003759g0050	Peptidase S10, serine carboxypeptidase, Peptidase S10, serine carboxypep	73.63	30.11	1.29	0.00000
Nitab4.5_0000543g0120	CidB/LrgB family	53.12	21.67	1.29	0.00000
Nitab4.5_0000380g0150	Late embryogenesis abundant protein, LEA-14	13.57	5.53	1.30	0.00067
Nitab4.5_0000588g0280	Cupredoxin, Multicopper oxidase, type 2, Multicopper oxidase, type 3, Multic	12.42	5.06	1.30	0.00253
Nitab4.5_0000635g0100	GH3 auxin-responsive promoter	51.58	21.00	1.30	0.00029
Nitab4.5_0002728g0020	Homeodomain-like, Myb domain, SANT/Myb domain	14.78	6.01	1.30	0.00070
Nitab4.5_0000007g0300	Ribulose bisphosphate carboxylase, large subunit, C-terminal, Ribulose bisp	42.68	17.34	1.30	0.00466
Nitab4.5_0000181g0100		13.64	5.54	1.30	0.00087
Nitab4.5_0000667g0150		35.06	14.23	1.30	0.00261
Nitab4.5_0002902g0020	Cytochrome P450, conserved site, Cytochrome P450, Cytochrome P450, E-	74.88	30.39	1.30	0.00006

Nitab4.5_0000422g0020	Gibberellin regulated protein	48.58	19.68	1.30	0.00012
Nitab4.5_0000351g0060	Photosystem I PsaH, reaction centre subunit VI, Signal peptide, camelysin	55.57	22.50	1.30	0.00001
Nitab4.5_0003884g0100		36.22	14.65	1.31	0.00001
Nitab4.5_0005019g0070	Nodulin-like, Major facilitator superfamily domain, general substrate transpor	11.91	4.81	1.31	0.00201
Nitab4.5_0004234g0050	Chlorophyll A-B binding protein, Chlorophyll A-B binding protein, plant, Chlor	44.23	17.85	1.31	0.00000
Nitab4.5_0004053g0080	Uncharacterised protein family UPF0114, Uncharacterised protein family UP	45.04	18.18	1.31	0.00000
Nitab4.5_0000201g0130	Tify, CO/COL/TOC1, conserved site	39.21	15.82	1.31	0.00040
Nitab4.5_0001112g0030	Phosphoribulokinase/uridine kinase, Uridine kinase, Fructose-bisphosphate a	31.81	12.82	1.31	0.00000
Nitab4.5_0007669g0020		101.30	40.78	1.31	0.00000
Nitab4.5_0002003g0050		49.70	20.00	1.31	0.00000
Nitab4.5_0011538g0020		55.48	22.29	1.32	0.00035
Nitab4.5_0000578g0180		34.98	14.04	1.32	0.00158
Nitab4.5_0000551g0070	Domain of unknown function DUF239, Domain of unknown function DUF440	22.73	9.12	1.32	0.00000
Nitab4.5_0001201g0020	START-like domain, Bet v I domain	26.52	10.63	1.32	0.00012
Nitab4.5_0008594g0010	Ammonium transporter, Ammonium transporter AmtB-like domain, Ammoniu	27.87	11.17	1.32	0.00001
Nitab4.5_0000123g0380	Peptidyl-prolyl cis-trans isomerase, FKBP-type, domain, Tetratricopeptide re	16.21	6.47	1.32	0.00079
Nitab4.5_0002449g0130	Myc-type, basic helix-loop-helix (bHLH) domain	11.37	4.54	1.32	0.00113
Nitab4.5_0002417g0020	FAS1 domain	15.82	6.31	1.33	0.00365
Nitab4.5_0000864g0130	Thaumatin, Thaumatin, conserved site	13.71	5.46	1.33	0.00026
Nitab4.5_0005994g0020	GDP-fucose protein O-fucosyltransferase, O-fucosyltransferase, plant	15.33	6.10	1.33	0.00032
Nitab4.5_0000043g0320	Photosystem I PsaD	96.44	38.36	1.33	0.00002
Nitab4.5_0003914g0040	Major intrinsic protein, Aquaporin-like, Major intrinsic protein, conserved site	47.04	18.69	1.33	0.00000
Nitab4.5_0003030g0010	Plastocyanin, Cupredoxin, Blue (type 1) copper protein, Blue (type 1) copper	700.57	278.27	1.33	0.00000
Nitab4.5_0007459g0020	P-type ATPase, A domain, HAD-like domain, Cation-transporting P-type AT	16.62	6.60	1.33	0.00010
Nitab4.5_0000559g0020		74.51	29.52	1.34	0.00046
Nitab4.5_0007293g0050	Major intrinsic protein, Aquaporin-like	24.51	9.70	1.34	0.00000
Nitab4.5_0008665g0010	Photosystem II protein D1, Photosynthetic reaction centre, L/M	17.09	6.76	1.34	0.00275
Nitab4.5_0004044g0020	Chlorophyll A-B binding protein, Chlorophyll a/b binding protein domain	93.29	36.83	1.34	0.00000
Nitab4.5_0004342g0050	Rhodanese-like domain	79.44	31.33	1.34	0.00013
Nitab4.5_0009676g0030	Basic-leucine zipper domain	12.95	5.10	1.34	0.00058
Nitab4.5_0007813g0010	Zinc finger, C2H2	18.03	7.06	1.35	0.00003
Nitab4.5_0003820g0010	Thioredoxin-like fold, Glutathione S-transferase, C-terminal-like, Glutathione	48.01	18.74	1.36	0.00000
Nitab4.5_0008639g0010	Peptidyl-prolyl cis-trans isomerase, FKBP-type, domain, Peptidyl-prolyl cis-tr	20.49	7.98	1.36	0.00002
Nitab4.5_0001829g0010	Rhodanese-like domain	28.50	11.10	1.36	0.00000
Nitab4.5_0000659g0120	Ribulose bisphosphate carboxylase, small chain, Ribulose-1,5-bisphosphate	139.80	54.39	1.36	0.00000
Nitab4.5_0001194g0110	Drug/metabolite transporter	14.55	5.66	1.36	0.00074
Nitab4.5_0005553g0020	Transferase, Chloramphenicol acetyltransferase-like domain	21.32	8.28	1.36	0.00001
Nitab4.5_0001332g0140	SWEET sugar transporter, Major facilitator superfamily domain, general subs	15.70	6.08	1.37	0.00014
Nitab4.5_0004300g0170	Photosystem II PsbQ, oxygen evolving complex, PsbQ-like domain	47.67	18.41	1.37	0.00000

Nitab4.5_0004290g0040		26.02	10.02	1.38	0.00002
Nitab4.5_0004160g0030	Pollen specific protein SF21, Ndr	37.78	14.54	1.38	0.00000
Nitab4.5_0001123g0060	Rhodanese-like domain	10.18	3.92	1.38	0.00238
Nitab4.5_0014443g0010	Major intrinsic protein, Major intrinsic protein, conserved site, Aquaporin-like	19.83	7.63	1.38	0.00042
Nitab4.5_0000956g0150	Major intrinsic protein, Aquaporin-like	31.76	12.19	1.38	0.00000
Nitab4.5_0006950g0010	Amino acid transporter, transmembrane	21.63	8.29	1.38	0.00000
Nitab4.5_0001755g0100		97.45	37.34	1.38	0.00001
Nitab4.5_0005901g0010	Cytochrome P450, Cytochrome P450, conserved site, Cytochrome P450, E-	49.86	19.09	1.38	0.00278
Nitab4.5_0000586g0080		34.65	13.26	1.39	0.00000
Nitab4.5_0001922g0120	Terpenoid synthase, Polyprenyl synthetase, Polyprenyl synthetase-related	94.56	36.14	1.39	0.00000
Nitab4.5_0000895g0250	NAF/FISL domain, Protein kinase domain, Serine/threonine- / dual specificity	13.49	5.15	1.39	0.00058
Nitab4.5_0003796g0010	Lipoxygenase, C-terminal, Lipoxygenase, iron binding site, PLAT/LH2 domai	36.43	13.89	1.39	0.00000
Nitab4.5_0000194g0070		55.56	21.17	1.39	0.00000
Nitab4.5_0000116g0280	Parallel beta-helix repeat, Glycoside hydrolase, family 28, Pectin lyase fold, I	11.98	4.56	1.39	0.00051
Nitab4.5_0005360g0020	DnaJ domain	33.68	12.80	1.40	0.00175
Nitab4.5_0002980g0050	Glycosyl transferase, family 1, Glycogen/starch synthase, ADP-glucose type	165.72	62.94	1.40	0.00000
Nitab4.5_0002809g0030	Tetratricopeptide-like helical	10.96	4.15	1.40	0.00151
Nitab4.5_0006914g0020	NAD(P)-binding domain	95.66	36.09	1.41	0.00000
Nitab4.5_0006744g0060	Peptidase S10, serine carboxypeptidase, Peptidase S10, serine carboxypep	209.77	78.93	1.41	0.00000
Nitab4.5_0000410g0380		19.69	7.38	1.42	0.00134
Nitab4.5_0000564g0020	Glycogen/starch synthase, ADP-glucose type, Starch synthase, catalytic dor	34.76	13.03	1.42	0.00000
Nitab4.5_0001103g0040	Chlorophyll a/b binding protein domain	17.31	6.48	1.42	0.00002
Nitab4.5_0000001g0050		27.68	10.35	1.42	0.00003
Nitab4.5_0001489g0020	Sucrose-phosphate synthase, Sucrose phosphate synthase, plant, HAD-like	37.59	14.05	1.42	0.00000
Nitab4.5_0000322g0160	Chlorophyll a/b binding protein domain, Chlorophyll A-B binding protein	104.09	38.87	1.42	0.00000
Nitab4.5_0000073g0060	Photosystem II PsbX	237.61	88.65	1.42	0.00000
Nitab4.5_0005537g0040	Carbohydrate binding domain CBM49, Glycoside hydrolase, family 9, Six-ha	52.74	19.66	1.42	0.00000
Nitab4.5_0000097g0010	P-loop containing nucleoside triphosphate hydrolase, Small GTPase superfa	15.09	5.62	1.42	0.00123
Nitab4.5_0002706g0040		21.02	7.83	1.42	0.00020
Nitab4.5_0000023g0490		84.37	31.43	1.42	0.00298
Nitab4.5_0002259g0020	Cytochrome P450, Cytochrome P450, conserved site, Cytochrome P450, E-	21.55	8.01	1.43	0.00007
Nitab4.5_0013252g0010	BURP domain	20.81	7.73	1.43	0.00334
Nitab4.5_0002097g0100	Cyclin-dependent kinase inhibitor, plant, Cyclin-dependent kinase inhibitor	33.15	12.31	1.43	0.00001
Nitab4.5_0002084g0070	NAD(P)-binding domain	107.26	39.71	1.43	0.00000
Nitab4.5_0001306g0030	BAG domain, Ubiquitin supergroup	22.24	8.22	1.44	0.00000
Nitab4.5_0001375g0050	SGNH hydrolase-type esterase domain, Lipase, GDSL	10.97	4.05	1.44	0.00164
Nitab4.5_0008995g0010	UDP-glucuronosyl/UDP-glucosyltransferase	9.34	3.42	1.45	0.00199
Nitab4.5_0009181g0010	Knottin, scorpion toxin-like, Gamma thionin, Gamma Purothionin	85.22	31.18	1.45	0.00005
Nitab4.5_0000300g0180	Acid phosphatase (Class B), HAD-like domain, Acid phosphatase, plant, Veç	421.31	153.71	1.45	0.00044

Nitab4.5_0000106g0330	Chaperonin Cpn60, GroEL-like equatorial domain, Chaperonin Cpn60, conse	20.94	7.61	1.46	0.00093
Nitab4.5_0000268g0090		13.82	5.01	1.46	0.00039
Nitab4.5_0000072g0600	Pectinesterase inhibitor domain	44.13	15.91	1.47	0.00001
Nitab4.5_0000329g0220	Xyloglucan endotransglucosylase/hydrolase, Xyloglucan endo-transglycosyla	14.98	5.37	1.48	0.00137
Nitab4.5_0003155g0010	Aquaporin-like, Major intrinsic protein, Major intrinsic protein, conserved site	600.76	214.98	1.48	0.00000
Nitab4.5_0003793g0010	Protein of unknown function DUF789	11.44	4.09	1.48	0.00101
Nitab4.5_0003627g0010	Pyridoxal phosphate-dependent transferase, Glycine cleavage system P pro	128.11	45.81	1.48	0.00000
Nitab4.5_0001439g0110	Photosynthetic reaction centre, L/M, Photosystem II protein D1	18.38	6.51	1.50	0.00546
Nitab4.5_0003961g0020	Photosynthetic reaction centre, L/M	88.69	31.39	1.50	0.00146
Nitab4.5_0010144g0020	Proteinase inhibitor, propeptide, Proteinase inhibitor I9	16.27	5.74	1.50	0.00005
Nitab4.5_0003381g0060	Peptidase M20, Amidohydrolase, Peptidase M20, dimerisation domain	13.16	4.64	1.50	0.00011
Nitab4.5_0008046g0010	Major latex protein domain, Bet v I domain, START-like domain	20.38	7.15	1.51	0.00397
Nitab4.5_0001761g0230	Photosynthetic reaction centre, L/M	28.24	9.85	1.52	0.01855
Nitab4.5_0006255g0010		45.86	15.99	1.52	0.00148
Nitab4.5_0001036g0140		33.49	11.65	1.52	0.00389
Nitab4.5_0007540g0030	Thioredoxin-like fold, Alkyl hydroperoxide reductase subunit C/ Thiol specific	20.50	7.11	1.53	0.00002
Nitab4.5_0000108g0440	Putative S-adenosyl-L-methionine-dependent methyltransferase	11.81	4.09	1.53	0.00022
Nitab4.5_0010563g0010	Amino acid transporter, transmembrane	7.20	2.48	1.54	0.00292
Nitab4.5_0004125g0010	FAS1 domain	17.30	5.94	1.54	0.00144
Nitab4.5_0002825g0020	Galactose mutarotase-like domain, Glycoside hydrolase-type carbohydrate-b	23.73	8.13	1.55	0.00000
Nitab4.5_0006870g0030	UDP-glucuronosyl/UDP-glucosyltransferase	21.68	7.42	1.55	0.00001
Nitab4.5_0001463g0050	Dienelactone hydrolase	22.02	7.52	1.55	0.00000
Nitab4.5_0000402g0180	Alcohol dehydrogenase, C-terminal, GroES (chaperonin 10)-like, Alcohol del	16.77	5.72	1.55	0.00001
Nitab4.5_0001907g0090	Protein phosphatase 2C (PP2C)-like domain, Protein phosphatase 2C	9.66	3.28	1.56	0.00118
Nitab4.5_0000373g0200	Sterile alpha motif/pointed domain, Sterile alpha motif, type 2	10.80	3.64	1.57	0.00052
Nitab4.5_0008011g0010	Thaumatin, Thaumatin, conserved site	17.52	5.89	1.57	0.00043
Nitab4.5_0005024g0030	Dihydrodipicolinate reductase, N-terminal, NAD(P)-binding domain, Dihydroc	27.79	9.30	1.58	0.00000
Nitab4.5_0002259g0030	Cytochrome P450, E-class, group I, Cytochrome P450, Cytochrome P450, c	9.34	3.12	1.58	0.00179
Nitab4.5_0008011g0050		39.14	13.02	1.59	0.00000
Nitab4.5_0001187g0030	Zinc finger, RING/FYVE/PHD-type, Zinc finger, RING-type, Cellulose syntha	49.03	16.22	1.60	0.00001
Nitab4.5_0002891g0070	Chloramphenicol acetyltransferase-like domain, Transferase	11.16	3.69	1.60	0.00019
Nitab4.5_0005388g0050	NPH3 domain	9.18	3.02	1.60	0.00096
Nitab4.5_0003767g0020		14.60	4.80	1.61	0.00008
Nitab4.5_0000024g0130	Photosystem II PsbX	66.31	21.70	1.61	0.00001
Nitab4.5_0005320g0020	Phenylalanine ammonia-lyase, L-Aspartase-like, Phenylalanine/histidine amı	119.71	39.13	1.61	0.00000
Nitab4.5_0001699g0010	Thioredoxin-like fold, Alkyl hydroperoxide reductase subunit C/ Thiol specific	34.77	11.28	1.62	0.00000
Nitab4.5_0003186g0010		29.76	9.64	1.63	0.00000
Nitab4.5_0005018g0030	Protein of unknown function DUF3474, Fatty acid desaturase, type 1	36.38	11.78	1.63	0.00000
Nitab4.5_0003305g0040		2761.61	889.21	1.63	0.00051

Nitab4.5_0003337g0010	Carbonic anhydrase, Carbonic anhydrase, prokaryotic-like, conserved site	38.95	12.51	1.64	0.00000
Nitab4.5_0000091g0360	Squalene/phytoene synthase, Terpenoid synthase, Squalene/phytoene syntl	111.80	35.85	1.64	0.00000
Nitab4.5_0005463g0080	Fatty acid desaturase, type 1, Protein of unknown function DUF3474	6.87	2.20	1.64	0.00491
Nitab4.5_0007527g0010	Zinc finger, NHR/GATA-type, Zinc finger, GATA-type, Transcription factor, G	7.21	2.30	1.65	0.00292
Nitab4.5_0005616g0010	Dienelactone hydrolase	21.88	6.97	1.65	0.00000
Nitab4.5_0000203g0040	BURP domain	18.03	5.73	1.65	0.00000
Nitab4.5_0000026g0070	Isopenicillin N synthase-like, Oxoglutarate/iron-dependent dioxygenase	36.48	11.60	1.65	0.00000
Nitab4.5_0008691g0010	Transferase, Chloramphenicol acetyltransferase-like domain	16.51	5.23	1.66	0.00001
Nitab4.5_0000659g0130	Ribulose bisphosphate carboxylase, small chain, Ribulose bisphosphate car	67.83	21.42	1.66	0.00000
Nitab4.5_0004090g0120	Riboflavin synthase-like beta-barrel, Ferric reductase, NAD binding, FAD-bin	14.05	4.43	1.67	0.00002
Nitab4.5_0000003g0010	Major facilitator superfamily domain, general substrate transporter, Proton-de	11.37	3.58	1.67	0.00012
Nitab4.5_0000639g0240	Thioredoxin-like fold, Glutathione S-transferase, N-terminal, Glutathione S-tra	25.93	8.09	1.68	0.00497
Nitab4.5_0006742g0010	Pyridine nucleotide-disulphide oxidoreductase, FAD/NAD(P)-binding domain	12.92	4.03	1.68	0.00066
Nitab4.5_0010051g0020		13.80	4.28	1.69	0.00042
Nitab4.5_0000137g0280	Lipase, GDSL, SGNH hydrolase-type esterase domain	9.04	2.80	1.69	0.00023
Nitab4.5_0004468g0010	DOMON domain, Uncharacterised conserved protein UCP037471	19.87	6.13	1.70	0.00000
Nitab4.5_0000625g0080	Peptidase C26	7.36	2.26	1.70	0.00172
Nitab4.5_0001163g0070	Aquaporin-like, Major intrinsic protein, Major intrinsic protein, conserved site	228.86	70.18	1.71	0.00000
Nitab4.5_0006696g0020	Bifunctional inhibitor/plant lipid transfer protein/seed storage helical domain,	56.02	17.15	1.71	0.01981
Nitab4.5_0000737g0120	Aquaporin-like, Major intrinsic protein, Major intrinsic protein, conserved site	44.25	13.51	1.71	0.00000
Nitab4.5_0001231g0080	Carbohydrate binding domain CBM49, Glycoside hydrolase, family 9, active	36.17	11.02	1.71	0.00000
Nitab4.5_0000194g0050	Pollen Ole e 1 allergen/extensin	25.85	7.87	1.72	0.00127
Nitab4.5_0001348g0060	Peptidase S10, serine carboxypeptidase, Peptidase S10, serine carboxypep	17.92	5.37	1.74	0.00000
Nitab4.5_0004867g0070	RNA-binding S4 domain, Aminoacyl-tRNA synthetase, class Ic, Aminoacyl-tF	7.13	2.13	1.74	0.00142
Nitab4.5_0005967g0020	Protein of unknown function DUF2996	25.73	7.67	1.75	0.00000
Nitab4.5_0001120g0010	ABC transporter type 1, transmembrane domain, AAA+ ATPase domain, AB	35.60	10.59	1.75	0.00000
Nitab4.5_0009124g0010	Tryptophan synthase beta chain/beta chain-like, Tryptophan synthase, beta	9.57	2.85	1.75	0.00029
Nitab4.5_0006370g0020		325.32	96.76	1.75	0.00697
Nitab4.5_0004217g0030	F-box domain, F-box associated interaction domain	19.12	5.69	1.75	0.00000
Nitab4.5_0004863g0010	Lipase, GDSL, SGNH hydrolase-type esterase domain	34.76	10.34	1.75	0.00000
Nitab4.5_0020038g0010	Ribulose bisphosphate carboxylase small chain, domain, Ribulose bisphospl	283.81	83.87	1.76	0.00000
Nitab4.5_0001367g0020	Glycine cleavage system P protein, homodimeric, Glycine cleavage system I	143.49	42.39	1.76	0.00000
Nitab4.5_0000062g0240		37.34	10.92	1.77	0.02327
Nitab4.5_0000002g0310	Uncharacterised protein family Ycf68	29.31	8.57	1.77	0.00000
Nitab4.5_0001344g0020	Zinc finger, RING/FYVE/PHD-type, Zinc finger, RING-type, Cellulose syntha	38.71	11.31	1.78	0.00002
Nitab4.5_0007367g0100	Nucleoside diphosphate kinase	5.49	1.60	1.78	0.00511
Nitab4.5_0007055g0020	Drug/metabolite transporter, Triose-phosphate transporter domain	9.11	2.64	1.79	0.00010
Nitab4.5_0000787g0200	Glyoxalase-like domain	8.86	2.56	1.79	0.00458
Nitab4.5_0000134g0010	Auxin-induced protein, ARG7	6.62	1.89	1.79	0.00203

Nitab4.5_0000040g0160	Expansin/pollen allergen, DPBB domain, Barwin-like endoglucanase, Expans	14.33	4.10	1.81	0.00000
Nitab4.5_0006593g0010	Terpenoid synthase, Terpene synthase, N-terminal domain, Terpene synthas	73.27	20.96	1.81	0.00000
Nitab4.5_0001810g0050	Triose-phosphate transporter domain	55.57	15.76	1.82	0.00000
Nitab4.5_0000082g0150	Winged helix-turn-helix DNA-binding domain, Heat shock factor (HSF)-type,	21.41	6.06	1.82	0.00033
Nitab4.5_0010595g0010	AMP-binding, conserved site, AMP-dependent synthetase/ligase	54.36	15.37	1.82	0.00001
Nitab4.5_0001970g0050		45.29	12.69	1.84	0.00003
Nitab4.5_0011391g0010	Cytochrome P450, Cytochrome P450, conserved site, Cytochrome P450, E-	20.22	5.66	1.84	0.00000
Nitab4.5_0000200g0190	UDP-glucuronosyl/UDP-glucosyltransferase	44.68	12.50	1.84	0.00002
Nitab4.5_0000006g0100	Triose phosphate/phosphoenolpyruvate translocator, Drug/metabolite transp	184.13	51.47	1.84	0.00003
Nitab4.5_0000677g0170	Pyridoxal phosphate-dependent transferase, major region, subdomain 1, Alli	9.40	2.62	1.84	0.00031
Nitab4.5_0004422g0010	Cupin 1, Germin, Germin, manganese binding site, RmlC-like cupin domain,	53.59	14.90	1.85	0.00000
Nitab4.5_0014182g0010	Xylan biosynthesis protein IRX15/IRX15L, Putative polysaccharide biosynthe	9.86	2.74	1.85	0.00004
Nitab4.5_0002606g0050	Tetrahydrofolate dehydrogenase/cyclohydrolase, catalytic domain, Tetrahydı	26.36	7.27	1.86	0.00731
Nitab4.5_0019996g0010	SWEET sugar transporter	17.13	4.66	1.88	0.00000
Nitab4.5_0006563g0030	Photosystem II PsbX	36.59	9.77	1.90	0.00000
Nitab4.5_0006590g0030	Translocon-associated protein (TRAP), alpha subunit	8.53	2.27	1.91	0.00039
Nitab4.5_0006911g0020		33.56	8.89	1.92	0.00012
Nitab4.5_0000130g0230	Cytochrome P450, Cytochrome P450, E-class, group IV	34.30	9.07	1.92	0.00000
Nitab4.5_0000659g0110	Ribulose bisphosphate carboxylase small chain, domain, Ribulose bisphospl	118.54	31.02	1.93	0.00000
Nitab4.5_0011952g0020	Photosynthetic reaction centre, L/M	326.20	85.30	1.94	0.00013
Nitab4.5_0022030g0010	Nucleic acid-binding, OB-fold, Primosome PriB/single-strand DNA-binding, S	7.11	1.83	1.95	0.00195
Nitab4.5_0002421g0030		9.00	2.32	1.96	0.00041
Nitab4.5_0001908g0030	Homeodomain-like, Myb domain, plants, SANT/Myb domain, Myb domain	102.49	26.03	1.98	0.00000
Nitab4.5_0001866g0030	Photosystem II PsbX	176.77	44.39	1.99	0.00000
Nitab4.5_0000960g0060		14.21	3.56	2.00	0.00113
Nitab4.5_0004924g0070	Peptidase S8/S53 domain, Peptidase S8, subtilisin-related	15.19	3.80	2.00	0.00000
Nitab4.5_0000116g0370	Pectinesterase inhibitor domain, Pectinesterase, active site, Pectinesterase,	4.48	1.12	2.00	0.00567
Nitab4.5_0001960g0060	SANT domain, SANT/Myb domain, Homeodomain-like	104.04	25.77	2.01	0.00140
Nitab4.5_0008983g0010	Prephenate dehydratase, conserved site, Prephenate dehydratase	17.16	4.21	2.03	0.00015
Nitab4.5_0015078g0010	Auxin-induced protein, ARG7	21.24	5.18	2.03	0.00334
Nitab4.5_0001256g0060	Chloramphenicol acetyltransferase-like domain, Transferase	14.48	3.52	2.04	0.00000
Nitab4.5_0000748g0010	SGNH hydrolase-type esterase domain, Lipase, GDSL	6.74	1.63	2.05	0.00048
Nitab4.5_0007342g0020		15.13	3.63	2.06	0.00004
Nitab4.5_0001701g0170	FAS1 domain	10.52	2.52	2.06	0.00242
Nitab4.5_0005555g0020	Bet v I type allergen, Major latex protein domain, START-like domain, Bet v I	6.99	1.65	2.08	0.00112
Nitab4.5_0012647g0010	AMP-dependent synthetase/ligase, AMP-binding, conserved site, AMP-bindi	50.94	12.03	2.08	0.00000
Nitab4.5_0001492g0060	O-methyltransferase, family 3	21.01	4.95	2.08	0.00000
Nitab4.5_0001757g0020	Myc-type, basic helix-loop-helix (bHLH) domain, Achaete-scute transcription	10.92	2.57	2.09	0.00017
Nitab4.5_0000156g0090	Cytochrome P450, E-class, group I, Cytochrome P450	8.49	1.98	2.10	0.00033

Nitab4.5_0001342g0100		17.58	4.02	2.13	0.00000
Nitab4.5_0001972g0010	Aux/IAA-ARF-dimerisation	18.54	4.23	2.13	0.00000
Nitab4.5_0002607g0040	Photosystem I PsaA/PsaB	12.79	2.91	2.14	0.00813
Nitab4.5_0002200g0010	Polyphenol oxidase, Tyrosinase, Polyphenol oxidase, C-terminal, Uncharact	43.42	9.87	2.14	0.00046
Nitab4.5_0000457g0270	Fatty acid hydroxylase	28.15	6.39	2.14	0.00000
Nitab4.5_0000137g0250	Lipase, GDSL, SGNH hydrolase-type esterase domain	22.18	5.04	2.14	0.00000
Nitab4.5_0006560g0010	SANT/Myb domain, Myb domain, Homeodomain-like, Myb domain, plants	37.27	8.42	2.15	0.00000
Nitab4.5_0006665g0030	Carbonic anhydrase, Carbonic anhydrase, prokaryotic-like, conserved site	87.26	19.41	2.17	0.00000
Nitab4.5_0006485g0040	Glyceraldehyde 3-phosphate dehydrogenase, NAD(P) binding domain, Dom	118.04	26.06	2.18	0.00000
Nitab4.5_0001884g0050	Triose-phosphate transporter domain	98.04	21.52	2.19	0.00000
Nitab4.5_0003914g0070	Protein of unknown function DUF1624	7.52	1.64	2.20	0.00067
Nitab4.5_0000408g0160	Thioredoxin, Thioredoxin domain, Thioredoxin-like fold, Thioredoxin, conserv	26.49	5.76	2.20	0.00002
Nitab4.5_0001278g0120	Lipase, GDSL, SGNH hydrolase-type esterase domain	6.10	1.32	2.21	0.00288
Nitab4.5_0000007g0130	ATPase, F1 complex alpha/beta subunit, N-terminal domain, ATP synthase s	23.48	5.07	2.21	0.00002
Nitab4.5_0000008g0920	HAD-like domain, Phospholipid/glycerol acyltransferase	11.82	2.54	2.22	0.00001
Nitab4.5_0014018g0010	Cytochrome P450, E-class, group I, Cytochrome P450, conserved site, Cyto	14.02	3.00	2.23	0.00000
Nitab4.5_0007255g0060	Germin, manganese binding site, Cupin 1, Germin, RmlC-like cupin domain,	6.40	1.37	2.23	0.00064
Nitab4.5_0007223g0040	Acyl transferase/acyl hydrolase/lysophospholipase	5.67	1.20	2.24	0.00511
Nitab4.5_0001265g0130	Uncharacterised protein family Ycf49	5.38	1.14	2.25	0.00249
Nitab4.5_0004892g0060		10.33	2.14	2.27	0.01168
Nitab4.5_0025922g0020	Photosystem I PsaA/PsaB	43.29	8.93	2.28	0.01426
Nitab4.5_0014528g0010		84.57	17.43	2.28	0.00324
Nitab4.5_0009795g0010	Major intrinsic protein, Major intrinsic protein, conserved site, Aquaporin-like	23.28	4.78	2.28	0.00000
Nitab4.5_0017715g0010	Uncharacterised protein family UPF0497, trans-membrane plant subgroup, U	7.69	1.56	2.29	0.00019
Nitab4.5_0005255g0020	Proteinase inhibitor I20, Pin2	431.87	88.08	2.29	0.00000
Nitab4.5_0000397g0230	Cupredoxin, Plastocyanin-like	12.45	2.54	2.30	0.00052
Nitab4.5_0000005g0450	Multi antimicrobial extrusion protein	19.79	3.99	2.31	0.00263
Nitab4.5_0006857g0030	O-methyltransferase, family 2, Winged helix-turn-helix DNA-binding domain,	6.30	1.27	2.31	0.00023
Nitab4.5_0009011g0010	FAS1 domain	9.67	1.95	2.31	0.00038
Nitab4.5_0001160g0120	Bifunctional inhibitor/plant lipid transfer protein/seed storage helical domain	16.17	3.20	2.33	0.00028
Nitab4.5_0004288g0010		637.67	126.88	2.33	0.00000
Nitab4.5_0002073g0040	Conserved hypothetical protein CHP00245	8.94	1.77	2.34	0.00003
Nitab4.5_0000189g0170	Germin, manganese binding site, RmlC-like cupin domain, Cupin 1, RmlC-lik	21.73	4.27	2.35	0.00000
Nitab4.5_0003714g0050	AWPM-19-like	6.35	1.22	2.36	0.00087
Nitab4.5_0003531g0020	Bifunctional inhibitor/plant lipid transfer protein/seed storage helical domain,	27.05	5.26	2.36	0.00000
Nitab4.5_0009644g0020	Histone-fold, Histone H3	12.31	2.36	2.36	0.01063
Nitab4.5_0008409g0010	Cytochrome P450, Cytochrome P450, E-class, group I, Cytochrome P450, c	28.44	5.51	2.37	0.00000
Nitab4.5_0001668g0120	Heavy metal-associated domain, HMA	68.85	12.94	2.41	0.00000
Nitab4.5_0000291g0010		8.58	1.60	2.41	0.00192

Nitab4.5_0001023g0020		9.71	1.79	2.42	0.00048
Nitab4.5_0003142g0020	Drug/metabolite transporter	7.92	1.48	2.42	0.00227
Nitab4.5_0000101g0270	Phenylalanine ammonia-lyase, Fumarase/histidase, N-terminal, Aromatic am	53.67	9.88	2.44	0.00000
Nitab4.5_0000555g0100		9.14	1.64	2.48	0.00000
Nitab4.5_0000689g0050	Cytochrome P450, Cytochrome P450, E-class, group I	8.70	1.56	2.48	0.00001
Nitab4.5_0000032g0030	Histone H3, Histone core, Histone-fold	8.39	1.50	2.48	0.00001
Nitab4.5_0009642g0010		38.88	6.91	2.49	0.00000
Nitab4.5_0000037g0290		153.75	27.26	2.50	0.01203
Nitab4.5_0000138g0160	ATPase, V1 complex, subunit F, ATPase, V1 complex, subunit F, eukaryotic	17.07	2.99	2.50	0.00026
Nitab4.5_0011729g0010	SANT domain, Homeodomain-like, SANT/Myb domain	105.83	18.62	2.51	0.00000
Nitab4.5_0001202g0030	Caffeate O-methyltransferase (COMT) family, Plant methyltransferase dimer	8.88	1.55	2.52	0.00037
Nitab4.5_0004623g0040	Expansin/pollen allergen, DPBB domain, Expansin, cellulose-binding-like do	4.17	0.73	2.52	0.00387
Nitab4.5_0008210g0050	Zinc finger, RING-type, Zinc finger, RING/FYVE/PHD-type	114.99	19.85	2.53	0.00000
Nitab4.5_0002733g0030	Terpenoid cyclases/protein prenyltransferase alpha-alpha toroid, Terpene sy	6.20	1.04	2.55	0.00067
Nitab4.5_0016854g0010	Pyrimidine 5'-nucleotidase, eukaryotic, HAD-like domain	3.20	0.52	2.57	0.00281
Nitab4.5_0006480g0020	Tyrosinase, Polyphenol oxidase, central domain, Polyphenol oxidase, C-tern	22.30	3.71	2.59	0.00009
Nitab4.5_0001759g0030	Homeodomain-like, SANT/Myb domain, SANT domain	10.85	1.75	2.61	0.00143
Nitab4.5_0010110g0020	Peptidase T2, asparaginase 2	24.85	3.99	2.64	0.00000
Nitab4.5_0002637g0060	Cyanobacterial aminoacyl-tRNA synthetase, CAAD domain	8.82	1.41	2.65	0.00314
Nitab4.5_0001567g0060	Alcohol dehydrogenase, C-terminal, Alcohol dehydrogenase GroES-like, Grc	6.38	0.98	2.68	0.00589
Nitab4.5_0002829g0040	Cytochrome b5-like heme/steroid binding domain, Fatty acid/sphingolipid de:	6.02	0.93	2.70	0.00007
Nitab4.5_0000374g0140	Bifunctional inhibitor/plant lipid transfer protein/seed storage helical domain,	10.59	1.63	2.70	0.00010
Nitab4.5_0000858g0060	UDP-glucuronosyl/UDP-glucosyltransferase	7.95	1.20	2.71	0.00008
Nitab4.5_0015407g0010	Cytochrome P450, E-class, group IV, Cytochrome P450	18.89	2.87	2.72	0.00000
Nitab4.5_0001418g0040		6832.29	1031.78	2.73	0.00000
Nitab4.5_0009018g0020		206.75	30.49	2.76	0.00000
Nitab4.5_0000604g0070		46.08	6.74	2.77	0.00000
Nitab4.5_0003636g0050	DNA-binding domain, AP2/ERF domain	5.40	0.75	2.79	0.00054
Nitab4.5_0000671g0120		9.31	1.33	2.79	0.00342
Nitab4.5_0000080g0030	Peptidase S24/S26, beta-ribbon domain, Peptidase S24/S26A/S26B, Peptid	3.92	0.53	2.84	0.00405
Nitab4.5_0016001g0010		45.44	6.29	2.85	0.01468
Nitab4.5_0000525g0290	Proteinase inhibitor I13, potato inhibitor I	1586.26	216.69	2.87	0.00000
Nitab4.5_0004573g0070	Uncharacterised protein family Ycf2	23.05	3.14	2.88	0.04150
Nitab4.5_0001769g0050	DNA polymerase delta, subunit 4	2.67	0.33	2.91	0.00600
Nitab4.5_0003280g0010	Alanine racemase/group IV decarboxylase, C-terminal, Orn/DAP/Arg decarb	27.22	3.57	2.93	0.00001
Nitab4.5_0008240g0010	NAD(P)-binding domain, Short-chain dehydrogenase/reductase SDR, Gluco:	22.83	2.99	2.93	0.00000
Nitab4.5_0002660g0010	Multi antimicrobial extrusion protein	4.45	0.58	2.94	0.00352
Nitab4.5_0012950g0010	P-loop containing nucleoside triphosphate hydrolase, Sulfotransferase doma	7.86	1.00	2.98	0.00285
Nitab4.5_0006525g0010	Phloem protein 2-like	318.32	40.36	2.98	0.00000

Nitab4.5_0001146g0080		27.17	3.40	3.00	0.00000
Nitab4.5_0001884g0060		9.28	1.12	3.03	0.00012
Nitab4.5_0007471g0020		10.98	1.31	3.04	0.00082
Nitab4.5_0000407g0110	Glyceraldehyde/Erythrose phosphate dehydrogenase family, Glyceraldehyde	87.41	10.28	3.09	0.00000
Nitab4.5_0021312g0010	Terpenoid cyclases/protein prenyltransferase alpha-alpha toroid	8.14	0.93	3.11	0.00000
Nitab4.5_0006794g0070		4.89	0.54	3.11	0.00061
Nitab4.5_0000102g0010	Class II glutamine amidotransferase domain, Asparagine synthase, Glutamir	30.74	3.54	3.12	0.00000
Nitab4.5_0002956g0070		13.51	1.48	3.16	0.00002
Nitab4.5_0001704g0090	UDP-glucuronosyl/UDP-glucosyltransferase	65.74	7.29	3.17	0.00000
Nitab4.5_0000156g0120	Cytochrome P450, Cytochrome P450, E-class, group I, Cytochrome P450, c	86.62	9.51	3.19	0.00000
Nitab4.5_0002737g0030	Ubiquinone biosynthesis O-methyltransferase, Methyltransferase type 11	4.03	0.40	3.29	0.00030
Nitab4.5_0000048g0020		3.05	0.27	3.30	0.00310
Nitab4.5_0008490g0020	Rossmann-like alpha/beta/alpha sandwich fold, Class II glutamine amidotrar	31.19	3.17	3.30	0.00000
Nitab4.5_0007439g0010	IQ motif, EF-hand binding site, P-loop containing nucleoside triphosphate hy	3.72	0.34	3.32	0.00072
Nitab4.5_0000101g0120	Aquaporin-like, Major intrinsic protein, Major intrinsic protein, conserved site	60.49	5.99	3.34	0.00000
Nitab4.5_0000218g0060	Chloramphenicol acetyltransferase-like domain, Transferase	20.80	2.05	3.34	0.00000
Nitab4.5_0001264g0180		29.59	2.85	3.36	0.00029
Nitab4.5_0000365g0010		37.92	3.66	3.37	0.00000
Nitab4.5_0000024g0270		14.50	1.38	3.38	0.00000
Nitab4.5_0003558g0030	Zinc finger, RanBP2-type	5.36	0.46	3.40	0.00007
Nitab4.5_0001003g0220	Phosphatidylethanolamine-binding protein PEBP	41.97	3.93	3.42	0.00000
Nitab4.5_0000850g0090	PGG domain	5.28	0.45	3.45	0.00007
Nitab4.5_0005940g0010	SWEET sugar transporter	29.28	2.60	3.50	0.00000
Nitab4.5_0000085g0070		3.33	0.25	3.51	0.00201
Nitab4.5_0010833g0010	Phytosulfokine	17.21	1.50	3.51	0.00000
Nitab4.5_0002794g0050	Poly(ADP-ribose) polymerase, catalytic domain	3.70	0.27	3.57	0.01365
Nitab4.5_0001402g0100		18.54	1.51	3.58	0.00000
Nitab4.5_0001146g0060		114.88	9.39	3.61	0.00000
Nitab4.5_0000892g0100	Zinc finger, CCCH-type	22.85	1.81	3.64	0.00000
Nitab4.5_0002611g0090	Photosystem II PsbL	18.90	1.45	3.66	0.01456
Nitab4.5_0005948g0010	Auxin efflux carrier	22.70	1.76	3.68	0.00000
Nitab4.5_0002910g0020		3.34	0.21	3.70	0.01022
Nitab4.5_0002519g0020		11.92	0.87	3.73	0.00002
Nitab4.5_0000900g0150	Carboxypeptidase A inhibitor-like	172.50	12.92	3.74	0.00000
Nitab4.5_0000038g0040		20.37	1.52	3.74	0.00000
Nitab4.5_0019241g0010	Cellulose synthase	5.10	0.32	3.82	0.00019
Nitab4.5_0002590g0030	Chloramphenicol acetyltransferase-like domain, Transferase	28.14	1.97	3.83	0.00000
Nitab4.5_0006880g0040	Myb domain, Homeodomain-like, SANT/Myb domain	4.41	0.29	3.85	0.00028
Nitab4.5_0001574g0130	Nucleoside phosphatase GDA1/CD39	51.59	3.56	3.86	0.00000

Nitab4.5_0003113g0100	SWEET sugar transporter	3.49	0.22	3.90	0.00281
Nitab4.5_0002788g0080	ATPase, V1/A1 complex, subunit E	8.91	0.55	3.91	0.01595
Nitab4.5_0003024g0020	SGNH hydrolase-type esterase domain, Lipase, GDSL	9.81	0.65	3.92	0.00000
Nitab4.5_0001199g0090	RmlC-like cupin domain, Cupin 1, Germin, RmlC-like jelly roll fold	10.79	0.70	3.92	0.00000
Nitab4.5_0000327g0140	Dehydroquinase class I, Aldolase-type TIM barrel	5.18	0.29	3.95	0.00835
Nitab4.5_0003558g0090	Zinc finger, RanBP2-type	4.93	0.28	4.07	0.00006
Nitab4.5_0000163g0290		5.71	0.30	4.07	0.00348
Nitab4.5_0018794g0010	SANT/Myb domain, Homeodomain-like, Myb domain	4.16	0.21	4.14	0.00130
Nitab4.5_0011473g0020	Protein of unknown function DUF1442	6.00	0.30	4.15	0.00545
Nitab4.5_0001777g0030	Knottin, scorpion toxin-like, Gamma thionin	10.10	0.51	4.18	0.00000
Nitab4.5_0001989g0050	Lipase, GDSL, SGNH hydrolase-type esterase domain	3.20	0.15	4.20	0.00067
Nitab4.5_0005563g0020	Zinc finger, RanBP2-type	37.02	2.00	4.21	0.00000
Nitab4.5_0000425g0230	Proteinase inhibitor I3, Kunitz legume, Kunitz inhibitor ST1-like	17.43	0.91	4.26	0.00003
Nitab4.5_0002482g0070	Uncharacterised domain, di-copper centre, Tyrosinase, Polyphenol oxidase,	87.75	4.27	4.36	0.00000
Nitab4.5_0000129g0260		78.29	3.75	4.38	0.00000
Nitab4.5_0000434g0190		6.67	0.28	4.39	0.00000
Nitab4.5_0005563g0030	Zinc finger, RanBP2-type	58.80	2.72	4.42	0.00000
Nitab4.5_0000023g0480	Proteinase inhibitor I3, Kunitz legume, Kunitz inhibitor ST1-like	65.50	3.03	4.43	0.00000
Nitab4.5_0000460g0030	Photosynthetic reaction centre, L/M, Ribosomal protein L10e/L16	12.75	0.54	4.48	0.00103
Nitab4.5_0005563g0040	Zinc finger, RanBP2-type	12.90	0.54	4.53	0.00000
Nitab4.5_0000072g0610	Pectinesterase inhibitor domain	6.19	0.21	4.61	0.00507
Nitab4.5_0001833g0060	Terpenoid synthase, Terpene synthase, N-terminal domain, Terpene syntha	38.64	1.43	4.76	0.00000
Nitab4.5_0004531g0020	Homeodomain-like, SANT/Myb domain, SANT domain	8.90	0.28	4.80	0.00000
Nitab4.5_0004981g0040		17.65	0.37	5.43	0.00099
Nitab4.5_0000218g0070	Chloramphenicol acetyltransferase-like domain, Transferase	10.59	0.22	5.48	0.00026
Nitab4.5_0004288g0030		5.60	0.06	5.80	0.00180
Nitab4.5_0006875g0020	Probable transposase, Ptta/En/Spm, plant	3.29	0.00	5.81	0.00067
Nitab4.5_0001903g0040		3.84	0.00	6.03	0.00008
Nitab4.5_0000470g0130	Probable transposase, Ptta/En/Spm, plant	3.94	0.00	6.07	0.00107
Nitab4.5_0005345g0060		3.95	0.00	6.07	0.00137
Nitab4.5_0000783g0020		4.23	0.00	6.17	0.00005
Nitab4.5_0011271g0060	Auxin-induced protein, ARG7	4.52	0.00	6.28	0.00319
Nitab4.5_0009643g0020	Protein of unknown function DUF761, plant	4.71	0.00	6.33	0.00001
Nitab4.5_0001049g0030		5.19	0.00	6.47	0.00006
Nitab4.5_0008177g0050		5.18	0.00	6.47	0.00141
Nitab4.5_0003580g0050	Protein of unknown function DUF4228, plant	5.23	0.00	6.48	0.00000
Nitab4.5_0000472g0090		5.44	0.00	6.54	0.00158
Nitab4.5_0000308g0160	Homeodomain-like, Myb-like domain, SANT/Myb domain	5.79	0.00	6.63	0.00136
Nitab4.5_0006409g0060	NADH-quinone oxidoreductase, subunit D	6.53	0.00	6.80	0.00500

Nitab4.5_0007738g0080		6.86	0.00	6.88	0.00306
Nitab4.5_0003558g0070	Zinc finger, RanBP2-type	26.45	0.17	6.92	0.00000
Nitab4.5_0001492g0040	O-methyltransferase, family 3	7.88	0.00	7.07	0.00000
Nitab4.5_0005388g0110		8.56	0.00	7.19	0.00036
Nitab4.5_0001087g0080		8.94	0.00	7.26	0.00220
Nitab4.5_0000694g0130		10.09	0.00	7.43	0.00063
Nitab4.5_0010914g0020		11.82	0.00	7.66	0.00000
Nitab4.5_0001227g0060	Zinc finger, GRF-type	13.08	0.00	7.80	0.00000
Nitab4.5_0000907g0090	Tyrosinase, Uncharacterised domain, di-copper centre, Polyphenol oxidase,	104.33	0.36	8.10	0.00000
Nitab4.5_0003171g0010	Polyphenol oxidase, C-terminal, Tyrosinase, Uncharacterised domain, di-cop	165.37	0.57	8.15	0.00000
