# Supplementary material for: Horizontally Transferred Salivary Protein Promotes Insect Feeding by Suppressing Ferredoxin-Mediated Plant Defenses
Source: Mol Biol Evol. 2023 Oct 7;40(10):msad221. doi: 10.1093/molbev/msad221 (PMC10583550; doi:10.1093/molbev/msad221)
Supplement: msad221_Supplementary_Data [file msad221_supplementary_data.zip › Supplementary Data 1.docx]

>QHB15613 BtFTSP1 [Bemisia tabaci]

MHPRTIIRVILPLFLILLMRSHSISGKDEGKGGKKGGGKGKNDTNPDSGGKGKNDTDPNSGGKGGPKDLSIPDKCGKDNGMVPKPCTYSYSGCKLKGSKLNFKTVSNDMIQMGECWANTRVLGQRVAFFVPVSPSVKNYGSEGGPYGSCHAYAGCPTADELEASDNPAEFCFIWETNPNGGGNKKKGNNGTDTCNPKCPGSPALPMPIVCPSTLKCCYSADWYEKGGKLEKYGNLVPTYKGVWFPLKGRKDHKPLFCPGLNLGCPTPTYRDLQLHHLEERKCDVSKCKGGGSPA

>XP_018909720 BtFTSP2 [Bemisia tabaci]

MKITLFLALPVLLVICSVVWARDKGRKEAVNSQFNPKKPKPGGKPSGKPNKKPDDGMFPHPAILAYSGCKLRTKSLPCNSKGHPHIQMGEAWANPRVLNQRVAFFTPYKSSHPQFCNQGGPYGDVIAYDCLPKKEDLVYTPNLASYCFLWEAGINGKTDNNGTMPDPPKGSYHALPPPMVCPSNLDPCYCKDWFKPGGKLGKYGNYKIGYKGLWCPPKTKEKPMKPLFCGGFDLGCKPGTYLEMQTEFLPKLNCTVSKCKTPKPLAEV

>CAH0381103 BtFTSP3 [Bemisia tabaci]

MNFAKHMIVVTLLVGIETTFGQNGDPHPAASSFSGCKIKWPLNGGCYAKLTVLVQAGQCWANTRIANQKSAYFVPVPPTTPGYNAQGGPYGICTGCNWVPKATDLVASNNPADFCFFWHDAAHPVLAPGKPTIPMPIICPSNLNPCPSLNWYEPGNKLGMYADWKSAYEGVWFGPRDKPPSYKALYCNGLNLGCETLTNRDFQFPRIVGKCDVTKCRPRS

>XP_018907192 BtFTSP4 [Bemisia tabaci]

MEVAVSVALLILANLWLTVIPQTKKDDKHPRPANASYSGCRLTIGKLTGANSESNDGIQMGECWANTRVHNQKCAFFVPFEPGHEHYDSEGGPYGSCLALDQYPQASDMSETSNLANWCFFWETKVDPSSNATAAPLPSAPGEKGLPMPIVCPSNLKDCYSADWFEPGGKLGAYGDLYPTYGGIWFPLKDPNTRKPLFCQGLGLGCDTPNMRGLQLQHLINGTCDVEHKCHPPNGTNPKP

>NODE_30271 SsFTSP1 [Singhiella simplex]

MRISLFTTFVALSVHSSSADKSPNPAVVSYSGCGLANGLPKCCTAKKTNVHIQMGEAWANPRVMNQRFAVFVPYKPGEKSYSNEGGPYGDVTACNCYPTEKEMKKVDNLANWCFFWDGQDHTLKPPSDSGSDSGSDSGSGSGSKKVRRASYEKSGKGEKGEKGGEGGKGEKGGEGGNGGKGGEGGKGGKGGKGGKGGEGGEGGEGGEGGSGKGNSTIPNAPGMKAIPPPIVCPSTLNGCYSASYYEPGGKLEVWANVKPTYKGVWFPPKDKSTHKPLFCEGFNLGCPTPTMRMLQLQNLPKVKCDPSNCQANPNGGSGGGGGGGSGNEPGNGGGSGGGGKGGKGGESGNGGNSGQGGEPGGGGKGGKGKKKSNDNNGNA

>NODE_19893 SsFTSP2 [Singhiella simplex]

MKVILSIAFVALSVYTSIADKSPNPAVVSYSGCGLKNGVPKCCTNKKTDVDIQMGEAWSNLRVMNQHFAVFVPYKPGTKSYDSEGGPYGDVTACNCYPTESEMVKVDNLANWCFFWDGKDHTGSGSGSSSGSSSGWVIPHAPGEKATPMPIVCPSNLPNCYSANYYEPGGKLAAYANVKPTYRGIWFPLQDKSSRKPLFCNGLNLGCPTPTMRMLQLQHLAKANCDPSRCQKHPSAG

>NODE_110072 SsFTSP3 [Singhiella simplex]

MVPIPASHSYSGCHLKSGSVSGAQSKANTYIQMGECWANTRILHQRSAFFVPVPPSDPKYGMEGGPYGTCTAWDAMPAASDMEKSSNLAEWCFFWDKDPNGGGGGGGGGGGGGCSGGGGEGGGGEGGGGEGGGGESDGGEGGNKQKKKSDGSKKPKKPCSGGGGKQSSSSGVPSAPGEKAIPMPIVCPHNLIGCYSADWFEPNGKLAKYGSMVPTYKGVWFPLKDASTKENLFCNGLGL

>NODE_4789 SsFTSP4 [Singhiella simplex]

MTSVGFIDSAIMKMAIPIAFVVLLTTSFLVDGRKDEGMFPSPAKGTYSGCKLKQGILKGCALKANTNIQMGECWSNPRVLRQKFAVFVPFEPGQQNYGSEGGPYGTCTAFDCWPKQEDMEKVGNPANWCFVWDGTLNGGDKSKKKKSRRSPKKSSSDSPKSPSDSSKSPSDSSKSPSDSSKSPSDSSASGSGAEMPSSCPGEKAIPMPIVCPSNLKDCYSADWYEPGGKLAAFGSMKPTYKGPWFSLKGPDHKPLFCPGLNLGCPTPTMRELQLLHLADQKCDVASCKGGSGGGSGGGSDSSDGSSSPKNPKKKKN

>NODE_29096 DcFTSP1 [Dialeurodes citri]

MKEALPITLVILLAYFSFVNGQSGVVPAVSFWSKCRLKLTVLKGCFTKTNVGVQMGQCWANQRINGQKSAYFIPFKPGTPQYDFEGGPYGICTACDWFPNPVTDLVVSDNLAEFCFFWDDKANPVKAPGEIAVPMPVICPSNLPNCPSAKWFEPGGPLATLATWKPTYKGVWFPPKDLVSKKPLFCQGLDLGCVTPTMLDLQLNRLAGMKCDITKCKAPIG

>NODE_20410 DcFTSP2 [Dialeurodes citri]

MKLAFPIAFVVLLAYFTIVNSQSGIVPANSFWSKCRLKWNILRGCYSKVNVGIQMGQCWANQRINGQKSAYFIPFKPGTPQYDSEGGPYGICTACDWLPNPATDLAVSDNLADFCFFWDDKANPVKAPGEIAVPMPVICPSNLANCPSANWFEPGGPLAALATWKPTYKGIWFPPKNPMKKPMFCQGLNLGCDTPTMRDLQLARLAGMKCDLTKCKAPFG

>NODE_6494 DcFTSP3 [Dialeurodes citri]

MTVFPIVFAVLLGNSFVSGGGRKDEGMVPIPASVSYSGCQLANGKIAGLKSKGNTYIQMGECWANTRIMNQRSAFFVPVDKSDPKYTAEGGPYGTCMAWDGFPTDSELAKSDDPAHWCFFWEKNPNGGGGGGGCSSGGGGGGGGGGGGGKNGGGGKDGGGGKNGGGGKNGGGGKNGGGGKNGGRNRRGGDGSKKKKKSSEEDPDKSTKDPDNSTKDPDNSTKDPDNSTKDPNNSSKCGGMPKNIPGVKAIPMPIVCPSNLQDCYSADWFDAGGKLASYGSLVPTYKGVWYPPKDPTKHTPLFCQGLSLGCKTPTMRQLQLHQLPKQGCDLASCKGGSSGGESGEKESGKKKKKP

>NODE_28436 AsFTSP1 [Aleurocanthus spiniferus]

MMPLPNMILALVLAFAIALTEARKDDGMVPHPAEISFQKCKMKGSSLSGCQSKTDNMIQQGECWSNPRVLRQKSAFFIPTPNKQHYDAEGGPYGDCIAFDCWPKESDLEEAPDYSYWCFFWDGTLNCEDGCGGDKNKKQSSSGGNSTDTGNGGGMPSNAPGEKGIPMPIVCPSNLVDCYSADWFDPGGKLEKYGSLTPTYKGVWYPLKDKSSHGPIYCNGLDLGCKTVTMREFQLHNLPKAGCDVKSCKSSAAGGGGGGGGGGGGSNSTDSGGGGGGGSGSLNSTDSGGGGANVTNSLSESKN

>NODE_18108 AsFTSP2 [Aleurocanthus spiniferus]

MISPPSMIAALILTFVVALTEARKDAGMVPHPAEISFQKCKMKGTTLSGCQTKTNNMIQQGECWANPRVLRQKSAFFIPTPSNKYYSAEGGPYGDCIAFDCWPQISDLEEAPDYSYWCFFWDGTLNCEGGCGSGSGGAKKKKKSGKGDGDGSGGNSTDTGDGGGGGGGGSMPTTAPGEKGIPMPIVCPSTLNDCYSADWFDPGGKLEKYGSLKPTYKGVWFPLKDKSTHEPLFCQGLNLGCETVTMRDLQLHTLPNAGCSVSSCKGGSGGGGGGDGEGGCPSSSSSSSSSSSKKKKSSSGGSSSCSKSPSSPPGPSGKSPDKSPSKSPDKSPSKSPDKSPKSPSKPPKNPPSKSPPAEERRRK

>NODE_89821 AsFTSP3 [Aleurocanthus spiniferus]

MEVTFSFPVVFAVLLASSCVVDGNDWPASNSFSGCRVKWPLKCASNASPNVQMGQCWGNQRVNNQKSAFFVPVEPTAQEYSFEGGPYGMCTAFDCYPAITDMEPFTNPANWCFFWDDKAAPIKAPGVPAVPMPIICPSVLQNCPSALWYEPGNKLSAYSDWKPTYKGIWFPPKNPLTKQPMFCQGLDLGCPTPTNRDLQLLRLAMQNCDITKCKPPPPMG

>NODE_89822 AsFTSP4 [Aleurocanthus spiniferus]

MNAFPLAIPVLLARFCLVNGNDWPAAHSYSGCRAKWALKCNSKVSKNVQMGQCWALQQVNNQKSAFFVPVPTTAPEYTAEGGPYGYCTVFDCYPAPWDMEYSASLTYWCMFWDDQAKPIQAFGIAAIPMPIICPSNLQNCPSAFWYEPGNKLAAYGGWKPTYKGIWFPPKNPWTKQPMFCQGLDLGCGTPTNRDLQLHRLALMKCDVSKCKPPPNQDPELCRTKRVQC

>NODE_063420 ApFTSP1 [Aleuroclava psidii]

MCVTRGSVWELRHFVLIFRYILDGVRPSVIMMTVFPIVFTVLLGNSFVSGGRKDEGMVPKPASVSYSGCQLANGKIAGLKSKGSNYIQMGECWANTRIMNQRSAYFIPVDASDPRYDNEGGPYGTCMAWDGFPTESELAKSDDLAHWCFFWEKNPNGGGGGGGCSSGGGGGGGKNGGGGKDGGGGKDGGGGKNGGGGKNGGGGKNGGGGKNGGGGKNGGGGKNGGGGKNGGGDKSGGGDKNGGRDRRGGDGSKKRKKGSETQDEDPETSTKDPETSTKDPETSTKDPDNSSKNPDNSSKCGGMPKNIPGTKAIPMPIVCPSTLTDCYSADWYDAGGKLASFGSLVPTYMGPWFPPKDPQKRTPLFCQGLSLGCKTPTMRQLQLHQLPKQGCDIESCKDPSGAKESGKKKKKP

>NODE_032806 ApFTSP2 [Aleuroclava psidii]

MKEALPITLVILLAYFSVVNGQSGVVPAVSSWSKCRLKLTVLKGCFTKTNVGVQMGQCWANQRINGQKSAYFIPFKPGTPQYDFEGGPYGICTACDWFPNPVADLVVSDNLAEFCFFWDDKANPVKAPGEIAVPMPVICPSNMPNCPSAKWFEPGGPLAILATWKPTYKGVWFPPKDLVTKKPLFCQGLDLGCDTPTMLDLQLNRLAGMKCDVTKCKKPIG

>NODE_126363 ApFTSP3 [Aleuroclava psidii]

MKLAFPIAFVVLLAYFTIVNGQSGIVPANSFWSKCRLKWNILRGCYSKVNVGIQMGQCWANQRINGQKSAYFIPFKPGTPQYDSEGGPYGICTACDWLPNPATDLAASDNLADFCFFWDDKANPVKAPGEIAVPMPVICPSNLANCPSANWFEPGGPLASFASWKPTYKGIWFPPKNAAKNPMFCQGLNLGCDTPTMRDLQLAHLAGMKCDVTKCKKPIG

>NODE_016320 ApFTSP4 [Aleuroclava psidii]

GDDLAGLGFGPHPAFDAYSGCRLKWGPSIKGCTCTSSSGIQMGETWANIRVQRQLSAFFVPFQPGEKNYGSEGGPYGTGTGCNCWPKEWDMEYSNNPADMCFFWKNDYNGENGNSTESDMPKFTAGERFVPMPITCPSILKNCYAEYWYEAGGALAAYATWKPTYKGVWFPPKDPSSHRPLFCEGLNLGCPTPTNQYLTLHRLPGVCDVSRCRPSPYRNSTV

>NODE_268628 ApFTSP5 [Aleuroclava psidii]

MKVTLLIAIVISLVISAVIGGRPRPAFVSYSGCRPKSGSTYKGCKCTTNTGIQIGETWANTRVQGQLSGFFISYKPGEKNYDSEGGPYGDGTGCDCYPTNAEMVKSENLADFCFFWKPADNTGKGGKSGKGGKNKGKGTNSTNSMPTVIPGEEVIPPCLVCPHNLNPCYSANYFEKGGPLAGYASQRPTYKGPWFPPKDPKSKKPLFCQGFNLGCPAPTYRDLQLHRLPGVNCDVSKCERPTTTG

>NODE_211042 ApFTSP6 [Aleuroclava psidii]

GGHPRPAFVSYSGCRLKSGPTIKGCKCVTNTGIQMGETWANTRVQGQLSGFFVSYKPGEKNYGSEGGPYGDGTGCDCHPKNAEMVKSENLADFCFFWKPAPGKDVQSKGNDTNSTMPTVIPGEEVIPMPIVCPHNLKNCYSANYYEPDGPLAEYASQKPTYRGPWFPLKDPSTEKPLFCQGLNLGCPTPTNRDLQLHRLPGVKCDVSKCKPPTTTV

>VMOF01000320.1 38542892-38543752 TvFTSP3 [Trialeurodes vaporariorum]

MRVALTMAFAVLLVECFETPPKSKDETNPVESPVESSDGKQPQQKKEIQPYPAILSYSGCKLKKGALSKCKTSSNKDIQMGEAWANPRVHGQVFAVFVPKKPGEGNYASQGGPYGLCAAWDCHPTEKDLEPSKNPAAYCFFWDPKDTGRPWPEGCDPKIPPPDPCIKPDKNKKPDKKPGGGGGGGGKPSTCPAPRPTKIPGEPGFPMPVVCPSNLKPCYSAAWYQPGGKLAKYADCKPTYKGQWFPLKENGTWKPLYCEGLNLGCGSPTNFELQLQYLLKKKCDVSTCKKPPPMPPV

>VMOF01000320.1 38547295-38548146 TvFTSP2 [Trialeurodes vaporariorum]

MRLALATVFVVFLVNCCDGNNGKGGNRPRPAIVSYQGCKVNESALKKCASVSNKGIQMGEAWANPRVHGQLFAVFVPNEPGVGNYASEGGPYGYVTAWDCNPLEADLSASDNYANWCMFWDGKDTGRKETGCSETPSSGDNTNPSPDTSEPAESDPKKPKKPDPKKPDPKKPGGGCPNPIPSKVPGEKGLPMPIVCPSNLNPCYSAKFYDPGGPLAKYASMKPTYKGIWFPLKNKATRNPLFCMGLDLGCPTPTMLQLQLHHLPDVGCDVSKCKVPPGAPAPK

>VMOF01000320.1 53589052-53588000 TvFTSP1 [Trialeurodes vaporariorum]

MRLAVATVFVVFLVNCCDGNNGKGGNTPRPAIVSYQGCKVNESALKKCASVSNKGIQMGEAWANPRVHGQLFAVFVPNEPGVGNYASEGGPYGYVTAWDCNPLEADLSASDNYANWCMFWDGKDTGRKPTTNCGGTPSPSPGDNTNPSPDNTKPKKPKKPDPKKPDPKKPDPKKPGGGCPNPIPSKVPGEKGLPMPIVCPSNLNPCYSAKFYDPGGPLAKYASMKPTYKGIWFPLKNKATRNPLFCMGLDLGCPTPTMLQLQLHHLPDVGCDVSKCKVPPGAPAPK

>CP096878.1 [Exobasidium cylindrosporum]

MKFAASRSQLMPLLLLAGSAIASPFNTRINYQQLAKRDPSGVPADILTEGCALRNTNFASHHSDHAIQIGECYVNSRYVKRKAVSVAITFVSRINLFLMAELGQRFCTFEPVDGKMRSPREGGPYGEWTSSTTVLEQVSSHLLLFIQGTCSCYASFPEAKDLMAFENPNYACLAWPTKTPALTAPEMPGKACIPFPLVCPTTLKGCYAEQYFTPQGPYVSFWFYAFRGSNPSTDALLLCS

>JAAVVH010000005.1 [Golubevia sp. BC0812]

MNLTRSLVLSILGLCSQVSTSTLHHLHRRDASPFPSDIAHSGCAVSSSWFNNPTWTDSKGEKIKCGRKVRYIVERKLEVVFLEGCSIDLFPFPSFVLRPVRVSRWENALLITGSLTIYLRSGEFISFEAAQICAFNWTHPQPLLRFPYLPTDAHYTSEGGPYGFCSAFDCQPLASDLTSENNPSNACFFWSYKENRPNLPKSIPGVDFVPPPMICPHNLEGCYSKTYYDKGGKVSERSRARNYVVSFDTGCSNFPLFCLMNSLVTSEI

>JAAVVG010000009.1 [Golubevia sp. BC0850]

MNLTRSLVLSILGLCSQVSTSTLHHLHRRDASPFPSDIAHSGCAVSSSWFNNPTWTDSKGEKIKCGRKVRLSSKGSFEVVFLEGCSIDLFPFPSFVLRPVRVSRWENALLITGSLTIYLRSGEFISFEAAQICAFNWTHPQPLLRFPYLPTDAHYTSEGGPYGFCSAFDCQPLASDLTSENNPSNACFFWSYKENRPNLPKSIPGVDFVPPPMICPHNLEGCYSKTYYDKGGKVSERSRARNYVVSFDTGCSNFPLFCLMNSLVTSEI

>JAAVVI010000001.1 [Golubevia sp. BC0902]

MNLTRSLVLSILGLCSQVSTSTLHHLHRRDASPFPSDIAHSGCAVNSSWFNNPTWTDSKGEKIKCGRKVRYIVERKQVVFLEGCSIDLFPFPSFVLRPVRVSRWENALLITGSLTIYLRSGEFISFEAAQIRAFNWTHPQPLLRFPYLPTDAHYTSEGGPYGFCSAFDCQPLASDLTSENNPSNACFFWSYKENRPNLPKSIPGVEFVPPPMICPHNLEGCYSKTYYDKGGKVSERSRARNYVVSFDTGCSNFPLFCLMNSLVTSEI

>BCHO01000002.1 [Tilletiopsis pallescens]

MRSLSFLSLLPLFFLSLEVILAFPANEHPHQLEKRDASPFPADKAFAGCSVSTAWFNAPTWRNGAGRVTRCGRKTDTDVQMGECGSNDRIQNNVFAIFVGQETNKRDEKHSRASTADTFFFHSISIPFTKVPYLPGDPHYDSEGGPYGYCSAFDCQPLATDLTKEANPSNACFLWSYKEDRPAVPNEIPGESFVPPPMICPHNLPDCYSKNYYDRGGKVSIKTPKWIEEAFEV

>UWYS01000030.1 [Thecaphora thlaspeos]

MRCPSSGLALLFATLEVAMLAQAHFSYSPRVRRGLHESIVHHKRDVAPFPSDLVRNQCNVDPDWLQNPWYFEPKTSQKMVCNFVMNKHIQKSECTTNSRVSLPSLPTRACSRESNGLDTRTLRLSFILPRLCPYVQPLLQCRVINNVFAAFVPYTLDDKVAKNEGGVYGDCFGFDCGPPAEVLREAPNDNFACAWWMDGGKVRYPLIPPKLPGYTAILAPIACPSTNGCYSQNYYEPGGKLAQLANVKMETCEIWFPVKDKSHKETWCHGANLGCETPTYAQLVRGESERSSLDLLFCPSASRPSSPCGRTRSLTSFPPFAYSAGCDTSKCKEHPGLTSSAWKFDNCDYNPVYDGCSVKKTGWGNSDSVACSGGGGGGGGNKLEKRGSTLSTKCAAVPKLSGAPASIASYVQGTPDTKEKVLAMFPFTGCKSAGSFGTS

>BCJU01000001.1 [Meira nashicola]

MTSPSLLLLVMLAVTGMLIKADKSPNPAAISYSGCSLKQPLNCNSHSDSDIQMGECWSNARVMKQKSAFFVPFKPGEKSYHSEGGPYGTCTAYACFPSESDMQEESNLANFCFFWDGKDHTVLQNGGLSRRGLFERDGTASDDGDPSTSQNMTATTADPTTADPTTADPTTADPTTADPTTADPTTADPTTTDPTTTNSTTTDPTTADPTTTDPTAVDPSTGTAPDPTMGNNATSTGTKGNNKYKHKKMNKIIKTNPKKTNPKKTNPKKTNPKKTKTKNTKPKKTKTKKPKTKKPKTKKPKKNPPSGGGSGSQPSNSSMPNAPGKVVIPIPMICPSNLVNCYSANYFEKGGKLEVYANLVPTYTGLWFPPKNKNHEFMFCKGLGLGCPTPTMRNLQLQHIGDQHCDTSKCKVPPNS

>XP_025357528.1 uncharacterized protein FA14DRAFT_11843 [Meira miltonrushii]

MTSPLLLLFVMLAVTSIPIKADKSPNPAAISYSGCSLKQPLDCNSHSDDDIQMGECWSNSRVMQQKSAFFVPYPPGHQSYHSEGGPYGGCTAYACYPSEGDMQEEANLSNFCFFWDGKDHTVIPNGGLQRRGLAERDASASAAADPNASPNMTTTTPDPTTMGTTGTADPNAGATTDPNNVPATTSADPPADPTMNSSGTSKGKSKKIHKHKPKKMKKKKKHHGHRPTHKKHKDSTGTDSTGTPGGSGSDSTGTPANGSGTDATDPTTAGTMNNGTSTDSAAGTAPGPATDTTGTTGTGTATADPNGTGATDTGTTTDATAAPAADPTTGTGTTTDPTANTGSDATAPTGTGPTDTGTTTDTTAAPATDPTADSGNSAPDPNATGASSTGTTTDNTAAPAAGPTTDTGTGTTPDPTTNTGTDATAPTGTGPTDTGATTDNTAAPAADPTTDTSGANTSGTGVTAAVGGTNDDITASVSTGTGDPGNGSSESSATSSTGTASDRKKTKKTKKPKKVKKPKQAKKKKSPASGGGSSGGSEGGGSGSYTPPASTSSLPKAPGKVVIPIPMICPSNLENCYSANYFEPGGKLEVYANLVPTYEGLWFPPKHDHQFMFCKGLGLGCPTPTMRELQLQHIGDQHCDTSKCKVPPNSN

>CEH17835.1 hypothetical protein CBOM_04222 [Ceraceosorus bombacis]

MKLTSRLLFSALGLAALQLCLIDTVTGQTDPFGPAWDGGEHYIFSREEGMHILHKRDKSPDPAVATYQGCKLNDAFIAKHKHNSKTNEGIQMAECFSNCRISPNAFAYFLVDDSNIKREGYPYGTCTAFSVQPTIDDLAEGDPEMHMCGFWDRGGVMLEEAYGRSFVNMPMICPHNLKGCYSKDYYSPGGKLAEFANVKPTYDGHIWPPKDQDHELQYCECLNCGCPTPTYAEFQQIPIPGCDPKTMCKPYQGQKTGVEGANIRGKQAGTAKGTTGGSAGTGTAPTTGKSNGSGKGTTKGGNTSTGSGGSSNTGSGGNANTGSGSHKGHDNSADTGKKGSSSKKGSSTKQKESPSKGPDENEFPISGSESKSKDKDKKESSKDSKETTGASLDNTYKLGD

SGFDKIAGSSSAHGKGDDTSTVTENDLPDCEEDPKNSKASDATDTISNRDRSQKRSIGPDSASSVSSQTT

>UZJ51548.1 hypothetical protein CBS101457_000868 [Exobasidium rhododendri]

MSGLFAVSALGQTFLNSSNDTGTHQLVKRDPSYVQATRALQNCVLRDVNYHHRHDGGEKYCNSRIQMSECHVNSRVMNQVFAVFVPDTESPHFGEGGVYGDCCAYNVYPVDGDIWEWENPNYACFTWETQDKKQGLGPPKLPNTAMQPFPIVCPSNLHPCYSETVYNDAHQKMGKYYTLGVTFTGLWNPPKTGGPHGTEMYCNWIGGGCKSGNLRDYTLHYLPKLGCEPTTMCKHTYTQSTKGVNWSSVCPVGTFPPVSHFSFYANGGVPSAEKCKGVSSNGASSRGKGSYGQDGSGSSSGSGSGSGSGSGSSSGSSSGSSTGSGAGSGSHAASGKGSYGGKGQQCSENNPGGHHAGKGQHHAHDCKKAGSNCTPTHKCATNETRKSSLAERDVVPEGDSKERLENRSHKCHGQSTHRSSKRSDEEESDHSAFKRHIIKRYGKRKL

>UZJ51547.1 hypothetical protein CBS101457_000867 [Exobasidium rhododendri]

MHFGTSVTFLASMLLLSGSAMASITSFSTTSNSYYSLAKRDPSGVPADILTEGCAIKNPGYAANHTDHGIQLGECYINARIMKQMFCTFEPTDGDMKSSREGGPYGSCSCYNAFPKSTDLMSFENLNYACLAWTKKNAGYTPPKLPGEQCIPFPLVCPINLKGCYAKDYWTSSGPASLLPTANEQATCKMYWSPEKAFQGGKAIYCDHIQEACEVPTNLQFMQRNFTCDVSKCVPYQGSFKLLTPELRASGTNNGQSTTYIVPPPGGSNPVNPGGKGNSAGGTGSGSYGVDTIPTNQGTQGSNENRDKTATGSYGAAPLPNSPNSKSTQGAVKKSGKTSASGTATDTTTPANTNYQIGAGAVGSTAASGAGIGTTTPANTNYQIGAGSGGGVGGSTAASGTGVGTTTPAKTNYQIGAGAGGKSAATAAGVGTTTPTNTDTQAGTGVVKSSGKSGAVGSTNAGTTAVTNTPTGTNNDVNEADGKKKKSKDDDLEDCKDDSDDDKEKKKGKKEKKEKKEGVKDPSEESADVVDPVKTPVTKVAMVSRNHQIKSNRRRRL

>XP_007878543.1 uncharacterized protein PFL1_02838 [Pseudozyma flocculosa PF-1]

MRGPGFGPVVPLLAILAMAMQAHSHLTDSPFARRGLHASVIRSKRDVAPFPASLVRNKCVADIGWMAKPWAWEPTVKQNIACNVVVNADIQKAECMSNSRIMNNVFAVFVPNAVGDQSWRSEGGIYGMCYAYNCIPPAEVFAEAPNENFACGFWLDGEKPKYGYIPATLPGLTRTIEPIICPSKNHCYSQNWYDNGGKLHALASTVTDSCEVWFPVKDSQHKETWCHGVGLGCPTPTYLELVYGTNKCDVSQCREHPLLNTAAYAYKDCQYSPAAKGCTPPTTKWSNTAPDTSVCGSSGGHGMQADVKGYASQDCRTCYSQWKSTPDPKCYSVAKVANAAAGPPANFESYVLGNPENKQKVLGMFPKGPPCDWAKEAAGAGAGGAGGAAAGGAAGGAAGGAAGGAAGTGTGTGTGTGTGTGTGTGTGTGTGTGTDTGTDKGKGTGTDKGKGTGTDKGKGTGTDKGKKSAGEKSTAGSGSSKSSKEKSKPNDQTDAASGGDGGESAPIF

>MCO5562931.1 hypothetical protein L7F22_016567 [Adiantum nelumboides]

MLAVTGMLIKADKSPNPAAISYSGCSLKQPLNCNSHSDSDIQMGECWSNARVMKQKSAFFVPFKPGEKSYHSEGGPYGTCTAYACFPSESDMQEESNLANFCFFWDGKDHTVLQNGGLSRRGLFERDGTASDDGDPSTSQNMTATTADPTTADPTTADPTTADPTTADPTTADPTTADPTTADPTTADPTTADPTTADPTTADPTTTDPTTTNSTTTDPTTADPTTTDPTAVDPSTGTAPDPTMGNNATSTGTKVHHVASKLEYESIRYVFGVLFYCRKDNSFFSIFLPLKQLYTDKISGDEMFSDGYDFKEIDDVVYEVDAANIVVQEGDVDIGANPSAEEQQEALENGGQQVINIVHSFRLQSTTFDKKSYLTYLKGYMKAIKTQLQTDAPERVEVFEKKAQEFAKKVLGNFKDYDFYTGESGNPDGMVALLNFREDGVTPFLIFWKDGLKDVKI

>PWN49522.1 hypothetical protein IE53DRAFT_139363 [Violaceomyces palustris]

MRSNRALVLTLMMVSVSTCHTVEHHILAKRDPAPFSADRAHEGCTVNKEFHKGQTFTDSKGRTQKCSFKVNEDIQMGECFSNPRILNNVFAIFEPLDISSKSYTSEGGPYGTCTAWNCQPSQDDVQAVDDPRYTCFQWTGRHVVQSGPVATLPGNAFVPPPIVCPSNLDNCYSEEYYVSTGLDALADLVPTYSGIYFSKKVNGKPTYCNCFNCGCDRPTYAQLQSGQ

>XP_025376099.1 hypothetical protein FA10DRAFT_260834 [Acaromyces ingoldii]

MVKLALLFTITVGLYLLSTLSTVTATELQRRYRQLYGPTQLEHTQLEPTQLETTQLGNESPRRHSQFEGEETRRNPLQVGETAQPTQRTRYGPNSLDSLKTTDGLGNHEQLQGMQGYRQRHRPDTSLASTYGAPTEHGYLPTSLSDKHSLGHGSGTHKPERERAHSHGGFTKNPDYGNGGRPDPATYAFYGCFPTNSYLIQMGNHGNENDNIQMGEAFENKRSGDKPQTVASFIVNIDKFYDEGYPYGSAVGTYLPYTADDFVKVESDDEDCLSSFAIFFWDTPPPQIWENCKPFSVPPILCPENLGNCYASTYYQPGSAYLSDQGGRKSNYTGYVRSRKDKYRHQLYRPVRLRATDHCENPTYQQWSSYSDSTLPSICPNPELDKRVVYDKDGNPSDDSFPPRARIQNGVPVCTGGQKGNRGGRGGEGGGSGSGGSGGGGSGNYGGGGYGGHSHGHRPHSSSRLPHGSIRHRINKGGLPHKPMETTSSDPQVYSLEEMQT

>KAG0144215.1 hypothetical protein CROQUDRAFT_108653 [Cronartium quercuum f. sp. fusiforme G11]

MLGLSRNLSVILSLIVLILPIIHIDAKSTGHFLGKKPTAQWMKANPARKVSKDIQSAECAYNLRVPGQVFAYFQVDPTKARYNGAPYGTCFACAATPTTGQLEDNPGYNVFFWDGKTGQPGPGTGPIKNPKTGANGYENSRGVYFDGKDPTEKQT

>XP_007412896.1 uncharacterized protein MELLADRAFT_89884 [Melampsora larici-populina 98AG31]

MPGFFSLHSVLFFLITLIVSIAPLEAKPMTSMGKSHKQARNHLETRATATTLHATGHHLGKKPTQSWISANPAGKSSSNIQAAECAHNLRLGTQVFAYFEIDSTKAKNHGAPYGTCYATAAAPTASELEANAAYDVFFWNNLGGQSGVGTGPIRNPKTGVAGYEDRNGVYHDGENPDKSP

>KAH7022408.1 hypothetical protein EDB80DRAFT_806101 [Ilyonectria destructans]

MFRLSTFVSLLAIAASVSATLDPATSNTQGKYPASPNCYATKVSDAIQAAECAYNTRVSGTQTFAVFVTDHQYDGNNGAPYGTCSAYTCTAPTDEELTSDDDYWTFFWNDNGESSGVGTTCIKDPTNGVCGCENSDGTFISGSSSCV

>XP_053009558.1 Hypothetical protein NCS54_00818600 [Fusarium falciforme]

MFTLAKITSLLAIAGAASATLDPATSNTKGAYPRNPGCSPSKVSNAIQAAECAYNTRVSGQQTFAIFRVDHQYDNNNGAPYGTCEAYQCTSPTSDQMTADDDYWTFFWTGSGESSGVGTTCIKDPNDGTCGCENSDGTFIHGGTNCV

>XP_052912261.1 hypothetical protein NCS57_00861900 [Fusarium keratoplasticum]

MFPFATITSLLAIAGAASATLDPATSNTKGAYPRNPGCSPSKVSNAIQAAECAYNTRVSGQQTFAIFRVDHQYDNNNGAPYGTCEAYQCTSPTSDQMTADDDYWTFFWTGSGESSGVGTTCIKDPNDGTCGCENSDGTFIHGGTNCV

>KAJ3464210.1 hypothetical protein MRS44_008996 [Fusarium solani]

MFPLATITSLLAIAGAASATLDPATSNTKGAYPRNPGCSPSKVSNAIQAAECAYNTRVSGQQTFAIFRVDHQYDNNNGAPYGTCEAYQCTSPTSDQMTADDDYWTFFWTGSGESSGVGTTCIKDPNDGTCGCENSDGTFIHGGTNCV

>RSL60227.1 hypothetical protein CEP54_006855 [Fusarium duplospermum]

MFPLATITSLLAIAGFASATLDPATSNTKGAYPQNPGCSPSKVSNAIQAAECAYNTRVSGQQTFAIFRVDHQYDNNNGAPYGTCEAYQCTSPTSDQMTADDDYWTFFWTGSGESSGVGTTCIKDPNDGTCGCENSDGTFIHGGTNCV

>KAI8717640.1 hypothetical protein NCS52_00840500 [Fusarium sp. LHS14.1]

MFPLATITALIAIAGAASATLDPATSNTKGAYPQNPGCSPSKVSNAIQAAECAYNTRVSGQQTFAIFRVDHQYDNNNGAPYGTCEAYQCTSPTSDQMTADDDYWTFFWTGSGESSGVGTTCIKDPNDGTCGCENSDGTFIHGGTNCV

>RBQ95317.1 hypothetical protein FVER53263_09036 [Fusarium verticillioides]

MYPLTTLASVLAIAGAVTATLEPAQSNTKGKYSKSPSCSPSKTSNAIQAAECAYNTRVSGQQTFAIFKVDHQYDANNGAPYGTCEAYECDAPTSDELTADADYWTFFWNDNGESSGVGTTCIKDPNDGTCGCENFDGSFVHGGTNCK

>KNZ54183.1 hypothetical protein VP01_3016g1 [Puccinia sorghi]

MNCLSLRNLSWLAWATTILARTPRSGANWDPSTGHVRDYKPTHRWLSKNREPISSLGAQLYSILITFFLIFIASIVTSDAIQVSECALNTRLTYPNVQLFAYFEVNHAMDCYHGCPYGICHAFTTFPQPDELEPSYTDGHSFFWHNLGGNTGANPQDGEYGWEGMNGVYHDGKPDYSKMQKNHDENYPLWAQRKSALKPWPAGAAECFNNGKSEPFHPKCGRPCESERDVVLSITGEPNKDPGSVPGLYGHYHPTPASEYFPPKGWRGSCDGYSDFQSDSAYFPGNPGVNNSTSKKNRKTRKIWKKE

>KAF7547199.1 hypothetical protein G7Z17_g7894 [Cylindrodendrum hubeiense]

MFRLSTFVSLLAIAASVSATLDPATSNTQGKYPASPNCSATKVSNAIQAAECAYNTRVSGTQTFAVFVTDHQYDGNNGAPYGTCSAYTCTAPTDDELTSDDDYWTFFWNDNGESSGVGTTCIKDPTDGVCGCENSDGTFVAGSSSCV

>KAH6989381.1 small secreted protein [Ilyonectria sp. MPI-CAGE-AT-0026]

MFRLSTFVSLLAIAASVSATLDPATSNTKGKYPASPNCYATKVSNAIQAAECAYNTRVSGTQTFAVFVTDHQYDGNNGAPYGTCSAYTCTAPTDEELTSDDDYWTFFWNDNGESSGVGTTCIKDPTNGVCGCENSDGTFISGSSSCV

>KAF5682859.1 small secreted protein [Fusarium circinatum]

MYPLTTLASVLAIAGAVTATLEPAQSNTKGKYPKSPSCSPSKTSNAIQAAECAYNTRVSGQQTFAIFKVDHQYDANNGAPYGTCEAYECDAPTSDELTADADYWTFFWNDNGESSGVGTTCIKDPNDGTCGCENSDVESDWAPSANSYLDMAFFSSTSASLKPDRLNGSKTGTEMIDLCTDPFSSLLNSLVS

>RSL78008.1 hypothetical protein CEP51_008564 [Fusarium floridanum]

MFPLATITSLIAIAGFASATLDPATSNTKGAYPQNPGCSPSKVSNAIQAAECAYNTRVSGQQTFAIFRVDHQYDNNNGAPYGTCEAYQCTSPTSDQMTSDDDYWTFFWTGSGESSGVGTTCIKDPNDGTCGCENSDGTFVYGGTDCV

>RSL72022.1 hypothetical protein CEP53_001231 [Fusarium sp. AF-6]

MFPLATITSLLAIAGFASATLDPATSNTKGAYPQNPGCSPSKVSNAIQAAECAYNTRVSGQQTFAIFRVDHQYDNNNGAPYGTCEAYQCTSPTSDQMTSDDDYWTFFWTGSGESSGVGTTCIKDPNDGTCGCENSDGTFVYGGTDCV

>RSM17478.1 hypothetical protein CDV31_003727 [Fusarium ambrosium]

MFPLATITSVLAIAGFASATLDPATSNTKGAYPQNPGCSPSKVSNAIQAAECAYNTRVSGQQTFAIFRVDHQYDNNNGAPYGTCEAYQCTSPTSDQMTSDDDYWTFFWTGSGESSGVGTTCIKDPNDGTCGCENSDGTFVYGGTDCV

>RSM07097.1 hypothetical protein CEP52_005420 [Fusarium oligoseptatum]

MFPLATITSVLAIAGFASATLDPATSNTKGAYPQNPGCSPSKVSNAIQAAECAYNTRVSGQQTFAIFRVDHQYDNNNGAPYGTCEAYQCTSPTSDQMTSDDDYWTFFWTGSGESSGVGTTCIKDPNDGTCGCENSDGTFVYGGTNCV

>KAI1841522.1 hypothetical protein JX266_012274 [Neoarthrinium moseri]

MRFSAVGLVAVALPSLAMATWSPLSLFQRSVQKRTLDPATSNTKGTCPGKYNCSASKVSKAIQAAECSHNTRTSGQTFAVFVTDHQYDSSHGAPYGTCSAYSCAVPTSAEMTDSNEDCWTFFWDGSGESDGDGAGCIRSPDDGTCGCENSDGTFVPGGSDCT

>KAF5540202.1 small secreted protein [Fusarium phyllophilum]

MYPLTTLASVLAIAGAVAATLEPAQSNTKGKYPNSPSCSPSKTSNAIQAAECAYNTRVSGQQTFAIFKVDHQYDANNGAPYGTCEAYECDAPTSDELTADSDYWAFFWNDNGESSGVGTTCIKDPNDGTCGCENSDGTFVHGGTNCK

>KAF4306654.1 putative small secreted protein [Botryosphaeria dothidea]

MIKFAQIHLVPALFGALSIHTIVTCRKDHIRQQLAENKSRQCLLALSEESKPGDIIQLAECSKNTRVAKAPQYFAIFVLTTDNDKDTIAKGGPYGRCFAFACGFDQNQKVTWIKDESAVSFFWDSDTSGNLHYVDASNCIKDPNYGTCGCELSGSGTFCRGHQDCPVTNNGGLPCGKPTFRRCGYELDGYGVGLWNTDPCDKTPPANPVGTCWEKDFDRQQQPLHTWDAHICVPGSNQLQAEGSLGQAQGLVAAAAAIDATQQCPPKVNCPPAGSQSVLGGTGQLFGPQNNNQLYTRQNTGVGGFGLGGSLLQGTGGLSQSLASSGLNQLLNNCGANKGAGGGGGTGSSLYGLNSGVDANQLAGANLQSVGNVGGGSVLRNVVDNSLAGSGAVSGDITPAGFTPGQCPGSVGPPRGAGTVQNVLGGNTGQS

>KAF5546222.1 small secreted protein [Fusarium napiforme]

MYPLTTLASVLAIAGAVTATLEPAQSNTKGKYPKSPSCSPSKTSNAIQAAECAYNTRVSGQQTFAIFKVDHQYDANNGAPYGTCEAYECDAPTSDELTADSDYWTFFWNDNGESSGVGTTCIKDPNDGTCGCENSDGTFVHGGTNCK

>KAG5751922.1 hypothetical protein H9Q70_005458 [Fusarium xylarioides]

MYPLTTLASVLAIAGAVTATLEPAQSNTKGKYPNSPSCSPSKTSNAIQAAECAYNTRVSGQQTFAIFKVDHQYDANNGAPYGTCEAYECDAPTSDELTADSDYWTFFWNDNGESSGVGTTCIKDPNDGTCGCENSDGTFVHGGTNCK

>KAF5666575.1 small secreted protein [Fusarium denticulatum]

MYPLTTLASVLAIAGAVTATLEPAQSNTKGKYPKTPSCSPSKTSNAIQAAECAYNTRVSGQQTFAIFKVDHQYDANNGAPYGTCEAYECDAPTSDELTADSDYWTFFWNDNGESSGVGTTCIKDPNDGTCGCENSDGTFVHGGTNCK

>KAI8667133.1 hypothetical protein NCS56_00848600 [Fusarium sp. Ph1]

MFPLATITSLLAIAGAATATLDPATSNTKGAYPRNPGCSPSKVSNAIQAAECAYNTRVSGQQTFAIFRVDHQYDKNNGAPYGTCEAYQCTSPTSDQMTADDDYWTFFWTGSGESSGVGTTCIKDPNDGTCGCENSDGTFIHGGTNCV

>KAF5590895.1 small secreted protein [Fusarium pseudoanthophilum]

MYPLTTLASVLAAAGAVTATLEPAQSNTKGKYPKSPSCSPSKTSNAIQAAECAYNTRVSGQQTFAIFKVDHQYDANNGAPYGTCEAYECDAPTSDELTADSDYWTFFWNDNGESSGVGTTCIKDPNDGTCGCENSDGTFVHGGTNCK

>KAG7414751.1 hypothetical protein Forpe1208_v007346 [Fusarium oxysporum f. sp. rapae]

MYPLTTLASVLAIAGAVAATLEPAQSNTKGKYPESPSCSPSKTSNAIQAAECAYNTRVSGQQTFAIFKVDHQYDANNGAPYGTCEAYECDAPTSDELTADADYWTFFWNDNGESSGVGTTCIKDPNDGTCGCENSDGTFVYGGTNCK

>KAF5596201.1 small secreted protein [Fusarium pseudocircinatum]

MYPLTTLASVLAVVGAVTATLEPAQSNTEGKYPKSPSCSPSKTSNAIQAAECAYNTRVSGQQIFAIFKVDHQYDANNGAPYGTCEAYECDAPTSDELTTDADYWTFFWNDNGESSGVGTTCIKDPNDGTCGCENSDGTFVYGGTNCK

>XP_041687096.1 uncharacterized protein FMAN_07893 [Fusarium mangiferae]

MYPLTTLASVLAIAGAVTATLEPAQSNTEGKYPKSPSCSPSKTSNAIQAAECAYNTRVSGQQTFAIFKVDHQYDANNGAPYGTCEAYECDAPTSDELTADADYWTFFWNDNGESSGVGTTCIKDPNDGTCGCENSDGTFVHGGTNCK

>KAI7782618.1 hypothetical protein LA080_013106 [Diaporthe eres]

MHFTSFFSLLAAPAMVLATLDPATSNTKGKYPASPACSATKVSKAIQAAECSHNTRVSGQQTFAVFETDHQYDSSHGAPYGTCSAYTCEAPTSSEMTADEDYWTFFWTGEGESSGEGAGCIKSPVDGTCGCENSDGTFVAGSDSCT

>KAF5545342.1 small secreted protein [Fusarium mexicanum]

MYPLTTLASVLAITGAVTATLDPAQSNTEGKYPKSPSCSPSKTSNAIQAAECAYNTRVSGQQTFAIFKVDHQYDANNGAPYGTCEAYECDAPTSDELTADADYWTFFWNDNGESSGVGTTCIKDPNDGTCGCENSDGTFVYGGTDCK

>KAG4280898.1 hypothetical protein FPRO04_05612 [Fusarium proliferatum]

MYPLTTLASVIAIAGVVTATLEPAQSNTKGKYPKSPSCSPSKTSNAIQAAECAYNTRVSGQQTFAIFKVDHQYDANNGAPYGTCEAYECDAPTSDELTADADYWTFFWNDNGESSGVGTTCIKDPNDGTCGCENSDGTFVHGGTNCK

>XP_036544457.1 small secreted protein [Fusarium subglutinans]

MYPLTTLASVLAIAGAVTATLEPAQSNTKGKYPKSPSCSPSKTSNAIQAAECAYNTRVSGQQTFAIFKVDHQYDANNGAPYGTCEAYECDAPTSDELTADADYWTFFWNDNGESSGVGTTCIKDPNDGTCGCENSDGTFVHGGTNCT

>KAF5236321.1 hypothetical protein FANTH_11273 [Fusarium anthophilum]

MYPLTTLASVLAIAGAVTATLDPAQSNTEGKYPKSPSCSPSKTSNAIQAAECAYNTRVSGQQTFAIFKVDHQYDANNGAPYGTCEAYECDAPTSDELTADADYWTFFWNDNGESSGVGTTCIKDPNDGTCGCENSDGTFVHGGTNCK

>OKP11508.1 hypothetical protein PENSUB_2994 [Penicillium subrubescens]

MRFTSLLALVATPVLLVTATLYPATSNTKGYKPKSLNCSASKVSTAIQAAECSHNTRVSGTQTFAIFQTDHQYDSSNGAPYGTCSAYTCTAPTFSELEADDDYWAFYWGDEDQGQSSGVGTGCIKDPSSGECGCEDSDGVFVVGSDSCT

>XP_031059164.1 uncharacterized protein FOIG_10761 [Fusarium odoratissimum NRRL 54006]

MYPLTTLASVLAVAGAVTATLEPAQSNTKGKYPKSPSCSPSKTSNAIQAAECAYNTRVSGQQTFAIFKVDHQYDANNGAPYGTCEAYECDAPTSDELTADADYWTFFWNDNGESSGVGTTCIKDPNDGTCGCENSDGTFVYGGTNCK

>KAI0477078.1 small secreted protein [Xylariaceae sp. FL0804]

MVAIRSLTGQVAVLALSVQALASPASGMHKRTLDTCSSNTKGKCPGTLNCSAAKVSTAIQAAECSHNTRTSGAQTFAVFETDHQYDGSHGAPYGTCSAYTCAAPTAGDMTDADDDCWTFFWAGQGESNGTGTGCIRSPDDGTCGCEDSDGTFVYGSDSCT

>XP_043017518.1 uncharacterized protein INS49_004968 [Diaporthe citri]

MHFTSFFSLVAAPAMVLATLDPATSNTKGKCPTSPACSATKVSKAIQAAECSHNTRVSGQQTFAVFETDHKYDSSHGAPYGTCSAYTCEAPTSADMKADEDCWTFFWTGEGESSGEGAGCIKSPDDGTCGCENSDGTFVAGSDSCT

>KAF7538191.1 hypothetical protein G7054_g3147 [Neopestalotiopsis clavispora]

MKSTVVLGLVAAVLPSITAAWSPLDLFQRSAEVQVQKRTLDPATSNTEGKCPSTYNCSASKTSKSIQAAECAYNTRTSSQTFAVFKTDHQYDSVEGAPYGTCSAYSCTVPTSSEMTDSDSDCWTFFWDDSGESSGVGTTCIKDPSSGECGCENSDGTFIVGSDSCT

>KAF3023128.1 hypothetical protein E8E14_013721 [Neopestalotiopsis sp. 37M]

MKSTVILGLVAAVLPSITAAWSPLDLFQRSAEVQVQKRTLDPATSNTEGKCPSTYNCSASKTSKSIQAAECAYNTRTSSQTFAVFKTDHQYDSVEGAPYGTCSAYSCTVPTSSEMTDSDSDCWTFFWDDSGESSGVGTTCIKDPSSGECGCENSDGTFIVGSDSCT

>KAA8895541.1 small secreted protein [Sphaerosporella brunnea]

MFTLRKLLAIAALALTASATLDPATSNTKGKCPCTYNCSAAKVSHAIQAAECSHNTRVHGTQTFAVFVTDHKYDHSHGAPYGNCSAYTCTAPTSAEMTVVDDDCWTFFWGNQGEETGYGTGCIKDPHDGTCGCENSNGVFVKGSDSCV

>KAF2672361.1 hypothetical protein BT63DRAFT_438172 [Microthyrium microscopicum]

MLNFYCFIFLLAALAVNGTLAPCQTNTKGKYPKNPGCSPSKVSKAIQAAECSYNTRVSGKQTFAIFHVDHKYDKSHGAPYGTCEAYTCAAPRDSDLVDDKDSWTFFWSQNGKQAGVGTGCIRDPKDGTSTFTTLRSCCCSPLVPLLQFTVVPLMLPVLLSLSLLPAVTYQQSSYVSFQNPYYIGGGWEGGYTHNEVWLLGSTQNIQWNTSLTTYNITLWQQNLTAPRATPAETPVLMITDFEPGVNHSMNWIVDHYDLPLEDSPVFKLWLNFSTTNGYTSPYFNITRNASEVTQSTISSSLVSTSSSTSSTTMMPTSKPVNAGLDVSTRLGLGIGLGIGIPFVMLLCIVTGIVFWMLRKKSMKNMGTEEAWLDNQPGNPGLSAFKYVDPIYEAPNDHESYQLGQPSSPAELADQRSEH

>KAH7163591.1 small secreted protein [Dactylonectria estremocensis]

MFRLSTVVSLLAIVASVSATLDPATSNTKGKYPANPNCSATKVSTAIQAAECAYNTRVSGTQTFAVFVTDHQYDGNNGAPYGTCSAYTCTAPTDYELTSNDDYWTFFWNDNGEDSGVGTTCIKSPDDGTCGCENSDGTFVYGGTSCV

>XP_052992429.1 uncharacterized protein J7T55_010582 [Diaporthe amygdali]

MHFTSFFSLLAAPAMVLATLDPATSNTKGKKPSSTNCSATKVSKAIQAAECSHNTRVSGTQTFAVFVTDHQYDSSHGAPYGTCSAYTCESPTDSEMEADEDSWTFFWSGEGESSGEGAGCIKSPDDGTCGCENSDGTFVYGSDSCT

>KAH7061243.1 hypothetical protein B0J12DRAFT_565417 [Macrophomina phaseolina]

MQFTTLATLLAAATAVTATLDPCTSNSKGKVPKNPPCSPEESSGDIQAAECSHNTRVSGTQTFAVWTRTKTNSNGISYGTCEAYTCTAPTSDELGDNPDGWTFFWDSNGEESGFGTTCIQDPNTGECGCENSNGDFIVGSDSCS

>KAH6887612.1 hypothetical protein B0T10DRAFT_607528 [Thelonectria olida]

MARLSILASILAVAASVSATLDPADSNTKGKYPANPACSPAKTSTAIQAAECAYNTRVSGTQTFAVFITDHQYDGNNGAPYGTCMAYTCTAPTDSELTVNSDYWTFFWNSNGESSGVGTGCIKDPTDGTCGCENSDGTFVKGSSSCV

>EOD52258.1 putative small secreted protein [Neofusicoccum parvum UCRNP2]

MFKLTSLISVLAVAATVSATLDPATSNTKGKYPASPNCSAAKTSTAIQAAECSHNTRVSGTQTFAIFKTDHQYDSSHGAPYGTCEAYTCTAPTDSELTDGEDYWTFFWNDNGEDSGVGTGCIKSPDDGTCGCENSDGEFIYGGTDCS

>KAE8354272.1 hypothetical protein BDV28DRAFT_156365 [Aspergillus coremiiformis]

MVSATSLVAVIFGASLAAATLDPALSNTKGKCPGTPKCSAAKTSNAIQASECSHNTRTSQKQTFAVFVTDHKYDSSHGAPYGTCTAYTCTPPTGAEMTDSNPDCWTFFWSGQGESSGEGAGCIRSPDDGTCGCENSTGEFVPGSNSCK

>KAF7593598.1 hypothetical protein BBP40_011219 [Aspergillus hancockii]

MVSASSLFALVLSASLAAATLDPASSNTKGKCPGTVKCSPAKTSNAIQAAECSHNTRTSGTQTFAVFTTDHQYDSSHGAPYGTCKAYTCTAPTSSEMTDSDDDCWTFFWNNNGESSGVGTGCIKSPDDGTCGCENSDGTFVYGGSNCS

>PYI02525.1 small secreted protein [Aspergillus sclerotiicarbonarius CBS 121057]

MLAKSLLTVVLGAVLATATVDPATTNTKGKCPGTYNCSAAKTSSAIQAAECSHNTRTSGTQTFAVFVTDHKYDSSHGAPYGTCSAYTCTAPTDSEMTDSDDDCWTFFWNDNGESSGSGTGCIRSPEDGTCGCEDSDGTFVYGGSNCS

>KAF9637446.1 putative small secreted protein [Lasiodiplodia theobromae]

MFKLTSILLVLAVAATVSATLDPATSNSKGKYPASPGCSPEKTSTVIQAAECAYNTRVSGTQTFAVFKQDHQYDENNGAPYGTCEAYTCTAPTSDELTEDDDYWTFFWGDNGDSSGVGTTCIKSPDDGTCGCENSDGTFVYGGTDCS

>XP_038802689.1 uncharacterized protein EKO05_000147 [Ascochyta rabiei]

MFAKSIVTVLTLAVAATATLDPATSNTKGKYPSSPSCSVTKTSKAIQAAECSHNTRVSGKQTFAVFTQDHQYDGNHGYPYGTCEAYTCAPGTAMKSSKDKWTFFWSNAGESSGDGAGCIKSPKDGTCGCENSDGTFVYGASNCK

>KAF1777024.1 hypothetical protein GQ600_3674 [Phytophthora cactorum]

MEFIYPSEAKQKHELTLQFAIYSISKTNQYAPHFCPRSLAIPALVSATLDPCSSNSKGKCPSTYNCSATKVSTAIQAAECSHNTRTSKTQTFAVFVTDHQYDGNNGYPYGTCSAYTCDSPTSDEIEDNDDCWTFFWSGNGTDSGVGTGCIKDPTTGDCGCESSDGTFVADSSSCVCCRRDLVAELVENPSCTKVIALTRREIPETQWGNAFPSMDVATAQNKLEIIPVDFEELNRDWKKIPVDVDAASRVWARPVKMAGSAEAFRKG

>KAG6944941.1 hypothetical protein JG688_00016823 [Phytophthora aleatoria]

MRLIFVLALLAIPALVSATLDPCSSNSKGKCPSTYNCSATKVSTAIQAAECSHNTRTSKTQTFAVFVTDHQYDGNNGYPYGTCSAYTCDPPTSDEIEDNDDCWTFFWSGNGTDSGVGTGCIKDPTTGDCGCESSDGTFVADSSSCV

>XP_009515305.1 hypothetical protein PHYSODRAFT_321736 [Phytophthora sojae]

MRFSFALVLLVAPALVSATLDPATSNTKGKCPSVYNCSATKVSTAIQAAECSHNTRTSETQTFAVFVTDHQYDGNNGYPYGTCSAYTCESPTADVMTANDDCWTFFWSGNGTDSGVGTGCIKDPTTGNCGCENSDGQFIADSSSCV
