# Supplementary material for: Horizontally Transferred Salivary Protein Promotes Insect Feeding by Suppressing Ferredoxin-Mediated Plant Defenses
Source: Mol Biol Evol. 2023 Oct 7;40(10):msad221. doi: 10.1093/molbev/msad221 (PMC10583550; doi:10.1093/molbev/msad221)
Supplement: msad221_Supplementary_Data [file msad221_supplementary_data.zip › Figure and Table 8.0.docx]

Supplementary Materials for

**Horizontally transferred salivary protein promotes insect feeding by suppressing ferredoxin-mediated plant defenses**

Yi-Zhe Wang *et al.*

*Corresponding author. Jun-Min Li, lijunmin@nbu.edu.cn; Chuan-Xi Zhang, chxzhang@zju.edu.cn; Hai-Jian Huang, huanghaijian@nbu.edu.cn

**This PDF file includes:**

Figs. S1 to S10

Tables S1 to S5

**Other Supplementary Materials for this manuscript include the following:**

Data S1 to S2


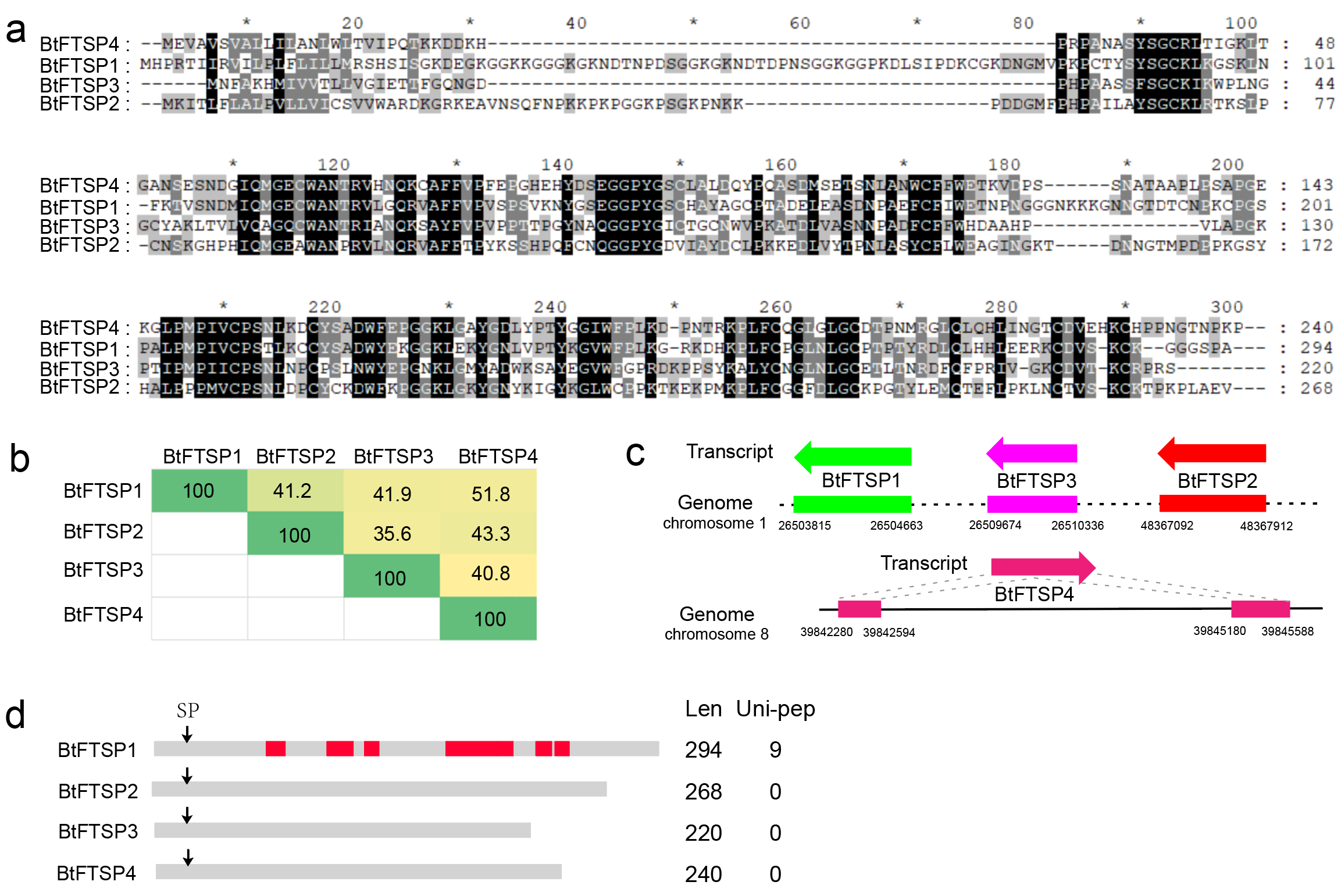


**Fig. S1 Identification of four** **BtFTSPs in** ***Bemisia tabaci*. a** Amino acid alignments of BtFTSPs. The sequences of the four BtFTSPs were aligned using ClustalX software. Black shading indicates the conserved regions of BtFTSPs. **b** Comparison of amino acid identity among BtFTSPs. The pairwise distances between the amino acid sequences of BtFTSPs were calculated using MegAlign. **c** Distribution of BtFTSPs in *B. tabaci* genome. The BtFTSP1, BtFTSP2, and BtFTSP3 were located in chromosome 1, while BtFTSP4 was located in chromosome 8. One intron was detected in BtFTSP4. Analysis was performed based on chromosome-level genome of *B. tabaci* in NCBI (accession, GCA_918797505.1). **d** Distribution of unique peptides identified by LC-MS/MS. Unique peptides mapped to BtFTSPs are labeled in red. The number of unique peptides in each protein are displayed on the right. The amino acid length of each protein is also shown. Arrow indicates the signal peptide (SP) cleavage site.


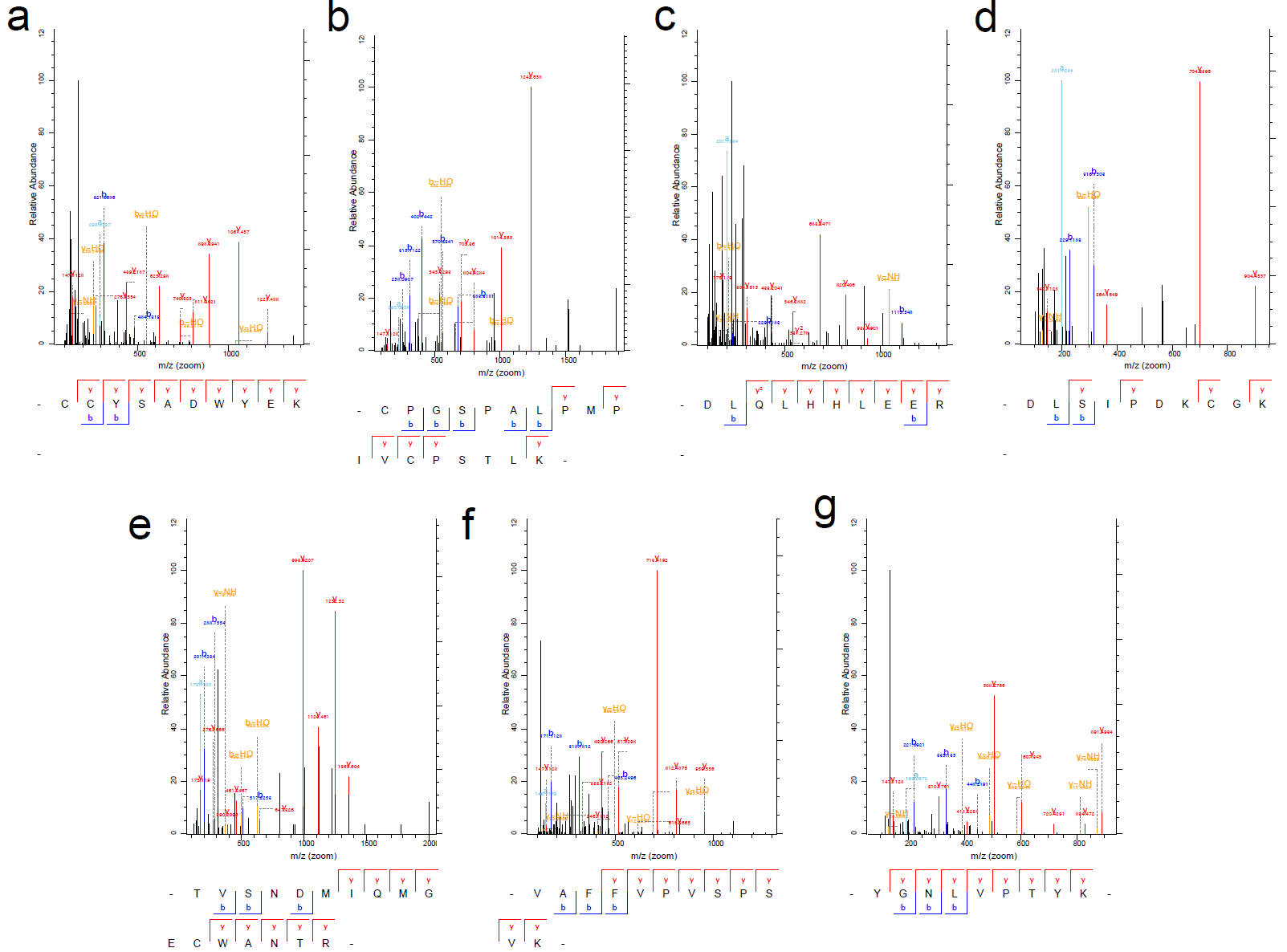


**Fig. S2 Mass spectrums of identified unique peptides**. The spectrums of seven unique peptides were displayed in **a**-**g**.


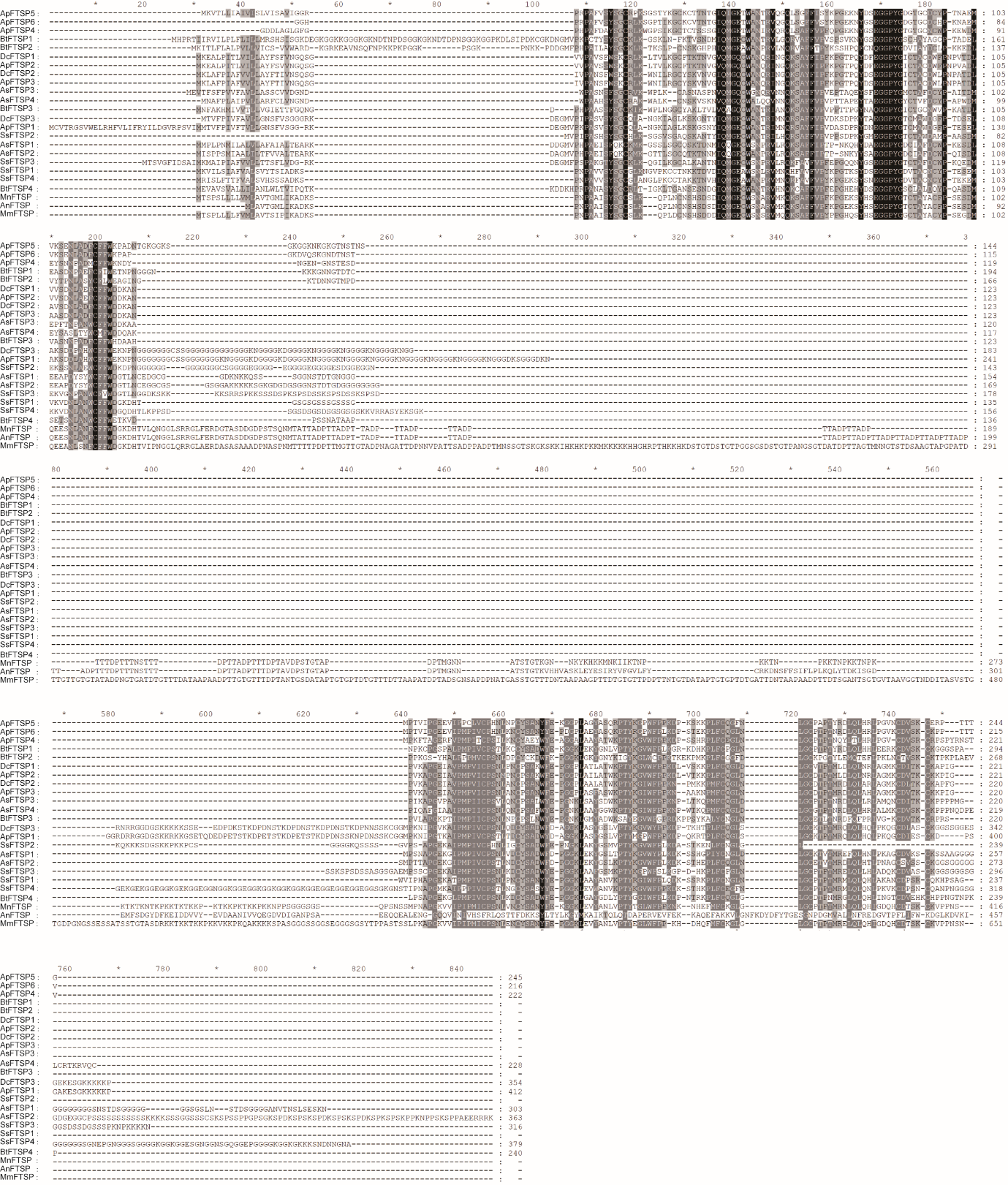


**Fig. S3 Amino acid aliment of FTSPs from insects and three fungi.** The sequences of 24 FTSPs from insects (*Bemisia tabaci*, *Trialeurodes vaporariorum*, *Dialeurodes* *citri*, *Aleurocanthus* *spiniferus*, *Aleuroclava* *psidii*, and *Singhiella* *simplex*) and 3 FTSPs from fungal (*Meira* *miltonrushii*, *Meira* *nashicola*, and *Adiantum* *nelumboides*-associated fungus) were aligned using ClustalX software. Black shading indicates the conserved regions of FTSPs.


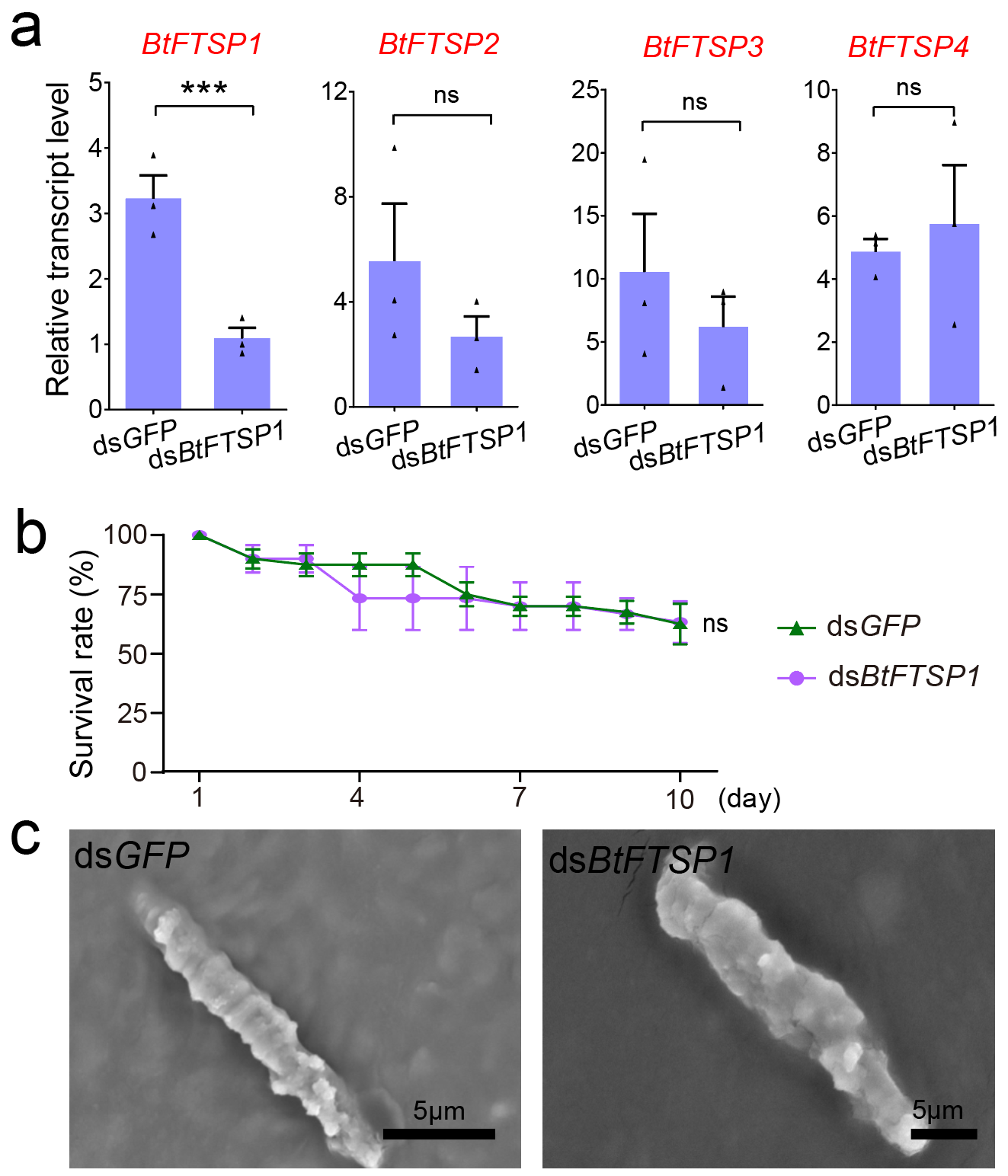


**Fig. S4 Effects of dsRNA treatment on** ***Bemisia tabaci*. a** Efficiency and specify of RNA interference. Newly emerged *B.* *tabaci* adults were treated with ds*GFP* and ds*BtFTSP1*, and the relative transcript level of *BtFTSP1*, *BtFTSP2*, *BtFTSP3*, and *BtFTSP4* were determined by qRT-PCR. **b** Effects of dsRNA treatment on insect survivorship. Mortality was recorded for ten consecutive days, and differences in survivorship between the two treatments were tested by log-rank test. ns, not significant. **c** Effects of dsRNA treatment on salivary sheath formation. The dsRNA-treated *B.* *tabaci* were fed on artificial diets. The salivary sheaths left on parafilm were inspected by scanning electron microscopy


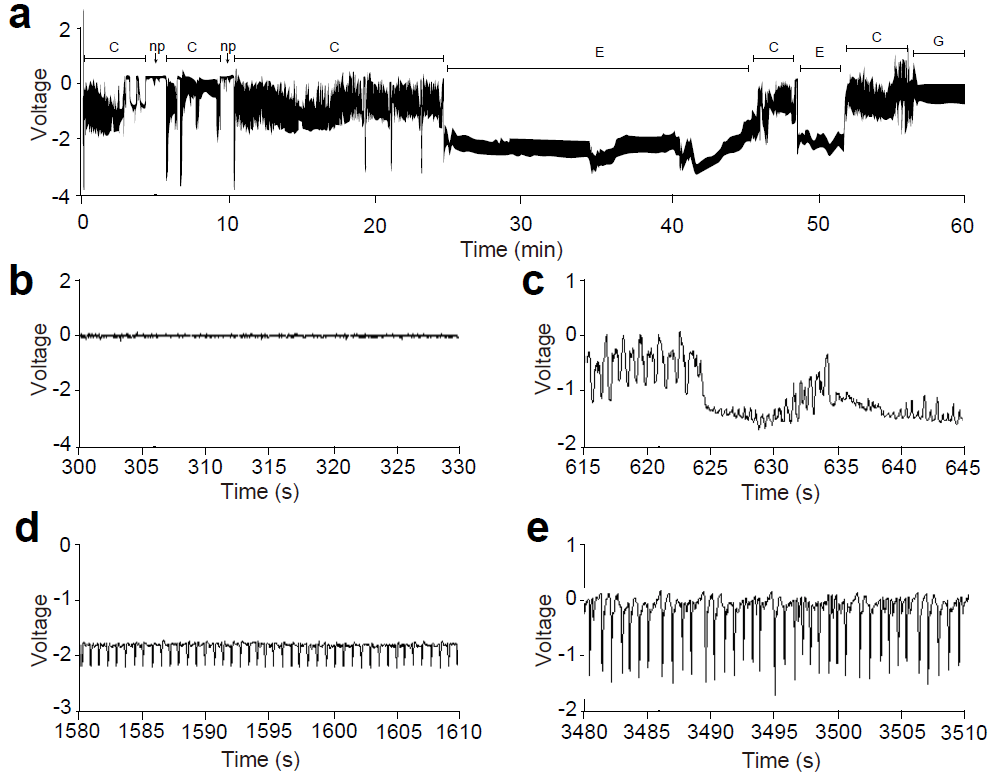


**Fig. S5 Typical waveforms detected by EPG recording. a** Overall EPG waveforms over 1 h

for *Bemisia tabaci* feeding on tobacco plants. **b-e** Typical waveforms for nonpenetration (**b**), pathway duration (**c**), phloem sap ingestion (**d**), and xylem sap ingestion (**e**).


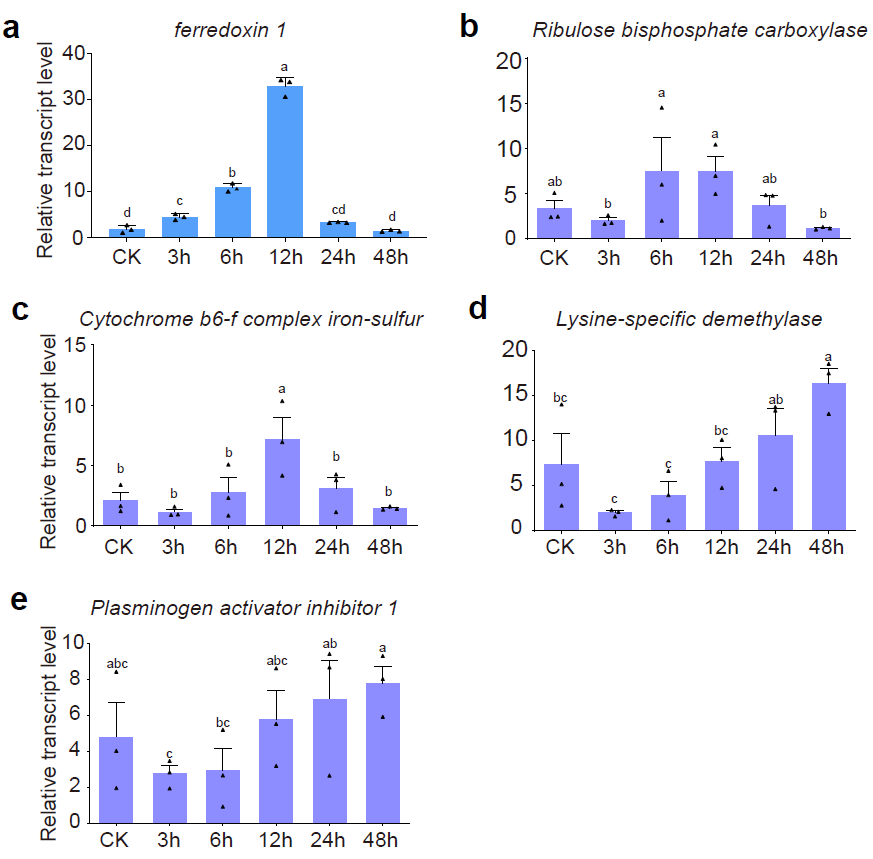


**Fig. S6 Expression patterns of BtFTSP1-interacting genes in response to *Bemisia tabaci* infestation.** Relative transcript level of *ferredoxin 1* (**a**), *ribulose bisphosphate carboxylase* (**b**), *cytochrome b6-f complex iron-sulfur* (**c**), l*ysine-specific demethylase* (**d**), and *plasminogen activator inhibitor* (**e**) in response to *B. tabaci* infestation were determined by qRT-PCR. Data are presented as mean values ± SEM (n = 3 independent biological replicates). Different lowercase letters indicate statistically significant differences at *P* < 0.05 level according to one-way ANOVA test followed by Tukey’s multiple comparisons test.


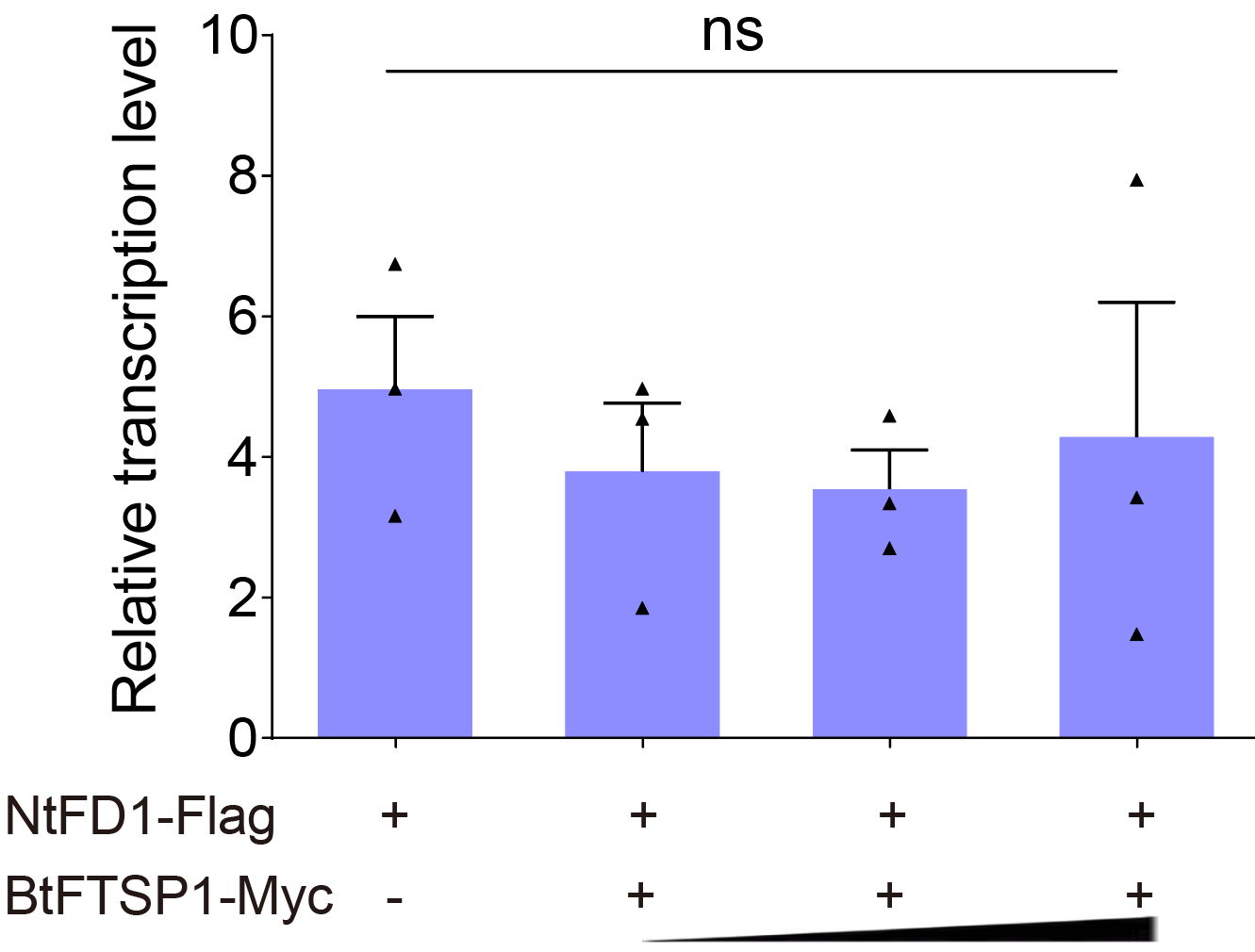


**Fig. S7 Effects of BtFTSP1 on NtFD1 transcripts.** NtFD1-Flag was co-expressed with different concentration of BtFTSP1-Myc in *Nicotiana benthamiana*. The transcript level of NtFD1 was quantified by qRT-PCR. *P*-value were determined by one-way ANOVA test followed by Tukey’s multiple comparisons test. ns, no significant difference in all comparison groups.


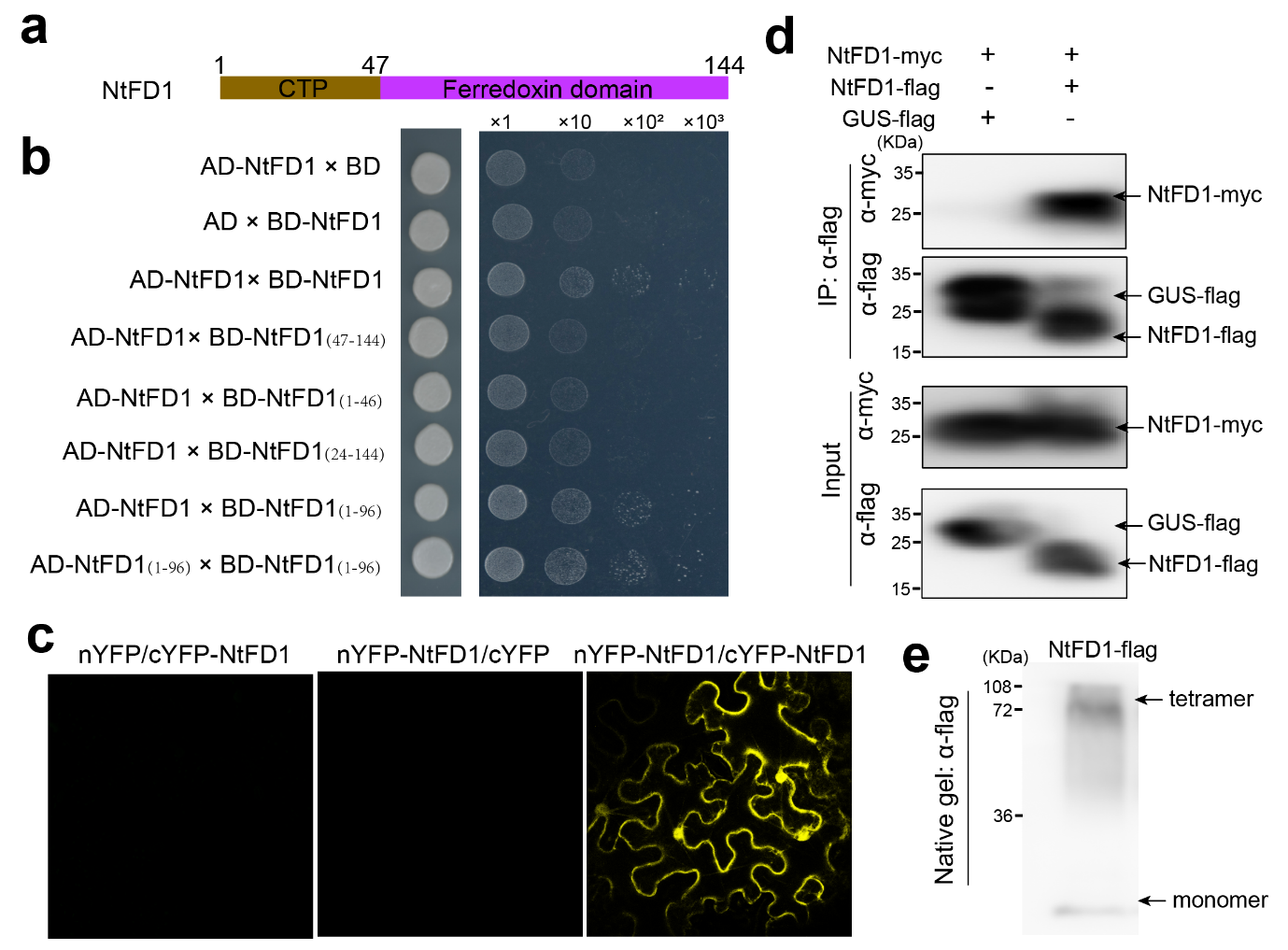


**Fig. S8 NtFD1 interacts with one another to form polymer. a** Domain organization of NtFD1. NtFD1 contains a chloroplastic transit peptide (cTP) at the N-terminal and a ferredoxin domain at the C-terminal. **b** Yeast two-hybrid assays showing that NtFD1 interacts with one another. Mutation assays further confirmed that the NtFD1^Δ1-96^ is critical for NtFD1-NtFD1 interaction. **c**, **d** Co-immunoprecipitation (Co-IP, **c**) and bimolecular fluorescence complementation (BiFC, **d**) assays confirmed the NtFD1-NtFD1 interaction. In BiFC assay, the nYFP-tag and cYFP-tag was expressed the N-terminal of NtFD1. **e** Native PAGE assay showing that NtFD1 mainly formed tetramer. The NtFD1-Flag was expressed in *N. benthamiana* leaves. The protein sample was directly loaded onto native PAGE gel, and underwent western blotting analysis. The native electrophoresis protein marker GAPDH was used to indicate the molecular weight.


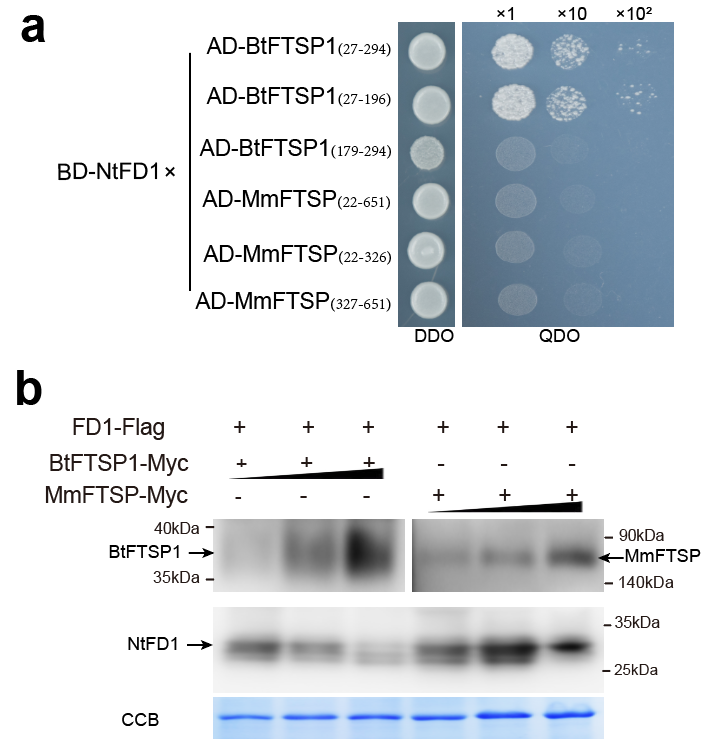


**Fig. S9** **MmFTSP-like fails to interact with NtFD1. a** Yeast two-hybrid assays showing that MmFTSP-like failed to interact with NtFD1. **b** MmFTSP-like cannot destabilize NtFD1. The BtFTSP1 was used as a positive control. The protein level of NtFD1-Flag, MmFTSP-like-Myc and BtFTSP1-Myc were quantified by western-blotting. Coomassie brilliant blue (CBB) staining was conducted to visualize the amount of sample loading.


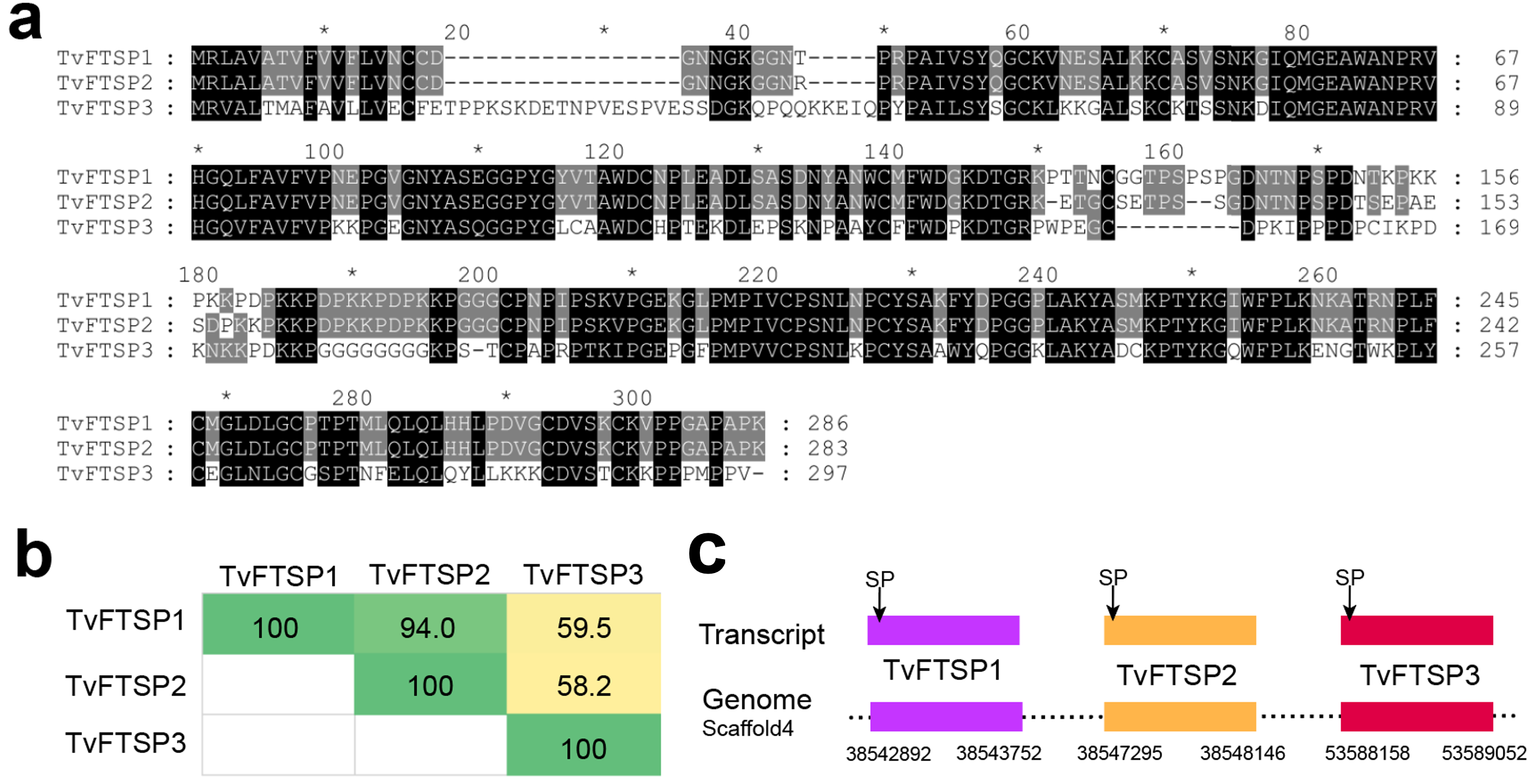


**Fig. S10 Identification of three TvFTSPs in** ***Trialeurodes vaporariorum*. a** Amino acid alignments of TvFTSPs. Three TvFTSP sequences were aligned using ClustalX software. Black shades indicate the conserved regions of TvFTSPs. **b** Comparison of amino acid identity among TvFTSPs. The pairwise distances between the amino acid sequences of TvFTSPs were calculated using MegAlign. **c** Distribution of TvFTSPs in *T. vaporariorum* genome. Three TvFTSP were tandemly arrayed in the Scaffold 4 in *T. vaporariorum* genome. Arrow indicates the signal peptide (SP) cleavage site.

**Supplementary Table 1 Chromosomal location of BtFTSPs and their adjacent genes**

|  | **Gene ID** | **Gene loci**^1^ | **Description** | **Best matched species in NCBI** ^2^ | **E-value** | **Classification** |
| --- | --- | --- | --- | --- | --- | --- |
| Region1 | LOCUS781 | Chr1: 26126284-26133338 | protein gustavus | *Schistocerca gregaria* | 0.0 | Insect |
|  | LOCUS782 | Chr1: 26183596-26196465 | *B. tabaci*-specific gene | *-* | - | - |
|  | LOCUS783 | Chr1: 26312480-26441491 | histone-lysine N-methyltransferase | *Cryptotermes secundus* | 0.0 | Insect |
|  | LOCUS784 | Chr1: 26503059- 26506798 | BtFTSP1 | *Violaceomyces palustris* | 1e-30 | Fungus |
|  | LOCUS785 | Chr1: 26509148-26512003 | BtFTSP3 | *Adiantum nelumboides* | 1e-22 | Fungus |
|  | LOCUS786 | Chr1: 26580490-26581738 | *B. tabaci*-specific gene | *-* | - | - |
|  | LOCUS787 | Chr1: 26697211-26830937 | MDS1 and EVI1 complex locus protein | *Diaphorina citri* | 2e-166 | Insect |
|  | LOCUS788 | Chr1: 26958265-27139925 | calcium ion binding | *Macrosteles quadrilineatus* | 0.0 | Insect |
| Region2 | LOCUS1228 | Chr1: 48273340-48284854 | uncharacterized protein | *Cinara cedri* | 1e-143 | Insect |
|  | LOCUS1229 | Chr1: 48303733-48314432 | uncharacterized protein | *Diploptera punctata* | 9e-07 | Insect |
|  | LOCUS1230 | Chr1: 48326736-48344525 | uncharacterized protein | *Cimex lectularius* | 7e-05 | Insect |
|  | LOCUS1231 | Chr1: 48363975-48367730 | BtFTSP2 | *Adiantum nelumboides* | 3e-20 | Fungus |
|  | LOCUS1232 | Chr1: 48388913-48403253 | *B. tabaci*-specific gene | *-* | - | - |
|  | LOCUS1233 | Chr1: 48425844-48515073 | chorion peroxidase | *Nilaparvata lugens* | 0.0 | Insect |
|  | LOCUS1234 | Chr1: 48541676-48561025 | zinc metalloproteinase | *Timema cristinae* | 2e-96 | Insect |
| Region3 | LOCUS13193 | Chr8: 39604051-39635121 | carbonic anhydrase | *Macrosteles quadrilineatus* | 4e-102 | Insect |
|  | LOCUS13194 | Chr8: 39657878-39774828 | epsin-2-like | *Nilaparvata lugens* | 2e-10 | Insect |
|  | LOCUS13195 | Chr8: 39785302-39828164 | uncharacterized protein | *Aphis gossypii* | 8e-75 | Insect |
|  | LOCUS13196 | Chr8: 39835790-39845737 | BtFTSP4 | *Meira miltonrushii* | 1e-29 | Fungus |
|  | LOCUS13197 | Chr8: 39882990-39885961 | folate receptor gamma-like | *Ischnura elegans* | 6e-84 | Insect |
|  | LOCUS13198 | Chr8: 39939781-40145259 | peroxidase | *Daktulosphaira vitifoliae* | 0.0 | Insect |
|  | LOCUS13199 | Chr8: 40158912-40165884 | uncharacterized protein | *Vigna radiata* | 3e-08 | Plant |

^1^ Gene loci was displayed based on the chromosome-level *Bemisia tabaci* genome (GCA_918797505.1). Three genes adjacent to BtFTSPs were displayed.

^2^ The best matched species was identified using BLASTp search against NCBI nr database. The top one matched species, excluding *B. tabaci*, was displayed for each gene. Genes that specifically identified in *B. tabaci* were labeled as “-”

**Supplementary Table 2 Identification of FTSP homologues in Hemiptera species**

| **Family** | **Species** | **Accession** | **Total Base** | **Number of** **FTSP homologues** | **Note** |
| --- | --- | --- | --- | --- | --- |
| Aleyrodidae | *Trialeurodes vaporariorum* | GCA_011764245 | 814.7 MB | 3 | Assembled genome |
|  | *Bemisia tabaci* | GCA_003994315 | 657.5 MB | 4 | Assembled genome |
|  | *Singhiella simplex* | ERR3781281 | 22.7Gb* | 4 | Whole genome-seq |
|  | *Aleurocanthus spiniferus* | SRR17330024 | 9.2Gb* | 4 | RNA-seq, head and body |
|  | *Aleyrodes proletella* | SRX14998513-SRX14998536 | 1.6-4.4Gb* | 0 | RNA-Seq, whole body |
|  | *Dialeurodes citri* | SRR2980521 | 5.0Gb* | 3 | RNA-Seq, whole body |
|  | *Aleuroclava psidii* | SRR16114381 | 29.5 Gb* | 6 | Whole genome-seq |
| Delphacidae | *Nilaparvata lugens* | GCF_014356525 | 1,088 MB | 0 | Assembled genome |
|  | *Laodelphax striatellus* | GCA_017141395 | 510.2 MB | 0 | Assembled genome |
|  | *Sogatella furcifera* | GCA_017141385 | 563.8 MB | 0 | Assembled genome |
| Aphididae | *Acyrthosiphon pisum* | GCA_005508785 | 533.6 MB | 0 | Assembled genome |
|  | *Myzus persicae* | GCA_001856785 | 347.3 MB | 0 | Assembled genome |
|  | *Sitobion miscanthi* | GCA_008086715 | 397.9 MB | 0 | Assembled genome |
| Aphalaridae | *Pachypsylla venusta* | GCA_012654025 | 482.0 MB | 0 | Assembled genome |
|  | *Diaci psyllid* | GCA_000475195 | 485.7 MB | 0 | Assembled genome |
| Alydidae | *Riptortus pedestris* | GCA_019009955 | 1,080 MB | 0 | Assembled genome |
| Lygaeidae | *Oncopeltus fasciatus* | GCA_000696205 | 1,100 MB | 0 | Assembled genome |
| Pentatomidae | *Halyomorpha halys* | GCA_000696795 | 998.2 MB | 0 | Assembled genome |
| Cimicidae | *Cimex lectularius* | GCA_000648675 | 510.8 MB | 0 | Assembled genome |
| Reduviidae | *Rhodnius prolixus* | GCA_000181055 | 706.8 MB | 0 | Assembled genome |
| Gerridae | *Gerris buenoi* | GCA_001010745 | 994.4 MB | 0 | Assembled genome |

^*^ The total base of raw data retrieved from SRA database.

**Supplementary Table 3 Sequences used for constructing phylogenetic tree**

| **Gene** | **Classification** | **Phylum** | **Species** | **ID** |
| --- | --- | --- | --- | --- |
| BtFTSP1 | insect | Protostomia | *Bemisia tabaci* | QHB15613 |
| BtFTSP2 | insect | Protostomia | *Bemisia tabaci* | XP_018909720 |
| BtFTSP3 | insect | Protostomia | *Bemisia tabaci* | CAH0381103 |
| BtFTSP4 | insect | Protostomia | *Bemisia tabaci* | XP_018907192 |
| SsFTSP1 | insect | Protostomia | *Singhiella simplex* | NODE_110072 |
| SsFTSP2 | insect | Protostomia | *Singhiella simplex* | NODE_4789 |
| SsFTSP3 | insect | Protostomia | *Singhiella simplex* | NODE_30271 |
| SsFTSP4 | insect | Protostomia | *Singhiella simplex* | NODE_19893 |
| DcFTSP1 | insect | Protostomia | *Dialeurodes citri* | NODE_29096 |
| DcFTSP2 | insect | Protostomia | *Dialeurodes citri* | NODE_20410 |
| DcFTSP3 | insect | Protostomia | *Dialeurodes citri* | NODE_6494 |
| AsFTSP1 | insect | Protostomia | *Aleurocanthus spiniferus* | NODE_28436 |
| AsFTSP2 | insect | Protostomia | *Aleurocanthus spiniferus* | NODE_18108 |
| AsFTSP3 | insect | Protostomia | *Aleurocanthus spiniferus* | NODE_89821 |
| AsFTSP4 | insect | Protostomia | *Aleurocanthus spiniferus* | NODE_89822 |
| TvFTSP1 | insect | Protostomia | *Trialeurodes vaporariorum* | VMOF01000320.1 38542892-38543752 |
| TvFTSP2 | insect | Protostomia | *Trialeurodes vaporariorum* | VMOF01000320.1 38547295-38548146 |
| TvFTSP3 | insect | Protostomia | *Trialeurodes vaporariorum* | VMOF01000320.1 53589052-53588000 |
| ApFTSP1 | insect | Protostomia | *Aleuroclava psidii* | NODE_063420 |
| ApFTSP2 | insect | Protostomia | *Aleuroclava psidii* | NODE_032806 |
| ApFTSP3 | insect | Protostomia | *Aleuroclava psidii* | NODE_126363 |
| ApFTSP4 | insect | Protostomia | *Aleuroclava psidii* | NODE_016320 |
| ApFTSP5 | insect | Protostomia | *Aleuroclava psidii* | NODE_268628 |
| ApFTSP6 | insect | Protostomia | *Aleuroclava psidii* | NODE_211042 |
| PcacFTSP-like | oomycete | Oomycota | *Phytophthora cactorum* | KAF1777024.1 |
| PaleFTSP-like | oomycete | Oomycota | *Phytophthora aleatoria* | KAG6944941.1 |
| PsojFTSP-like | oomycete | Oomycota | *Phytophthora sojae* | XP_009515305.1 |
| MnasFTSP-like | fungus | Basidiomycota | *Meira nashicola JCM 18503* | BCJU01000001.1 |
| MmilFTSP-like | fungus | Basidiomycota | *Meira miltonrushii* | XP_025357528.1 |
| ErhoFTSP-like 1 | fungus | Basidiomycota | *Exobasidium rhododendri* | UZJ51548.1 |
| ErhoFTSP-like 2 | fungus | Basidiomycota | *Exobasidium rhododendri* | UZJ51547.1 |
| AnelFTSP-like | fungus | Basidiomycota | *Adiantum nelumboides-associated fungus* | MCO5562931.1 |
| EcylFTSP-like | fungus | Basidiomycota | *Exobasidium cylindrosporum* | CP096878.1 |
| AingFTSP-like | fungus | Basidiomycota | *Acaromyces ingoldii* | XP_025376099.1 |
| CbomFTSP-like | fungus | Basidiomycota | *Ceraceosorus bombacis* | CEH17835.1 |
| TpalFTSP-like | fungus | Basidiomycota | *Tilletiopsis pallescens* | BCHO01000002.1 |
| GBC081FTSP-like | fungus | Basidiomycota | *Golubevia sp. BC0812* | JAAVVH010000005.1 |
| GBC0850FTSP-like | fungus | Basidiomycota | *Golubevia sp. BC0850* | JAAVVG010000009.1 |
| GBC0902FTSP-like | fungus | Basidiomycota | *Golubevia sp. BC0902* | JAAVVI010000001.1 |
| TthlFTSP-like | fungus | Basidiomycota | *Thecaphora thlaspeos* | UWYS01000030.1 |
| PfloFTSP-like | fungus | Basidiomycota | *Pseudozyma flocculosa* | XP_007878543.1 |
| VpalFTSP-like | fungus | Basidiomycota | *Violaceomyces palustris* | PWN49522.1 |
| MlarFTSP-like | fungus | Basidiomycota | *Melampsora laricis* | XP_007412896.1 |
| CqueFTSP-like | fungus | Basidiomycota | *Cronartium quercuum* | KAG0144215.1 |
| PsorFTSP-like | fungus | Basidiomycota | *Puccinia sorghi* | KNZ54183.1 |
| MphaFTSP-like | fungus | *Ascomycota* | *Macrophomina phaseolina* | KAH7061243.1 |
| MmicFTSP-like | fungus | *Ascomycota* | *Microthyrium microscopicum* | KAF2672361.1 |
| ArabFTSP-like | fungus | *Ascomycota* | *Ascochyta rabiei* | XP_038802689.1 |
| IdesFTSP-like | fungus | *Ascomycota* | *Ilyonectria destructans* | KAH7022408.1 |
| lsp.FTSP-like | fungus | *Ascomycota* | *lyonectria sp. MPI-CAGE-AT-0026* | KAH6989381.1I |
| ChubFTSP-like | fungus | *Ascomycota* | *Cylindrodendrum hubeiense* | KAF7547199.1 |
| LtheFTSP-like | fungus | *Ascomycota* | *Lasiodiplodia theobromae* | KAF9637446.1 |
| DestFTSP-like | fungus | *Ascomycota* | *Dactylonectria estremocensis* | KAH7163591.1 |
| ToliFTSP-like | fungus | *Ascomycota* | *Thelonectria olida* | KAH6887612.1 |
| NparFTSP-like | fungus | *Ascomycota* | *Neofusicoccum parvum UCRNP2* | EOD52258.1 |
| DereFTSP-like | fungus | *Ascomycota* | *Diaporthe eres* | KAI7782618.1 |
| DamyFTSP-like | fungus | *Ascomycota* | *Diaporthe amygdali* | XP_052992429.1 |
| DcitFTSP-like | fungus | *Ascomycota* | *Diaporthe citri* | XP_043017518.1 |
| AsclFTSP-like | fungus | *Ascomycota* | *Aspergillus sclerotiicarbonarius CBS 121057* | PYI02525.1 |
| AhanFTSP-like | fungus | *Ascomycota* | *Aspergillus hancockii* | KAF7593598.1 |
| AcorFTSP-like | fungus | *Ascomycota* | *Aspergillus coremiiformis* | KAE8354272.1 |
| Xsp.FTSP-like | fungus | *Ascomycota* | *Xylariaceae sp. FL0804* | KAI0477078.1 |
| NmosFTSP-like | fungus | *Ascomycota* | *Neoarthrinium moseri* | KAI1841522.1 |
| SbruFTSP-like | fungus | *Ascomycota* | *Sphaerosporella brunnea* | KAA8895541.1 |
| Nsp.FTSP-like | fungus | *Ascomycota* | *Neopestalotiopsis sp. 37M* | KAF3023128.1 |
| NclaFTSP-like | fungus | *Ascomycota* | *Neopestalotiopsis clavispora* | KAF7538191.1 |
| PsubFTSP-like | fungus | *Ascomycota* | *Penicillium subrubescens* | OKP11508.1 |
| Isp.FTSP-like | fungus | *Ascomycota* | *Ilyonectria sp. MPI-CAGE-AT-0026* | KAH6989381.1 |
| FantFTSP-like | fungus | *Ascomycota* | *Fusarium anthophilum* | KAF5236321.1 |
| FphyFTSP-like | fungus | *Ascomycota* | *Fusarium phyllophilum* | KAF5540202.1 |
| FmexFTSP-like | fungus | *Ascomycota* | *Fusarium mexicanum* | KAF5545342.1 |
| FnapFTSP-like | fungus | *Ascomycota* | *Fusarium napiforme* | KAF5546222.1 |
| FpseudoaFTSP-like | fungus | *Ascomycota* | *Fusarium pseudoanthophilum* | KAF5590895.1 |
| FpseudocFTSP-like | fungus | *Ascomycota* | *Fusarium pseudocircinatum* | KAF5596201.1 |
| FdenFTSP-like | fungus | *Ascomycota* | *Fusarium denticulatum* | KAF5666575.1 |
| FcirFTSP-like | fungus | *Ascomycota* | *Fusarium circinatum* | KAF5682859.1 |
| FproFTSP-like | fungus | *Ascomycota* | *Fusarium proliferatum* | KAG4280898.1 |
| FxylFTSP-like | fungus | *Ascomycota* | *Fusarium xylarioides* | KAG5751922.1 |
| FoxyFTSP-like | fungus | *Ascomycota* | *Fusarium oxysporum f. sp. rapae* | KAG7414751.1 |
| FPh1FTSP-like | fungus | *Ascomycota* | *Fusarium sp. Ph1* | KAI8667133.1 |
| FLHS141FTSP-like | fungus | *Ascomycota* | *Fusarium sp. LHS14.1* | KAI8717640.1 |
| FsolFTSP-like | fungus | *Ascomycota* | *Fusarium solani* | KAJ3464210.1 |
| FverFTSP-like | fungus | *Ascomycota* | *Fusarium verticillioides* | RBQ95317.1 |
| FdupFTSP-like | fungus | *Ascomycota* | *Fusarium duplospermum* | RSL60227.1 |
| FAF-6FTSP-like | fungus | *Ascomycota* | *Fusarium sp. AF-6* | RSL72022.1 |
| FfloFTSP-like | fungus | *Ascomycota* | *Fusarium floridanum* | RSL78008.1 |
| FoliFTSP-like | fungus | *Ascomycota* | *Fusarium oligoseptatum* | RSM07097.1 |
| FambFTSP-like | fungus | *Ascomycota* | *Fusarium ambrosium* | RSM17478.1 |
| FodoFTSP-like | fungus | *Ascomycota* | *Fusarium odoratissimum NRRL 54006* | XP_031059164.1 |
| FsubFTSP-like | fungus | *Ascomycota* | *Fusarium subglutinans* | XP_036544457.1 |
| FmanFTSP-like | fungus | *Ascomycota* | *Fusarium mangiferae* | XP_041687096.1 |
| FkerFTSP-like | fungus | *Ascomycota* | *Fusarium keratoplasticum* | XP_052912261.1 |
| FfalFTSP-like | fungus | *Ascomycota* | *Fusarium falciforme* | XP_053009558.1 |

**Supplementary Table 4. Proteins from a *Nicotiana benthamiana* cDNA library screened by yeast two-hybrid (Y2H) using BtFTSP1 as a bait**

| **GenBank Accession** | **Annotation** | **Number of colonies** |
| --- | --- | --- |
| NP_001312705.1 | cytochrome b6-f complex iron-sulfur subunit 2 | 4 |
| ABB30150.1 | ferredoxin | 3 |
| XP_016449404.1 | ribulose bisphosphate carboxylase small chain S41 | 2 |
| XP_016459901.1 | lysine-specific demethylase JMJ25-like | 1 |
| XP_016458783.1 | plasminogen activator inhibitor 1 RNA-binding protein-like | 1 |

**Supplementary Table 5. Primers used in this study**

| **Genes** | **Forward primer (5’-3’)** | | Reverse primer (5’-3’) | |  |  |
| --- | --- | --- | --- | --- | --- | --- |
| **Primers used in qPCR** | |  |  |  |  |  |
| *Nicotiana tabacum tubulin* | AAGTACATGGCTTGCTGCCT | | ATCAATGCGCGAGAAGACCT | |  |  |
| *Bemisia tabaci actin* | TCTTCCAGCCATCCTTCTTG | | CGGTGATTTCCTTCTGCATT | |  |  |
| *NtFD1* | ATGGCCAGTATTTCAGGTACC | | CCTCAGCTTGGTCAAGAATG | |  |  |
| *BtFTSP1* | ATCGTCTGTCCTAGCACGC | | TTATGCCGGACTACCCCCAC | |  |  |
| *BtFTSP2* | CAACCTGAAAGACTGCTACTC | | AAGTGCCATTGATGAGATGCT | |  |  |
| *BtFTSP3* | CATCCCAATGCCGATTATATG | | CTTATATGATGGCGGTTTGTC | |  |  |
| *BtFTSP4* | TTCACACCTTACAAGAGTAGC | | ACACTAAATCCTCCTTCTTCG | |  |  |
| *Nicotiana tabacum* *cytochrome b6-f complex iron-sulfur* | TGGAGAATGATGGAACACTTG | | CAGGTCCTCTAACAACTCTTC | |  |  |
| *Nicotiana tabacum* *ribulose bisphosphate* | CTTCCTCAGTTATGTCCTCAG | | AGTGTCTCGTACTTCTTCTTG | |  |  |
| *Nicotiana tabacum* *lysine-specific demethylase* | GCTTAGAGATGGTCACCTTAA | | CCTCCGTTATCCTTAGATTCC | |  |  |
| *Nicotiana tabacum plasminogen activator inhibitor* | GACACCAATAAGGATTCTACTG | | CCTTCCTCTTCTCTTCCATTA | |  |  |
| **Primers used in double stranded RNA synthesis** | | | | | | |
| *GFP* | TAATACGACTCACTATAGGGAGAATGAGTAAAGGAGAAGAACTTTTC | | TAATACGACTCACTATAGGGAGATTTGTATAGTTCATCCATGCCATGT | |  |  |
| *BtFTSP1* | TAATACGACTCACTATAGGGGGGCAAAGATGAAGGCAAAG | | TAATACGACTCACTATAGGGTTATGCCGGACTACCCCCAC | |  |  |
| **Primers used in binary vector construction** | | | | | | |
| *GFP-BtFTSP1* | ACGAGCTGTACAAGGGTACCATGGGCAAAGATGAAGGCAAAG | | GCGGACTCTAGTTCATCTAGATTATGCCGGACTACCCCCAC | |  |  |
| *BtFTSP1-Flag/Myc/mCherry* | CGACGACAAGACCGTCACCATGGGCAAAGATGAAGGCAAAG | | GAGGAGAAGAGCCGTCGTGCCGGACTACCCCCACCTT | |  |  |
| *NtFD1-Flag/Myc/mCherry* | CGACGACAAGACCGTCACCATGGCCAGTATTTCAGGTACC | | GAGGAGAAGAGCCGTCGGCCAGTGAGCTCCTCCTCC | |  |  |
| *NtFD1- Myc* | CGACGACAAGACCGTCACCATGGATAAATCACCTAATCCCGCCG | | GAGGAGAAGAGCCGTCGATTGCTGTTCGGTGGTACCTT | |  |  |
| **Primers used in Y2H vector construction** | | | | | | |
| *AD-BtFTSP1* | GTACCAGATTACGCTCATATGGGCAAAGATGAAGGCAAAGG | | CAGCTCGAGCTCGATGGATCCTTATGCCGGACTACCCCCAC | |  |  |
| *BD-BtFTSP1* | TCAGAGGAGGACCTGCATATGGGCAAAGATGAAGGCAAAGG | | CCGCTGCAGGTCGACGGATCCTTATGCCGGACTACCCCCAC | |  |  |
| *AD-BtFTSP1/N-terminus* | GTACCAGATTACGCTCATATGGGCAAAGGTGGTAAAAAAGGTG | | CAGCTCGAGCTCGATGGATCCCGGGTTGCACGTGTCAGTGCCA | |  |  |
| *AD-BtFTSP1/C-terminus* | GTACCAGATTACGCTCATATGGGCGGCGGTAATAAAAAGAAAG | | CAGCTCGAGCTCGATGGATCCTTATGCCGGACTACCCCCACCT | |  |  |
| *BD-NtFD1* | TCAGAGGAGGACCTGCATATGGCCAGTATTTCAGGTACC | | CCGCTGCAGGTCGACGGATCCTTAGCCAGTGAGCTCCTCCTCC | |  |  |
| *BD-NtFD1/N-terminus* | TCAGAGGAGGACCTGCATATGGCCAGTATTTCAGGTACC | | CCGCTGCAGGTCGACGGATCCTCCAGCACAAGAAGAGCAAGA | |  |  |
| *BD-NtFD1/C-terminus* | TCAGAGGAGGACCTGCATATGAAAGCCATACCAAATGTTGGG | | CCGCTGCAGGTCGACGGATCCTTAGCCAGTGAGCTCCTCCTCC | |  |  |
| *AD-NtFD1/N-terminus* | GTACCAGATTACGCTCATATGGCCAGTATTTCAGGTACC | | CAGCTCGAGCTCGATGGATCCCATGCAAGTAATCCTACCAC | |  |  |
| *AD-NtFD1/C-terminus* | GTACCAGATTACGCTCATATGGCCAGTTACAAAGTGAAGC | | CAGCTCGAGCTCGATGGATCCTTAGCCAGTGAGCTCCTCCTCC | |  |  |
| *AD-MmFTSP* | GTACCAGATTACGCTCATATGGATAAATCACCTAATCCCGCCG | | CAGCTCGAGCTCGATGGATCCTCAATTGCTGTTCGGTGGTACC | |  |  |
| *AD-MmFTSP/N-terminus* | GTACCAGATTACGCTCATATGGATAAATCACCTAATCCCGCCG | | CAGCTCGAGCTCGATGGATCCATCAGCTGCAGGAGCGGCTGT | |  |  |
| *AD-MmFTSP/C-terminus* | GTACCAGATTACGCTCATATGCCTACAACAGGTACTGGCACA | | CAGCTCGAGCTCGATGGATCCTCAATTGCTGTTCGGTGGTACC | |  |  |
| **Primers used in BIFC vector construction** | | | |  | |  |
| *NY-NtFD1* | ATCGAGGACTCCGGAGTCGACATGGCCAGTATTTCAGGTACC | | GATCGGGGAAATTCGAGCTCTTAGCCAGTGAGCTCCTCCTCC | |  |  |
| *CY-NtFD1* | CTGTACAAGTCCGGAGTCGACATGGCCAGTATTTCAGGTACC | | GATCGGGGAAATTCGAGCTCTTAGCCAGTGAGCTCCTCCTCC | |  |  |
| *NY-BtFTSP1* | ATCGAGGACTCCGGAGTCGACATGGGCAAAGATGAAGGCAAAG | | GATCGGGGAAATTCGAGCTCTTATGCCGGACTACCCCCAC | |  |  |
| *CY-BtFTSP1* | CTGTACAAGTCCGGAGTCGACATGGGCAAAGATGAAGGCAAAG | | GATCGGGGAAATTCGAGCTCTTATGCCGGACTACCCCCAC | |  |  |
| **Primers used in LUC vector construction** | | | | | | |
| *NLUC- NtFD1* | GTACGCGTCCCGGGGCGGTACCATGGGCAAAGATGAAGGCAAAG | | GACGCGTACGAGATCTGGTCGACGCCAGTGAGCTCCTCCTCC | |  |  |
| *CLUC- BtFTSP1* | GTACGCGTCCCGGGGCGGTACCATGGGCAAAGATGAAGGCAAAG | | GAACGAAAGCTCTGCAGGTCGACTTATGCCGGACTACCCCCAC | |  |  |
| **Primers used in prokaryotic expression** | | | | |  |  |
| *PET-28a-BtFTSP1-GFP* | CAGCAAATGGGTCGCGGATCCATGGTGAGCAAGGGCGAG | | GTGGTGGTGGTGGTGCTCGAGTGCCGGACTACCCCCAC | |  |  |
| *PET-28a-GFP* | CAGCAAATGGGTCGCGGATCCATGGTGAGCAAGGGCGAGG | | GTGGTGGTGGTGGTGCTCGAGTGTACAGCTCGTCCATGCCGA | |  |  |
| *pGEX-6p-1-BtFTSP1* | TTCCAGGGGCCCCTGGGATCCATGGGCAAAGATGAAGGCAAAG | | GTCACGATGCGGCCGCTCGAGTTATGCCGGACTACCCCCAC | |  |  |

Data S1.

Sequences used in this study

Data S2.

Differentially expressed genes between *Nicotiana tabacum* infested by ds*GFP*- and dsB*tFTSP1*- treated *Bemisia tabaci*
